# Temperature‐Responsive Near‐Infrared Emission Enabled by Reversible π‐Umpolung with an Alkenyl‐Strapped Diarylboryl Unit

**DOI:** 10.1002/anie.202523338

**Published:** 2026-01-24

**Authors:** Satoru Kitamura, Mika Sakai, Shigehiro Yamaguchi

**Affiliations:** ^1^ Department of Chemistry Graduate School of Science and Integrated Research Consortium on Chemical Sciences (IRCCS) Nagoya University Furo, Chikusa Nagoya 464‐8602 Japan; ^2^ Research Center of Materials Science (RCMS) Nagoya University Furo, Chikusa Nagoya 464‐8602 Japan; ^3^ Institute of Transformative Bio‐Molecules (WPI‐ITbM) Nagoya University Furo, Chikusa Nagoya 464‐8601 Japan

**Keywords:** Boron, Fluorescence, Temperature response, π‐electron systems, π‐umpolung

## Abstract

Coordination of a Lewis base to a tricoordinate boryl group generates a tetracoordinate species, thereby inverting the electronic character of the boryl substituents from electron‐accepting to electron‐donating. Utilizing this π‐umpolung strategy, we report temperature‐responsive fluorophores that emit in the near‐infrared (NIR) region. To achieve reversible π‐umpolung, we designed a diarylboryl unit in which two aryl rings are tethered by an alkenyl linker. This alkenyl‐strapped scaffold engages in a weak olefin–borane interaction and undergoes frustrated Lewis pair (FLP)‐type addition even with bulky neutral Lewis bases such as tricyclohexylphosphine (PCy_3_). When boryl groups are installed at both termini of 4,7‐di(2‐thienyl)‐2,1,3‐benzothiadiazole, coordination of PCy_3_ induces pronounced red‐shifts in the emission spectra. In polar acetonitrile, the emission maximum reaches 732 nm, entering the NIR region. This red shift arises from the strong σ‐donating character of the resulting tetracoordinate boron centers, which enhance intramolecular charge transfer (ICT) character in the excited state. Unlike conventional dimesitylborane–fluoride complexes, the FLP‐type adducts exhibit reversible, temperature‐dependent shifts in the dissociation/association equilibrium. Although the solvent polarity influences the equilibrium, modulation of phosphine Lewis basicity enables reversible dissociation even in polar media, allowing this system to display large emission changes spanning the visible to NIR region.

## Graphical abstract

Coordination of a Lewis base switches an electron‐accepting tricoordinate boron center into an electron‐donating tetracoordinate one. An alkenyl‐strapped diphenylthienylboryl π‐system undergoes phosphine‐induced reversible π‐umpolung, where transformation from an acceptor–acceptor–acceptor framework to a donor–acceptor–donor system gives rise to temperature‐responsive fluorescence changes in the red‐to‐NIR region.

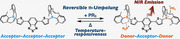

## Introduction

“Reactivity umpolung”, the inversion of polarity at a reactive site, is a fundamental concept in modern synthetic chemistry, enabling transformations that are otherwise difficult to achieve by conventional means.^[^ ^1^ ^]^ From the perspective of functional materials, the ability to switch between electron‐accepting and electron‐donating character within π‐conjugated systems, referred to here as “π‐umpolung”, offers a powerful strategy for tuning electronic and photophysical properties. This concept can be implemented using tricoordinate boron‐based π‐electron systems.

**Figure 1:**
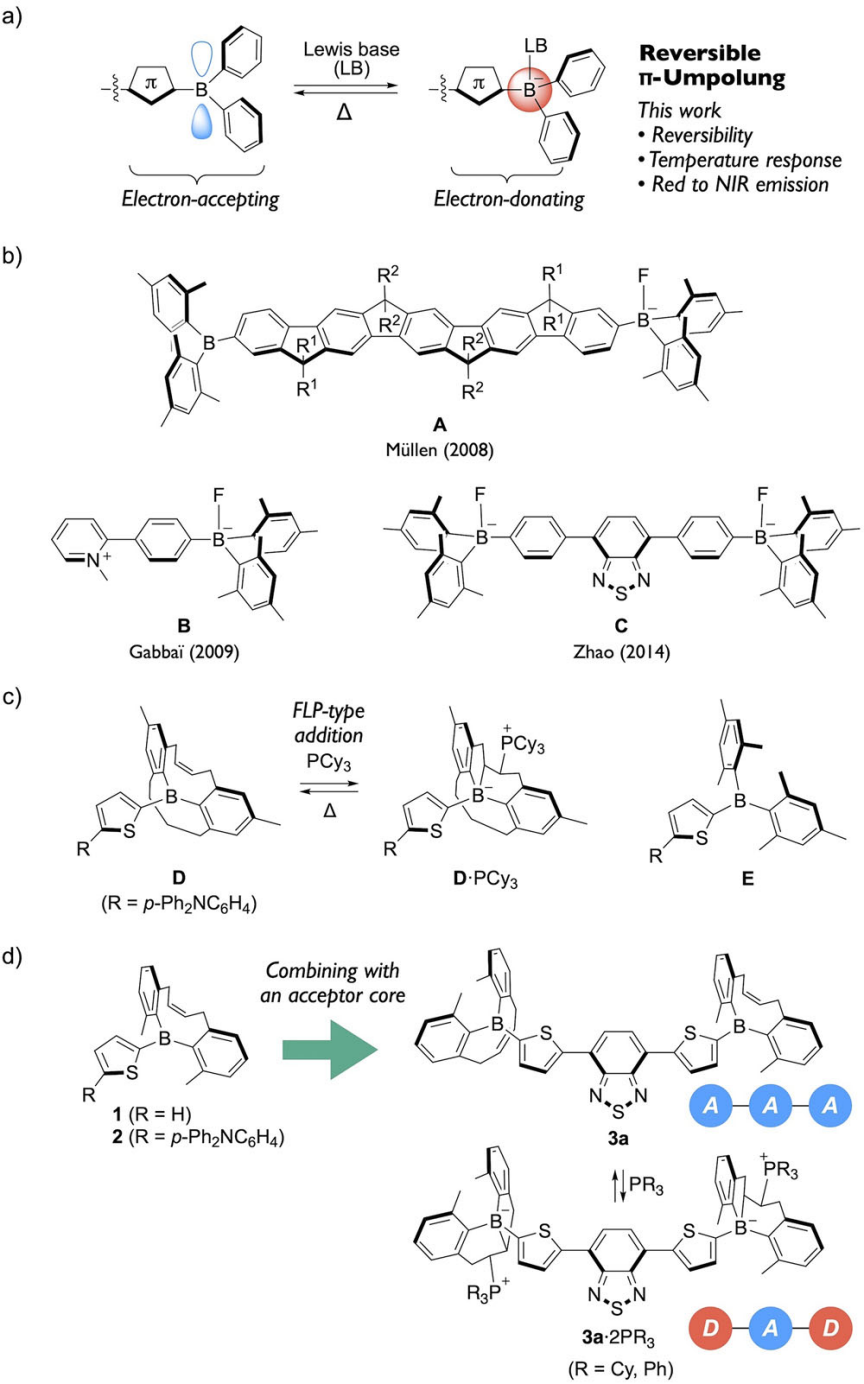
a) Schematic illustration of reversible, stimuli‐responsive π‐umpolung in boron‐containing π‐conjugated frameworks through Lewis base coordination. b) Representative examples of borate‐containing π‐systems **A**–**C**. c) Chemical structures of the doubly strapped diarylboryl compound **D** and its FLP‐type adduct, and the dimesitylboryl compound **E** for comparison. d) Mono‐alkenyl‐strapped diarylboryl derivatives **1**–**3** investigated in this study.

Incorporation of tricoordinate boron, which is isoelectronic with a carbocation, endows π‐conjugated frameworks with strong electron‐accepting character (Figure 1a). Such boron‐containing units are commonly integrated with electron‐donating motifs to construct donor–π–acceptor (D–π–A) or acceptor–π–donor–π–acceptor (A–π–D–π–A) systems, which have been widely explored in diverse applications including nonlinear optics, two‐photon absorption, light‐emitting devices, electron‐transporting materials, and fluorescent probes for bioimaging.^[^ ^2^, ^3^, ^4^, ^5^, ^6^, ^7^, ^8^, ^9^, ^10^, ^11^, ^12^, ^13^ ^]^ Another key feature of boron‐based π‐systems is their ability to coordinate with a variety of Lewis bases, not only anionic species (e.g., fluoride, cyanide),^[^ ^14^, ^15^, ^16^ ^]^ but also neutral bases (e.g., pyridines, phosphines),^[^ ^17^, ^18^, ^19^, ^20^, ^21^, ^22^ ^]^ and intramolecular lone pair‐bearing groups (e.g., CHO, NR_2_, OR, SR, P(Ch)R_2_ (Ch = O, S, Se)).^[^ ^23^, ^24^, ^25^, ^26^, ^27^, ^28^, ^29^, ^30^, ^31^, ^32^, ^33^, ^34^, ^35^, ^36^, ^37^ ^]^ Conversion from a tricoordinate to a tetracoordinate boron center significantly alters the electronic and structural characteristics of the π‐system. In addition to enabling the sensing of anions present in the medium,^[^ ^14^, ^16^, ^38^, ^39^, ^40^ ^]^ this transformation has also proven effective for functional modulations^[^ ^41^, ^42^ ^]^ such as solubility tuning,^[^ ^43^ ^]^ self‐assembly behavior,^[^ ^44^, ^45^ ^]^ and self‐healing properties.^[^ ^46^, ^47^, ^48^, ^49^, ^50^ ^]^

Particularly significant is the switch in electronic character that accompanies this conversion. The transition from a tricoordinate borane to a tetracoordinate borate not only disrupts p–π^∗^ conjugation by eliminating the vacant p orbital on boron, but also enhances the electron‐donating capacity of the resulting borate‐substituted π‐frameworks. Pioneering studies reported zwitterionic molecules bearing a tetracoordinate borate and a cationic moiety at both ends of a π‐conjugated framework, which exhibited intramolecular charge transfer (ICT) characteristics.^[^ ^51^, ^52^ ^]^ This π‐umpolung effect has been leveraged to modulate photophysical properties of boronic ester‐ or dimesityboryl‐substituted compounds through post‐coordination with Lewis bases (Figure 1b).^[^ ^53^, ^54^, ^55^, ^56^, ^57^, ^58^, ^59^, ^60^, ^61^, ^62^, ^63^, ^64^, ^65^, ^66^, ^67^, ^68^, ^69^, ^70^, ^71^, ^72^, ^73^, ^74^, ^75^, ^76^ ^]^ For instance, Müllen and co‐workers reported ladder‐type pentaphenylene derivative **A** featuring dimesitylboryl groups at both termini, where mono‐fluoride coordination induced red‐shifted absorption and emission via increased donor character on one side of the π‐framework.^[^ ^56^ ^]^ Gabbaï and co‐workers reported compound **B**, which combines a tetracoordinate borate moiety with an electron‐accepting pyridinium scaffold.^[^ ^59^ ^]^ Zhao and co‐workers reported compound **C**, comprising dimesitylboryl groups at both termini of a benzothiadiazole‐based π‐framework, where fluoride coordination resulted in red‐shifts in the absorption spectrum.^[^ ^63^ ^]^ However, these previous examples have relied exclusively on anionic Lewis bases, most commonly fluoride, which inherently limit reversibility and environmental responsiveness. While several molecules bearing tricoordinate boranes as electron‐accepting groups are reported to exhibit NIR emission in highly polar solvents,^[^ ^77^, ^78^, ^79^ ^]^ spectral changes achieved through Lewis base coordination are typically confined to the visible region; examples demonstrating modulation in the NIR region remain scarce, despite the importance of NIR emission for bioimaging and photonic applications. Overcoming these limitations requires the design of more sophisticated boron‐based molecular architectures that enable reversible, stimuli‐responsive π‐umpolung with tunable NIR emission (Figure 1a).

In this context, we previously developed a prototype diarylboryl scaffold in which two aryl substituents are bridged by dual linkers, one bearing an alkenyl group (Figure 1c).^[^ ^72^ ^]^ This structure sterically protects the tricoordinate boron center while allowing a weak intramolecular interaction between boron and the olefin moiety. Upon addition of bulky neutral phosphines such as PCy_3_, a frustrated Lewis pair (FLP)‐type addition occurs at the olefin, affording a tetracoordinate boron center. In a D–π–A‐type molecule (compound **D**) incorporating this boryl unit and an electron‐donating group, this FLP‐type reaction was reversible and accompanied by a notable fluorescence change in the blue region. This behavior stands in marked contrast to that of dimesitylboryl‐substituted analogue **E**, which remains unresponsive to PCy_3_. Nevertheless, the laborious synthesis of the double‐strapped scaffold posed a significant barrier to further exploration.

To overcome this challenge, we have now developed a mono‐alkenyl‐strapped diarylboryl group that is both synthetically accessible and structurally versatile. A series of π‐conjugated molecules incorporating this unit were synthesized (Figure 1d), including a thienyl derivative (compound **1**) and an analogue of compound **D** (compound **2**), enabling direct evaluation of the structural impact of the mono‐strapping design. Extending beyond conventional D–π–A frameworks, we further incorporated this diarylboryl unit into an electron‐accepting π‐core to construct an acceptor–acceptor–acceptor (A–A–A)‐type system. We hypothesized that, upon FLP‐type addition, this framework would be transformed into a donor–acceptor–donor (D–A–D)‐type system, thereby amplifying the π‐umpolung effect and enabling red‐shifted emission. To test this concept, we employed a benzothiadiazole‐based core to afford **3a**. This symmetrically extended molecule demonstrated distinct red‐shifts in its emission spectra into the NIR region upon phosphine addition, along with reversible temperature‐dependent switching behavior. The detailed synthesis, photophysical analysis, and computational insights into this unprecedented responsive π‐umpolung system are discussed herein.

## Results and Discussion

**Scheme 1:**
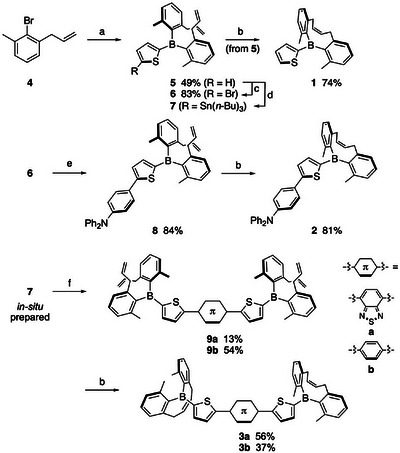
Synthesis of **1**–**3**. *Reagents and conditions*: a) i) Mg (1.2 equiv), THF, 0 °C; ii) potassium thiophene‐2‐trifluoroborate, THF, rt; b) 2nd generation Grubbs cat., CH_2_Cl_2_, rt; c) i) *n*‐BuLi, THF, –78 °C; ii) 1,2‐dibromo‐1,1,2,2‐tetrachloroethane, –78 °C to rt; d) i) *n*‐BuLi, THF, –78 °C; ii) *n‐*Bu_3_SnCl, –78 °C to rt; e) (*p*‐Ph_2_NC_6_H_4_)SnBu_3_, Pd(PPh_3_)_4_, 1,4‐dioxane, reflux; f) corresponding dibromoarenes, Pd(PPh_3_)_4_, 1,4‐dioxane, microwave or reflux.

The synthesis of mono‐alkenyl‐strapped diarylboryl π‐conjugated compounds was accomplished using bis(2‐allyl‐6‐methylphenyl)(2‐thienyl)borane (**5**) as a key precursor (Scheme 1). This compound was obtained in 49% yield by the reaction of potassium thiophene‐2‐trifluoroborate with 2 equiv of (2‐allyl‐6‐methylphenyl)magnesium bromide.^[^ ^80^ ^]^ Subsequent derivatization of **5** via lithiation followed by bromination and stannylation afforded **6** and **7**, respectively. Kosugi–Migita–Stille cross‐coupling reaction of **6** with (*p*‐Ph_2_N‐substituted phenyl)tributylstannane provided π‐extended compound **8** in 84% yield. Likewise, the reaction of in situ‐generated **7** with dibromobenzothiadiazole or dibromobenzene, furnished **9a** and **9b** in 13% and 54% yields, respectively. The final step involved ring‐closing metathesis in the presence of the 2nd generation Grubbs catalyst^[^ ^81^ ^]^ which produced target compounds, thienyl derivative **1** from **5**, mono‐borylated compound **2** from **8**, and bis‐borylated compound **3a** and **3b** from **9a** and **9b**, respectively. In all cases, the metathesis proceeded in a *cis*‐selective manner, as confirmed by the ^1^H NMR spectra. All mono‐alkenyl‐strapped diarylboryl compounds were stable toward air and moisture to be treated without special precautions.

**Figure 2:**
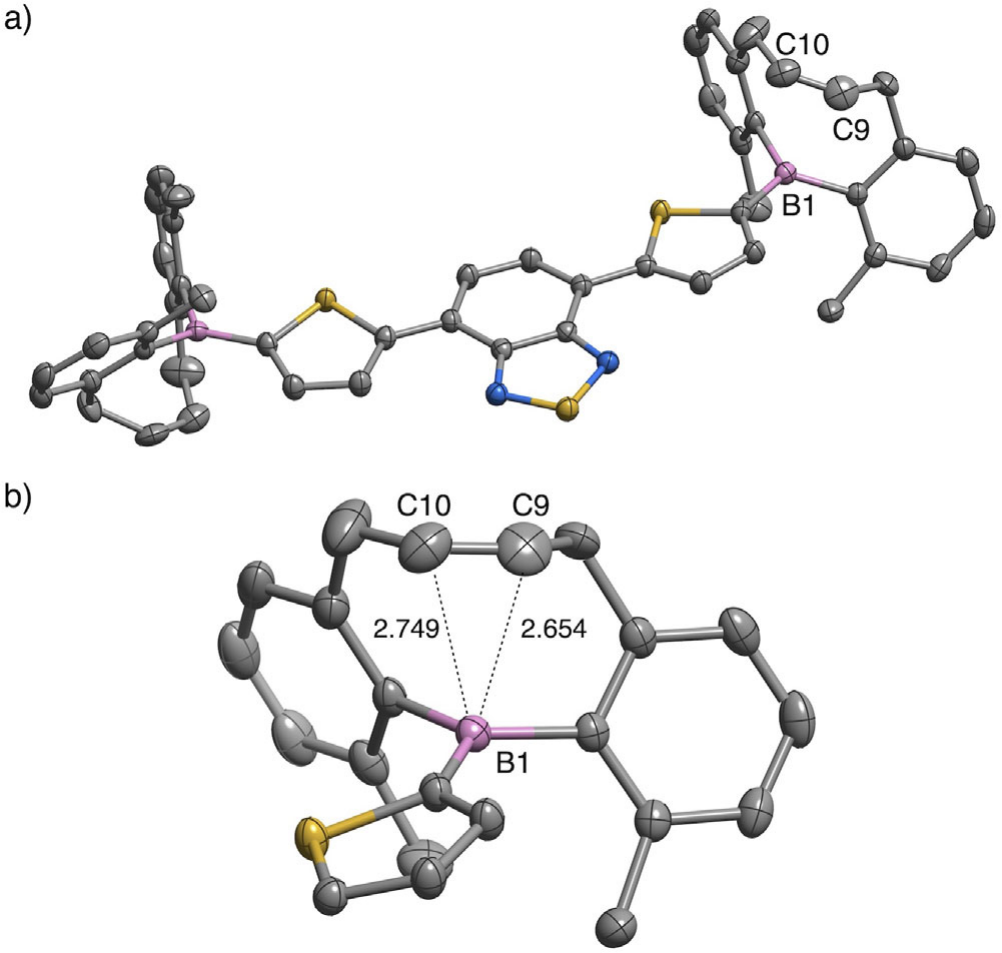
a) *ORTEP* diagram of **3a** (50% probability for thermal ellipsoids). Hydrogen atoms and solvents are omitted for clarity. b) Enlarged view of the area around the boron moiety.

Among these compounds, single‐crystal X‐ray diffraction analyses of **1** and **3a** were performed.^[^ ^82^ ^]^ Crystals suitable for measurement were obtained by slow cooling of a hot ethyl acetate solution for **1**, and by a two‐layer diffusion method using methanol/dichloromethane solutions for **3a** (Figures 2 and S1). In both structures, the boron centers are sterically well protected by the mono‐alkenyl linker and two methyl substituents. Notably, the olefin moieties are positioned above the boron atom with the B···C_olefin_ distances of 2.655 and 2.724 Å in **1** and 2.654 and 2.749 Å in **3a**, which are significantly shorter than the sum of the van der Waals radii of boron and carbon atoms (3.62Å).^[^ ^83^ ^]^ These distances fall within the typical range observed in compounds featuring olefin–borane interactions,^[^ ^72^, ^84^, ^85^, ^86^ ^]^ including compound **D** (B···C_olefin_: 2.724 and 2.786 Å).^[^ ^72^ ^]^

To investigate the electron‐accepting character of the mono‐alkenyl‐strapped diarylboryl group, compound **2** was compared with alkenyl‐ and alkyl‐strapped diarylboryl congener **D** as well as dimesitylboryl analogue **E**.^[^ ^87^ ^]^ Reflecting their D–π–A electronic characteristic, all these compounds exhibited pronounced solvatochromism in their fluorescence spectra, whereas their absorption spectra showed subtle solvent‐dependent changes (Table S1 and Figure S2). The emission maximum wavelength (*λ*_em_) of **2** in CH_2_Cl_2_ was observed at 507 nm, slightly red‐shifted relative to that of **D** (*λ*_em_ = 488 nm).^[^ ^72^ ^]^ Compound **2** displayed a high fluorescence quantum yield of 0.74, accompanied by a single‐exponential decay with a fluorescence lifetime of 2.8 ns (Figure S3). This is in contrast to the fact that **D** has a lower quantum yield of 0.21 with a biexponential decay profile with lifetime components of *τ*_1_ = 0.59 ns and *τ*_2_ = 2.5 ns.^[^ ^72^ ^]^ Given that the short lifetime component in **D** likely arises from the persistence of the borane–olefin interaction in the excited state,^[^ ^72^ ^]^ the absence of such a component in **2** suggests that the borane–olefin interaction within the mono‐alkenyl‐strapped diarylboryl group is not maintained upon excitation, thereby leading to a slightly red‐shifted emission than **D**. However, given the fact that the *λ*_em_ of **2** remains blue‐shifted relative to that of **E** (*λ*_em_ = 512 nm),^[^ ^87^ ^]^ the photo‐dissociated olefin moiety in **2** still seems to affect the electron‐accepting character of the diarylboryl group in the excited state. In this context, the higher fluorescence quantum yield observed for **2** relative to that of **D** can also be attributed by the loss of borane–olefin interaction in the excited state, resulting in a quantum yield closer to that of **E**.

**Figure 3:**
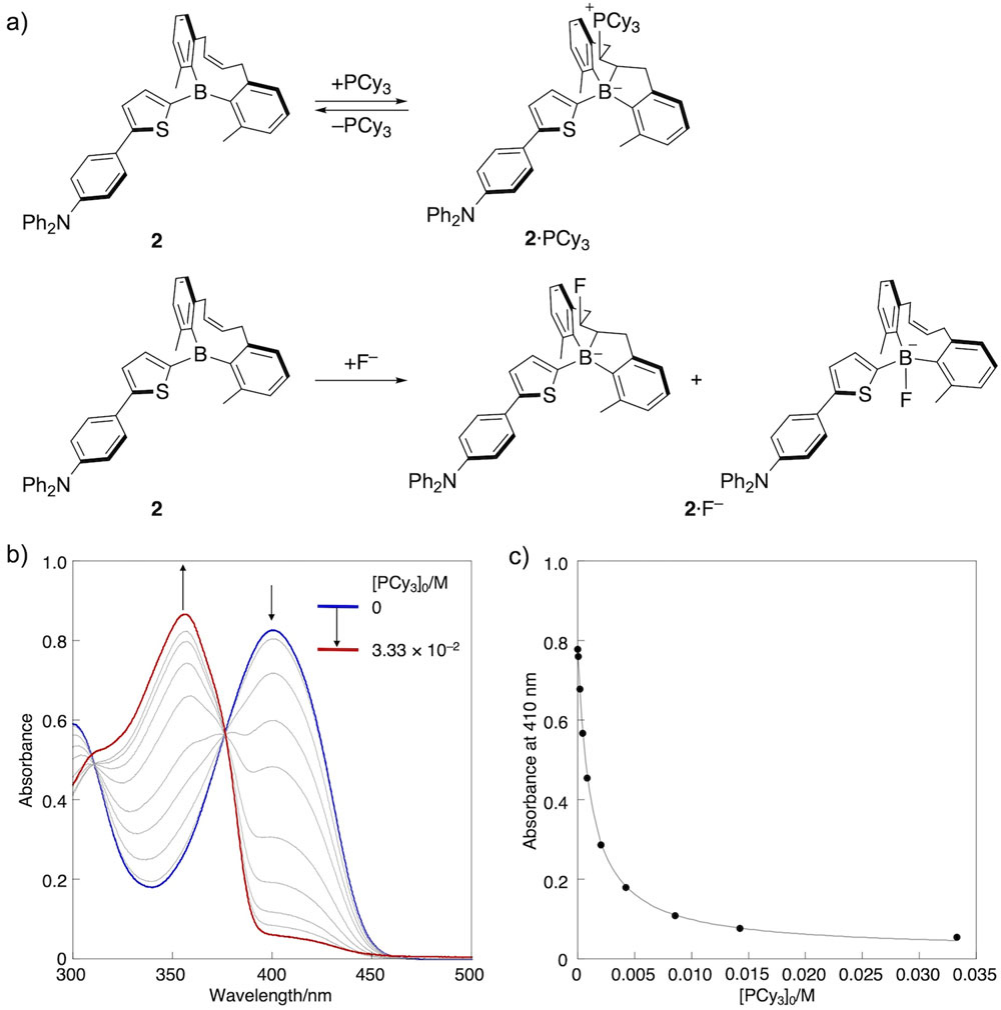
a) Different reaction modes of **2** with PCy_3_ and fluoride. b) UV–vis absorption spectral changes upon addition of PCy_3_ to a toluene solution of **2** (2.57 × 10^−5^ M) and c) plots and titration curve of **2** at 410 nm (*R* ^2^ = 0.99907) at 25 °C.

The influence of the mono‐alkenyl‐strapped structure in **2** on its reactivity toward FLP‐type addition was examined by treating **2** with bulky phosphine PCy_3_. Upon addition of PCy_3_ to a CDCl_3_ solution of **2**, the ^11^B NMR spectrum displayed a significant up‐field shift from a broad signal at 61.9 ppm to a sharp signal at –8.6 ppm. Correspondingly, the ^31^P NMR spectrum exhibited a new signal at 28.8 ppm, alongside the signal of unreacted PCy_3_ at 11.4 ppm (Figure S11). These chemical shifts are comparable to those observed for **D·**PCy_3_ adduct in CDCl_3_ (^11^B NMR: –8.7 ppm; ^31^P NMR: 28.7 ppm) (Figure S13), indicating that even in the mono‐alkenyl‐strapped structure, a bulky, neutral Lewis base can engage in the FLP‐type addition (Figure 3a).

UV–vis absorption spectroscopy was further employed to quantitatively compare the reactivity of the alkenyl‐strapped diarylboryl moieties in **2** and **D** toward the FLP‐type addition. Upon addition of PCy_3_ to a toluene solution of **2**, the absorption band centered around 400 nm decreased in intensity, while a new blue‐shifted band emerged at *λ*_abs_ = 357 nm, accompanied by the appearance of an isosbestic point (Figure 3b,c). The association constant of **2** with PCy_3_ in toluene at 25 °C was determined to be 8.8 × 10^2^ M^−1^, which is smaller than the value observed for **D** with PCy_3_ (2.3 × 10^3^ M^−1^).^[^ ^72^ ^]^ This order is consistent with the calculated difference in Gibbs free energy (ΔΔ*G*) for the addition reactions, obtained at the GD3‐M06‐2X/6‐311G(d) level of theory including toluene as a solvent using the polarizable continuum model (PCM). The computational results indicate that the formation of the **2·**PCy_3_ adduct is thermodynamically less favorable than that of the **D·**PCy_3_ adduct (Figure S15).

In contrast to the reaction mode observed with bulky phosphines, a distinct reaction pathway emerged when a small anionic nucleophile, such as a fluoride ion was employed. A solution of **2** in CDCl_3_ (including 10%THF) containing excess tetra(*n*‐butyl)ammonium fluoride (TBAF) as the fluoride source exhibited a broad signal at 4.1 ppm and a sharp signal at –8.4 ppm in ^11^B NMR spectrum. These chemical shifts are comparable to those of the dimesitylboryl–fluoride adduct **E·**F^–^ (4.2 ppm) and the **D·**F^–^ adduct (–8.4 ppm), the latter formed exclusively through the FLP‐type addition (Figure S14). These observations demonstrate that the small fluoride ion can directly coordinate to the boron center in the mono‐alkenyl‐strapped boryl group in **2**, in sharp contrast to the behavior of **D**, and likely approaches from the backside of the alkenyl‐strapped moiety (Figure 3a).

While diarylboryl groups are typically combined with electron‐donating π‐conjugated framework to capitalize on their electron‐accepting character, we became interested in integrating them with an electron‐accepting π‐conjugated framework to construct an A–A–A‐type π‐system. From this perspective, we employed a 4,7‐di(2‐thienyl)‐2,1,3‐benzothiadiazole core and introduced the mono‐alkenyl‐strapped diarylboryl groups at both termini to afford compound **3a**. For comparison, 1,4‐di(2‐thienyl)benzene‐based analogue **3b** was also investigated.

To assess the impact of the π‐umpolung on the photophysical properties, we first examined the behavior of **3b** (Figure S4). In toluene, this compound exhibited absorption and emission maxima at around 415 nm (shoulder) and 435 nm, respectively, and showed only subtle solvatochromic shifts in both spectra (Table S2 and Figure S4), reflecting the absence of an electron‐donating moiety in the π‐framework. Upon addition of excess PCy_3_ (∼40 equiv) to a toluene solution of **3b** (1.64 × 10^−5^ M), a slight red shift was observed in the tail of the absorption spectrum, while the emission spectrum evolved into a broad band centered around 470 nm. However, further addition of a larger excess of PCy_3_ resulted in blue shifts in both absorption and emission, shifting them to wavelengths shorter than the original bands (Figure S5). Similar biphasic behavior has been reported for other π‐conjugated compounds bearing dimesitylboryl groups at both termini upon treatment with fluoride ions.^[^ ^38^, ^56^, ^69^, ^71^ ^]^ This spectral response can be rationalized by considering that, under conditions where only one of the two terminal boryl groups forms an adduct with PCy_3_, ICT character emerges in the excited state. This arises from the enhanced electron‐donating ability of the resulting borylthienyl moiety, imparting asymmetry to the π‐system.

**Figure 4:**
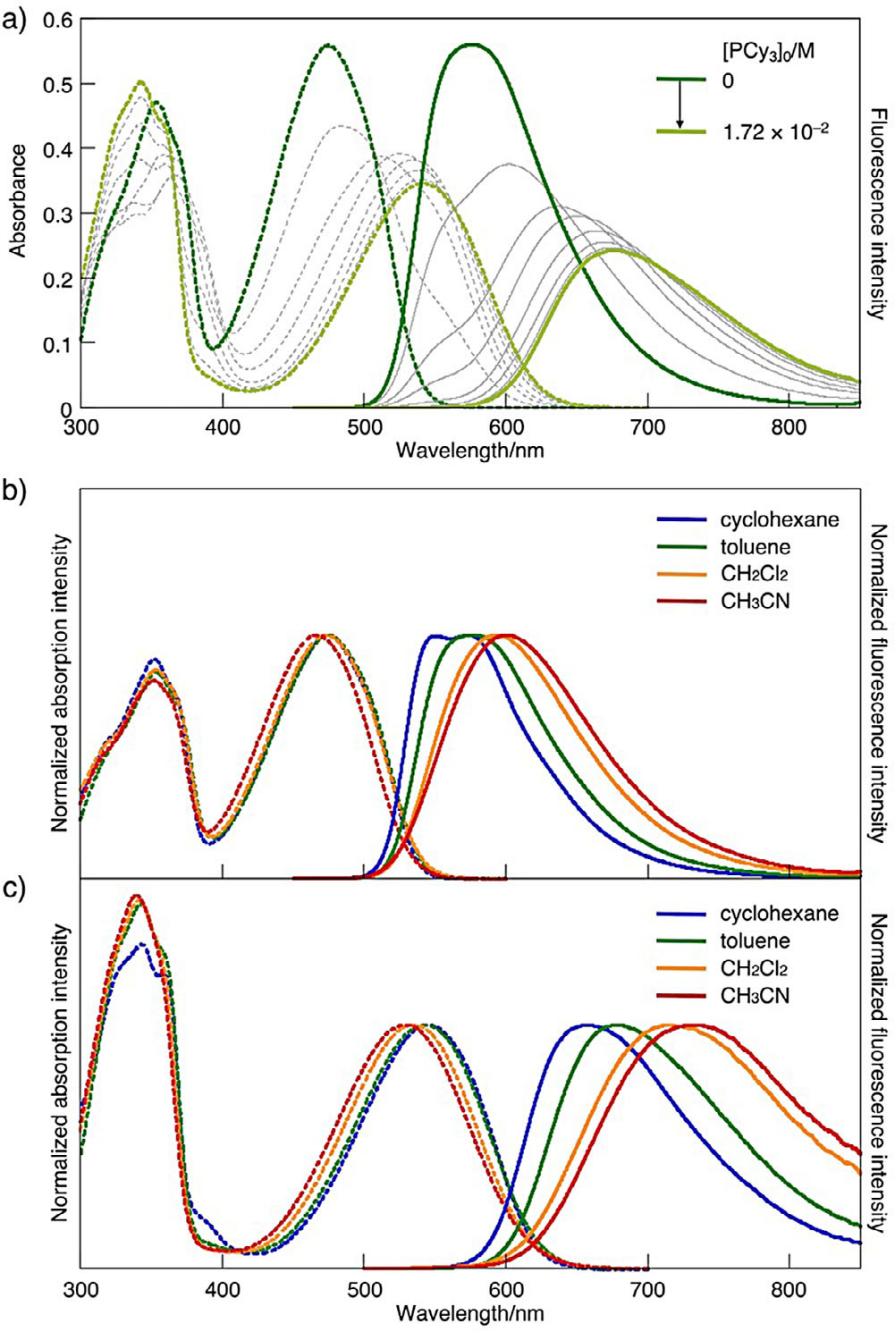
a) UV–vis absorption (dotted line) and fluorescence (solid line) (*λ*_ex_ = 365 nm) spectral changes upon addition of PCy_3_ to a toluene solution of **3a** (1.77 × 10^−5^ M) at 25 °C. UV–vis absorption (dotted line) and emission (solid line) spectra of b) **3a** (*λ*_ex_ = 430 nm) and c) **3a**·2PCy_3_, generated in situ from **3a** with excess PCy_3_, (*λ*_ex_ = 350 nm) in various solvents at 25 °C: [**3a**]_0_ = 2.6 × 10^– 5^ M, [PCy_3_]_0_ = saturated in cyclohexane, 7.6 × 10^−2^ M in toluene, 1.0 × 10^−2^ M in CH_2_Cl_2_, 5.0 × 10^−3^ M in CH_3_CN.

A more pronounced effect was observed in benzothiadiazole‐containing derivative **3a**. In toluene, **3a** exhibited absorption and emission maxima at 475 nm and 576 nm, respectively (Figure 4a). Upon gradual addition of PCy_3_ to a toluene solution of **3a** (1.77 × 10^−5^ M), the absorption maximum red‐shifted progressively, ultimately reaching 543 nm by addition of excess PCy_3_. The fluorescence spectra exhibited a red‐shift in the maximum wavelength from 576 nm to 675 nm, accompanied by a visible fluorescence color change from yellow to red. The resulting **3a·**2PCy_3_ adduct exhibited pronounced solvatochromism (Table 1 and Figure 4b,c). Specifically, while its absorption maximum showed a slight blue shift with increasing the solvent polarity, the emission maximum exhibited a significant red shift from 657 nm in cyclohexane to 732 nm in acetonitrile. Despite the large Stokes shift of 5207 cm^−1^ observed in acetonitrile, the adduct showed a fluorescence quantum yield of 0.17, which is relatively high for emission in this NIR region. This behavior is in marked contrast to that reported for **C**, which resulted in fluorescence quenching upon addition of excess fluoride ion.^[^ ^63^ ^]^

**Table 1:** Photophysical Properties of **3a** and Its Phosphine Adducts.

Compd.	Solvent	Concentration/M^a)^	λ_abs_/nm^b)^	ε/10^4^ M^−1^cm^−1^	λ_em_/nm^c)^	ν_abs_–ν_em_/cm^−1^	Φ_F_ ^d)^
**3a**	cyclohexane	–	475	3.22	550	2871	0.90
	toluene	–	475	3.17	576	3692	0.90
	CH_2_Cl_2_	–	473	3.19	593	4278	0.87
	CH_3_CN	–	467	–^e)^	601	4774	0.86
**3a**·2PCy_3_	cyclohexane	[**3a**]_0_ = 2.59 × 10^−5^ [PCy_3_]_0_ saturated	545	–^e)^	657	3128	0.65
	toluene	[**3a**]_0_ = 2.59 × 10^−5^ [PCy_3_]_0_ = 7.64 × 10^−2^	543	1.91	680	3710	0.54
	CH_2_Cl_2_	[**3a**]_0_ = 2.59 × 10^−5^ [PCy_3_]_0_ = 1.02 × 10^−2^	534	2.00	717	4780	0.23
	CH_3_CN	[**3a**]_0_ = 2.60 × 10^−5^ [PCy_3_]_0_ = 5.03 × 10^−3^	530	1.96	732	5207	0.17
**3a**·2PPh_3_	CH_3_CN	[**3a**]_0_ = 2.20 × 10^−5^ [PPh_3_]_0_ = 6.59 × 10^−2^	522	2.02	714	5151	0.24

^a)^ Concentration employed for all photophysical measurements, including absorption spectra, emission spectra, and quantum yield determinations.

^b)^ Only the longest absorption maximum wavelengths are shown.

^c)^ Emission maximum wavelengths upon excitation at *λ*_ex_ = 430 nm for **3a**; *λ*_ex_ = 350 nm for **3a**·2PCy_3_; *λ*_ex_ = 365 nm for **3a**·2PPh_3_.

^d)^ Absolute fluorescence quantum yields determined by a calibrated integrating sphere system with ± 8% error upon internal light source excitation at *λ*_ex_ = 430 nm for **3a**; upon broad band excitation at *λ*_ex_ = 475 nm (filter) for **3a**·2PCy_3_ and **3a**·2PPh_3_.

^e)^ Not determined due to low solubility.

**Figure 5:**
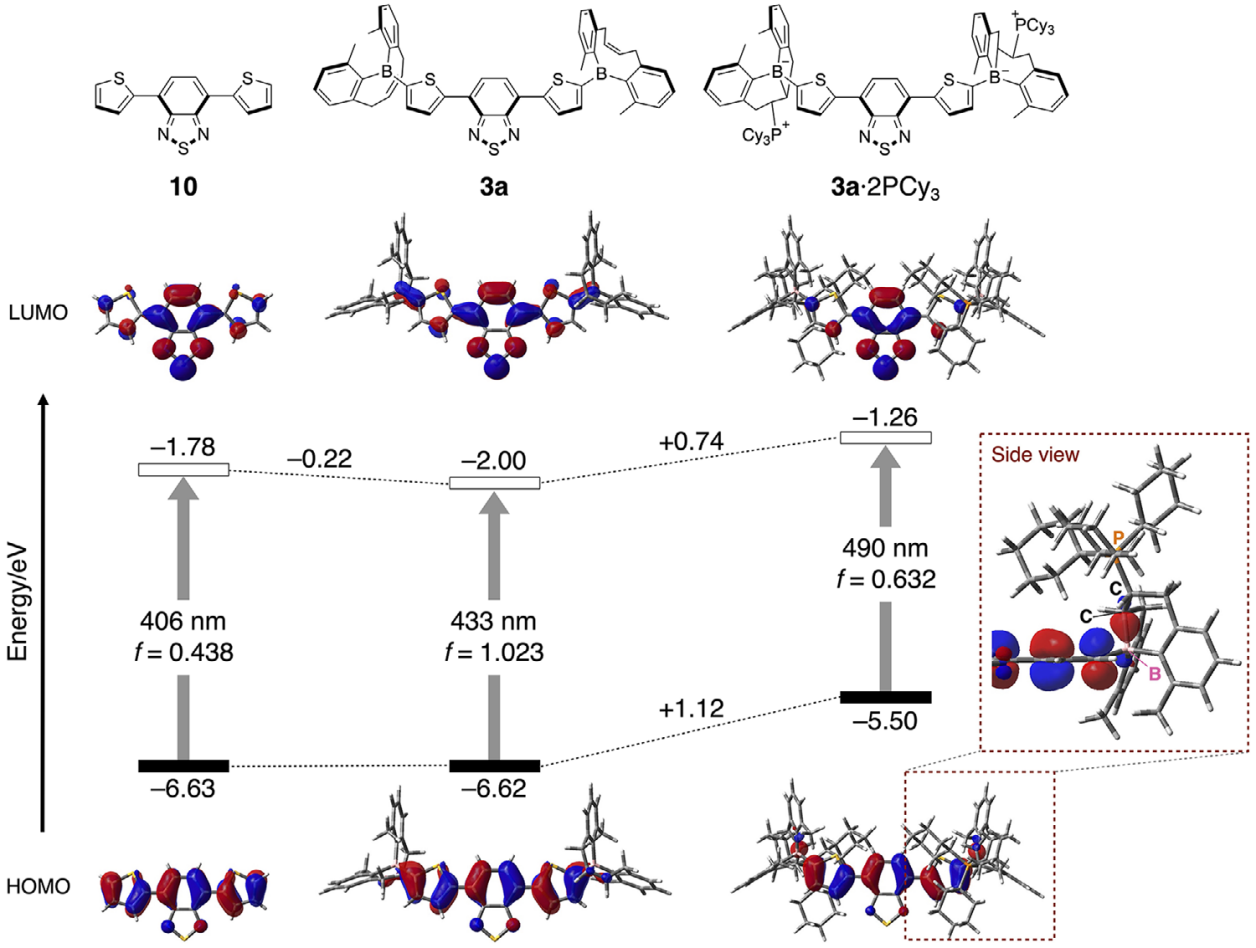
Energy diagrams and Kohn–Sham HOMOs and LUMOs for 4,7‐di(2‐thienyl)‐2,1,3‐benzothiadiazole (**10**), **3a,** and **3a**·2PCy_3_ calculated at the TD‐M06‐2X/6‐31G(d) level of theory.

To elucidate the effect of π‐umpolung induced by PCy_3_ addition, the electronic structures of 4,7‐di(2‐thienyl)‐2,1,3‐benzothiadiazole (**10**), **3a**, and its PCy_3_ adduct **3a·**2PCy_3_ were compared using time‐dependent density functional theory (TD‐DFT) calculation at the M06‐2X/6‐31G(d) level of theory (Figure 5 and Table S3). In going from **10** to **3a**, while the HOMO energy levels remain largely unchanged, the LUMO level is notably decreased. For both compounds, the lowest‐energy absorption arises from a HOMO–LUMO transition. In **3a**, the LUMO extends to the terminal boron atoms, resulting in a 27 nm red shift in the calculated absorption maximum and an increased oscillator strength. These changes highlight the impact of enhanced electron‐accepting character by introducing the boryl group at both termini. Upon formation of the **3a·**2PCy_3_ adduct, both the HOMO and LUMO energy levels rise, but the HOMO level increases more significantly than the LUMO level, resulting in a reduced HOMO–LUMO energy gap. This HOMO elevation is primarily attributed to the σ‐donating inductive effect of the electron‐rich tetracoordinate boron moieties. Furthermore, interaction between the π‐orbital of the thienyl moieties and the bonding orbital of the newly formed B–C bond in an out‐of‐phase fashion may also contribute to the HOMO destabilization in **3a·**2PCy_3_ (Figure 5, inset). As a result, **3a·**2PCy_3_ exhibits much red‐shifted calculated absorption wavelength by 57 nm with a slight decrease in oscillator strength, in agreement with the experimentally observed red‐shifted absorption spectra upon conversion from **3a** to **3a·**2PCy_3_.

It should be noted that the phosphine‐added borate moiety serves as a strong σ‐donating group and its electron‐donating effect is stronger even compared to common electron‐donating groups such as diphenylamino (Ph_2_N)‐substituted thiophene moiety. TD‐DFT calculations (Figure S16) revealed that **3a·**2PCy_3_ has a higher‐lying HOMO than that of Ph_2_N‐substituted congener **11**, demonstrating another unique feature of the alkenyl‐strapped diarylboryl group with phosphine.

**Figure 6:**
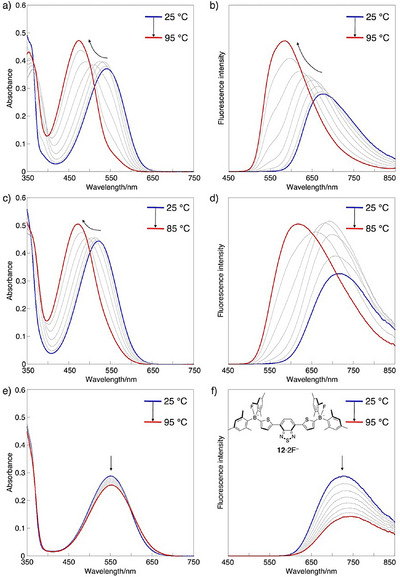
a) UV–vis absorption and b) fluorescence (*λ*_ex_ = 365 nm) spectral changes of **3a** in the presence of an excess amount of PCy_3_ in toluene, with increasing temperature from 25 °C to 95 °C: [**3a**]_0_ = 1.91 × 10^−5^ M and [PCy_3_]_0_ = 1.09 × 10^−2^ M. c) UV–vis absorption and d) fluorescence (*λ*_ex_ = 365 nm) spectral changes of **3a** in the presence of an excess amount of PPh_3_ in CH_3_CN, with increasing temperature from 25 °C to 85 °C: [**3a**]_0_ = 2.20 × 10^−5^ M, [PPh_3_]_0_ = 6.59 × 10^−2^ M. e) UV–vis absorption and f) fluorescence (*λ*_ex_ = 365 nm) spectral changes of **12** in the presence of an excess amount of TBAF in toluene (including 0.1%THF), with increasing temperature from 25 °C to 95 °C: [**12**]_0_ = 1.92 × 10^−5^ M and [TBAF·3H_2_O]_0_ = 1.00 × 10^−4^ M.

A key advantage of the alkenyl‐strapped diarylboryl‐substituted π‐systems lies in their ability to undergo reversible dissociation and association of Lewis bases from their FLP‐type adducts. Notably, the use of bulky phosphines as Lewis bases induces a large entropy gain upon dissociation, thereby facilitating the equilibrium shift toward dissociation at elevated temperatures. To demonstrate this feature, we investigated the temperature‐dependent photophysical properties of the phosphine adducts **3a·**2PR_3_ (Figures 6a–d and S8–S10). Upon gradual heating of a toluene solution of in situ‐generated **3a·**2PCy_3_ in the presence of excess PCy_3_, the absorption spectrum exhibited a progressive blue shift, eventually resembling that of uncoordinated **3a**. This observation indicates dissociation of PCy_3_ from the alkenyl‐strapped diarylboryl units (Figure 6a). The absence of the isosbestic points suggests that the two phosphines dissociate in a random rather than stepwise manner. Correspondingly, the emission maximum shifted significantly from 675 nm at 25 °C to 585 nm at 95 °C (Figure 6b). Importantly, upon cooling, both absorption and emission spectra reverted to their original profiles, which are characteristic of **3a·**2PCy_3_, confirming the re‐association of PCy_3_. This dissociation–association process was fully reversible, as evidenced by the reproducibility of the spectral changes over multiple heating–cooling cycles (Figure S8).

In polar acetonitrile, however, **3a·**2PCy_3_ exhibited only slight blue shifts in the absorption and fluorescence spectra, indicating that the dissociation was suppressed (Figure S9). This behavior is likely attributable to significant stabilization of the zwitterionic phosphonium‐borate structure in polar solvents. To circumvent this, the less Lewis basic triphenylphosphine (PPh_3_) was employed. Upon addition of excess PPh_3_ to an acetonitrile solution of **3a** (2.20 × 10^−5^ M), formation of **3a·**2PPh_3_ was confirmed, displaying an emission maximum at 714 nm in the NIR region with a quantum yield of 0.24. Gradual heating of this solution induced pronounced shifts in the emission spectra spanning the NIR to visible regions, with the emission maximum shifting from 714 nm at 25 °C to 617 nm at 85 °C (Figure 6c,d). Moreover, this solution showed reversible spectral changes over multiple heating–cooling cycles (Figure S10). Thus, modulation of Lewis basicity of phosphines enables temperature‐responsive emission across a wide range of solvent polarity.

Notably, conventional dimesitylboryl‐substituted π‐systems in combination with a fluoride ion do not readily exhibit temperature responsiveness. To confirm this, we prepared bis(dimesitylboryl)‐substituted 4,7‐di(2‐thienyl)‐2,1,3‐benzothiadiazole **12** ^[^ ^88^ ^]^ and examined its fluoride complex **12**·2F^–^ in toluene. The in situ‐generated **12**·2F^–^ solution showed only decreases in absorption and fluorescence intensities upon heating from 25 °C to 95 °C, with negligible wavelength shifts (Figure 6e,f). This result indicates that the fluoride adduct remains intact over this temperature range. Given that sterically protected dimesityboryl groups generally coordinate only with a limited set of small anionic Lewis bases such as fluoride and cyanide ions, achieving reversibility through Lewis base tuning is inherently difficult. By contrast, the mono‐alkenyl‐strapped diarylboryl unit developed in this study uniquely enables a reversible, temperature‐responsive NIR‐to‐red emission change, which is a hallmark of its π‐umpolung reactivity. This unprecedented property arises from the transformation of a boryl‐substituted A–A–A system into a borate‐substituted D–A–D system upon addition of bulky neutral phosphines, highlighting the distinctive potential of this design for temperature‐responsive sensing materials.

## Conclusion

In summary, we have developed a novel class of fluorescent π‐conjugated systems that exhibit reversible, temperature‐responsive red‐to‐NIR fluorescence changes, enabled by reversible π‐umpolung induced through FLP‐type addition of phosphines at terminal boryl groups. Central to this system is a newly designed mono‐alkenyl‐strapped diarylboryl unit, in which two aryl substituents on boron are bridged by an alkenyl chain. A concise and modular synthetic route was established to introduce this unit into a series of π‐conjugated frameworks. Detailed studies on a model compound bearing this boryl group and an electron‐donating triphenylamine moiety demonstrated the susceptibility of the boryl site to FLP‐type addition with bulky phosphines. Of particular note is the incorporation of this boryl unit at both termini of an electron‐accepting benzothiadiazole‐based π‐framework, forming a symmetric A–A–A architecture. Upon coordination with PCy_3_, the system undergoes π‐umpolung, resulting in pronounced red shifts in both absorption and emission spectra, with emission maxima reaching 732 nm in acetonitrile. TD‐DFT calculations demonstrated that the resulting tetracoordinate boron moiety functions as a strong electron‐donating group compared to typical diphenylamino donor. Through appropriate adjustment of the solvent polarity and Lewis bases, these photophysical changes can be made fully reversible under thermal cycling. These findings not only establish a promising π‐system for reversible, thermally responsive photofunctionality in the red–NIR region, but also present a general design concept that exploits the reversible π‐umpolung behavior of boron‐based π‐electron systems. Further efforts in our laboratory are directed toward extending this strategy to molecular systems operable in aqueous media, with the aim of enabling biologically relevant applications, such as intracellular thermometry.

## Supporting Information

Full experimental details can be found in the Supporting Information.

## Conflict of Interests

The authors declare no conflict of interest.

## Supporting information



Supporting Information

Supporting Information

## Data Availability

The data that support the findings of this study are available in the Supporting Information of this article.
