# Supplementary material for: Temperature‐Responsive Near‐Infrared Emission Enabled by Reversible π‐Umpolung with an Alkenyl‐Strapped Diarylboryl Unit
Source: Angew Chem Int Ed Engl. 2026 Jan 24;65(10):e23338. doi: 10.1002/anie.202523338 (PMC12955520; doi:10.1002/anie.202523338)
Supplement: Supplementary file 1 — Supporting Information [file ANIE-65-e23338-s002.pdf]

Supporting Information

Contents

1.	Experimental Details	S2
2.	X-ray Crystallographic Analysis	S6
3.	Photophysical Properties	S7
4.	Temperature-dependent UV–vis–NIR Absorption and Emission Spectra	S10
5.	NMR Studies on Lewis Base Adducts	S13
6.	Theoretical Calculations	S16
7.	References	S26
8.	NMR Spectra	S27

1. Experimental Details

General Procedures. Melting points (mp) were determined with a Yanaco MP-S3 or J3 instrument. ^{1}H, $^{13}C\{^{1}H\}$, ^{11}B, ^{19}F, ^{31}P NMR spectra were recorded on a JEOL ECS-400 spectrometer, a JEOL ECZ-400S spectrometer, a JEOL ECA-600 spectrometer or a JEOL ECA-600 II spectrometer equipped with an UltaCOOL probe in $CDCl_3$, acotone-d_6, dicloromethane-d_2 (400 MHz for ^{1}H, 100 MHz or 150 MHz for $^{13}C\{^{1}H\}$, 128 MHz for ^{11}B, 376 MHz for ^{19}F, and 162 MHz for ^{31}P). The chemical shifts in ^{1}H NMR spectra are reported in δ ppm using the residual proton of the solvents, δ 7.26 in $CDCl_3$, δ 2.05 in acetone-d_6, δ 5.32 in dicloromethane-d_2 as internal standards. The chemical shifts in $^{13}C\{^{1}H\}$ NMR spectra are reported using the solvent signals of $CDCl_3$ (δ 77.16) as an internal standard. The chemical shifts in ^{11}B NMR, ^{19}F NMR and ^{31}P NMR spectra are reported using $BF_3 \cdot OEt_2$ (δ 0.00), CF_3COOH (δ –78.50), and H_3PO_4 (δ 0.00) as an external standard, respectively. Mass spectra were measured with a Bruker micrOTOF Focus spectrometry system with atmospheric pressure chemical ionization (APCI) or a JEOL JMS-T100GCV system with electron ionization (EI). Thin layer chromatography (TLC) was performed on glass plates coated with silica gel $60F_{254}$ (Merck). Column chromatography was performed using PSQ60B (Fuji Silysia Chemical). Florisil (Kanto) and Sephadex® LH-20 (Cytiva) were used in the purification process. Preparative gel permeation chromatography (GPC) was performed using LaboACE LC-5060 Plus equipped with polystyrene gel columns (JAIGEL-2HR Plus, Japan Analytical Industry) using chloroform as eluent. Anhydrous THF and CH_2Cl_2 were purchased from Kanto Chemical and further purified by Glass Contour Solvent Systems, and DMF and 1,4-dioxane were purchased from Wako Chemical. 2-Bromo-3-iodotoluene was prepared according to the literature method.[S1] *N*-$Bu_4NF \cdot 3H_2O$ (TBAF) was purchased from Wako Chemical. All reactions were performed under a nitrogen atmosphere. The microwave-assisted reaction was performed using a Biotage® Initiator+.

3-Allyl-2-bromotoluene (**4**). A mixture of 2-bromo-3-iodotoluene (8.00 g, 26.9 mmol), allytributylstannane (9.80 g, 29.6 mmol) and $Pd(PPh_3)_4$ (853 mg, 0.738 mmol) in DMF (30 mL) was stirred at 90 °C for 17 h. After cooling to room temperature, a 20% aqueous solution of KF (100 mL) was added to the reaction mixture followed by stirring for 5 h. To the reaction mixture, Et_2O (100 mL) was added and filtered off the precipitates. The organic layer of filtrate was separated and washed with H_2O and brine, and dried over Na_2SO_4, filtered, and concentrated under a reduced pressure. The mixture was subjected to silica gel column chromatography (hexane, R_f = 0.77) to give 4.51 g (21.4 mmol) of **4** in 79% yield as a colorless oil: ^{1}H NMR (400 MHz, $CDCl_3$) δ 7.05–7.16 (m, 3H), 5.98 (ddt, J = 16.8, 10.0, 6.4 Hz, 1H), 5.05–5.12 (m, 2H), 3.54 (d, J = 6.4 Hz, 2H), 2.43 (s, 3H); $^{13}C\{^{1}H\}$ NMR (150 MHz, $CDCl_3$) δ 140.0, 138.8, 136.0, 128.9, 127.9, 127.3, 127.0, 116.5, 41.1, 24.2; HRMS (EI) *m/z* calcd for $C_{10}H_{11}{}^{79}Br$ $[M]^{\bullet+}$: 210.0039, found: 210.0042.

Bis(2-allyl-6-methylphenyl)(2-thienyl)borane (**5**). To magnesium turnings (418 mg, 17.2 mmol), a few drops of a concentrated solution of 3-allyl-2-bromotoluene (**4**) in THF were added initially, followed by the dropwise addition of a diluted solution of **4** (total 2.99 g, 14.2 mmol) in THF (total 48 mL) over 1 h at 0 °C. After stirring at 0 °C for 2 h, the reaction mixture was slowly concentrated by vacuum to approximately one-

sixth of its original volume. To a solution of potassium thiophene-2-trifluoroborate (1.36 g, 7.16 mmol) in THF (10 mL), the THF solution of ArMgBr was added at 0 °C. The mixture was gradually warmed up to room temperature and stirred for 17 h. The mixture was passed through a pad of silica gel using a CH_2Cl_2 as eluent and concentrated. The mixture was adsorbed on florisil subsequently subjected to silica gel column chromatography (9:1 hexane/CH_2Cl_2, R_f = 0.50) to give 1.24 g (3.48 mmol) of **5** in 49% yield as a colorless oil: ^{1}H NMR (400 MHz, acetone-d_6) δ 8.20 (dd, J = 4.0, 1.2 Hz, 1H), 7.45 (dd, J = 4.0, 1.2 Hz, 1H), 7.35 (t, J = 4.0 Hz, 1H), 7.27 (t, J = 7.6 Hz, 2H), 7.07–7.09 (m, 4H), 5.70 (ddt, J = 16.8, 10.4, 6.4 Hz, 2H), 4.80–4.88 (m, 4H), 3.23 (d, J = 6.4 Hz, 4H), 2.13 (s, 6H); ^{13}C{^{1}H} NMR (100 MHz, $CDCl_3$) δ 150.3, 144.4, 143.0, 141.2, 141.0, 138.9, 138.3, 129.4, 129.2, 128.1, 126.8, 115.6, 40.9, 24.0; ^{11}B NMR (128 MHz, $CDCl_3$) δ 65.8; HRMS (EI) *m/z* calcd for $C_{24}H_{15}{}^{11}BS$ $[M]^{\bullet+}$: 356.1765, found: 356.1775.

Mono-alkenyl-strapped diphenylthienylborane 1. A mixture of **5** (100 mg, 0.281 mmol), the 2nd generation Grubbs catalyst (11.8 mg, 0.0139 mmol) in CH_2Cl_2 (28 mL) was stirred at room temperature for 3 h under dark conditions. The reaction mixture was adsorbed on florisil and subsequently subjected to silica gel column chromatography (9:1 hexane/CH_2Cl_2, R_f = 0.43) to give 68.4 mg (0.208 mmol) of **1** in 74% yield as a colorless solid: Mp. 127.1–127.9 °C; ^{1}H NMR (400 MHz, $CDCl_3$) δ 7.80 (d, J = 4.8 Hz, 1H), 7.40 (d, J = 3.2 Hz, 1H), 7.12–7.18 (m, 3H), 6.95–6.99 (m, 4H), 5.42–5.50 (m, 2H), 3.71 (dd, J = 16.8, 5.6 Hz, 2H), 3.14 (dd, J = 16.8, 4.0 Hz, 2H), 2.18 (s, 6H); ^{13}C{^{1}H} NMR (150 MHz, $CDCl_3$) δ 147.7, 146.3, 142.3, 139.5, 139.0, 136.0, 135.7, 128.7, 128.2, 125.6, 35.2, 23.5, one signal was not observed due to overlap with other signal; ^{11}B NMR (128 MHz, $CDCl_3$) δ 61.7; HRMS (EI) *m/z* calcd for $C_{22}H_{21}{}^{11}BS$ $[M]^{\bullet+}$: 328.1452, found: 328.1455.

Bis(2-allyl-6-methylphenyl)(5-bromo-2-thienyl)borane (6). To a solution of **5** (200 mg, 0.561 mmol) in THF (3 mL) was added *n*-BuLi (1.6 M in hexane, 0.42 mL, 0.67 mmol) dropwise at –78 °C. After stirring at the same temperature for 1 h, 1,2-dibromo-1,1,2,2-tetrachloroethane (217 mg, 0.666 mmol) was added. The mixture was gradually warmed up to room temperature and stirred for 12 h. A saturated NH_4Cl aqueous solution (15 mL) was added and the mixture was extracted with EtOAc twice. The combined organic layer was washed with brine, dried over Na_2SO_4, filtered, and concentrated under reduced pressure. The mixture was adsorbed on florisil and subsequently subjected to silica gel column chromatography (19:1 hexane/CH_2Cl_2, R_f = 0.62) to give 204 mg (0.469 mmol) of **6** in 83% yield as a colorless oil: ^{1}H NMR (400 MHz, dicloromethane-d_2) δ 7.17–7.27 (m, 4H), 7.06 (m, 4H), 5.67–5.73 (m, 2H), 4.80–4.91 (m, 4H), 3.21 (brs, 4H), 2.15 (s, 6H); ^{13}C{^{1}H} NMR (150 MHz, $CDCl_3$) δ 152.7, 143.6, 143.0, 141.3, 141.1, 138.1, 132.7, 129.5, 128.2, 127.3, 126.9, 115.8, 40.9, 24.0; ^{11}B NMR (128 MHz, $CDCl_3$) δ 65.2; HRMS (APCI) *m/z* calcd for $C_{24}H_{25}{}^{11}B^{79}BrS$ $[M+H]^+$: 435.0948, found: 435.0934.

Compound 8. To a solution of (4-bromophenyl)diphenylamine (149 mg, 0.460 mmol) in THF (3 mL) was added *n*-BuLi (1.6 M in hexane, 0.31 mL, 0.50 mmol) dropwise at –78 °C. After stirring at the same temperature for 2.5 h, tributyltin chloride (159 mg, 0.488 mmol) was added followed by stirring at the same temperature for 1 h, and then at room temperature for 3.5 h. After removing volatiles under reduced pressure,

6 (100 mg, 0.230 mmol), $Pd(PPh_3)_4$ (13.4 mg, 0.0116 mmol) and 1,4-dioxane (1.5 mL) were added followed by stirring under reflux for 17 h. After cooling to room temperature, the mixture was passed through a pad of silica gel/K_2CO_3 using CH_2Cl_2 as eluent. The mixture was adsorbed on florisil and subsequently subjected to silica gel column chromatography (9:1 hexane/CH_2Cl_2, $R_f = 0.19$) to give 116 mg (0.193 mmol) of **8** in 84% yield as a yellow solid: Mp. 78.9–79.9 °C; ^{1}H NMR (400 MHz, dicloromethane-d_2) δ 7.54 (dd, J = 8.4, 1.2 Hz, 2H), 7.37–7.42 (m, 2H), 7.22–7.31 (m, 6H), 7.01–7.11 (m, 12H), 5.73 (m, 2H), 4.83–4.91 (m, 4H), 3.26 (brs, 4H), 2.19 (s, 6H); ^{13}C{^{1}H} NMR (150 MHz, $CDCl_3$) δ 157.9, 148.7, 148.5, 147.3, 144.3, 143.1, 142.7, 141.1, 138.3, 129.5, 129.1, 128.0, 127.7, 127.2, 126.7, 124.9, 124.6, 123.6, 123.1, 115.6, 41.0, 24.1; ^{11}B NMR (128 MHz, $CDCl_3$) δ 64.1; HRMS (APCI) m/z calcd for $C_{42}H_{38}{}^{11}BNS$ $[M]^{\bullet +}$: 599.2813, found: 599.2800.

Compound 2. A mixture of **8** (59.9 mg, 0.0999 mmol), the 2nd generation Grubbs catalyst (4.36 mg, 5.14 μmol) in CH_2Cl_2 (10 mL) was stirred at room temperature for 5 h under dark conditions. The reaction mixture was passed through a pad of silica gel using CH_2Cl_2 as eluent, and then further purified by preparative GPC ($CHCl_3$) to give 46.4 mg (0.0812 mmol) of **2** in 81% yield as a yellow solid: Mp. 108.1–109.1 °C; ^{1}H NMR (400 MHz, dicloromethane-d_2) δ 7.53 (d, J = 8.4 Hz, 2H), 7.34–7.36 (m, 2H), 7.28 (t, J = 8.0 Hz, 4H), 6.97–7.16 (m, 14H), 5.47–5.49 (m, 2H), 3.69 (dd, J = 16.8, 6.0 Hz, 2H), 3.17 (dd, J = 16.8, 4.0 Hz, 2H), 2.22 (s, 6H); ^{13}C{^{1}H} NMR (150 MHz, $CDCl_3$) δ 155.0, 148.1, 147.5, 146.3, 146.2, 142.4, 140.5, 139.6, 135.2, 129.5, 128.2, 127.0, 125.6, 124.8, 124.0, 123.5, 123.4, 35.4, 23.6, two signals were not observed due to overlap with other signals; ^{11}B NMR (128 MHz, $CDCl_3$) δ 61.9; HRMS (APCI) m/z calcd for $C_{40}H_{34}{}^{11}BNS$ $[M]^{\bullet +}$: 571.2500, found: 571.2476.

Compound 9a. To a solution of **5** (504 mg, 1.41 mmol) in THF (5 mL) was added n-BuLi (2.3 M in cyclohexane, 0.73 mL, 1.7 mmol) dropwise at –78 °C. After stirring at the same temperature for 2 h, tributyltin chloride (563 mg, 1.73 mmol) was added, and the mixture was stirred at the same temperature for 30 min and then at room temperature for 17 h. The resulting mixture was concentrated and transferred to a microwave vial. To the mixture, 4,7-dibromo-2,1,3-benzothiadiazole (204 mg, 0.694 mmol), $Pd(PPh_3)_4$ (163 mg, 0.141 mmol) and 1,4-dioxane (1.5 mL) were added, and the mixture was stirred in a microwave synthesizer at 150 °C for 2.5 h. After cooling to room temperature, the mixture was passed through a pad of silica gel/K_2CO_3 using CH_2Cl_2 as eluent. The mixture was adsorbed on florisil and subsequently subjected to silica gel column chromatography (9:1 hexane/EtOAc, $R_f = 0.58$), and then further purified by preparative GPC ($CHCl_3$) to give 78.2 mg (0.0926 mmol) of **9a** in 13% yield as an orange solid: Mp. 98.2–98.8 °C; ^{1}H NMR (400 MHz, dicloromethane-d_2) δ 8.31 (d, J = 4.0 Hz, 2H), 8.02 (s, 2H), 7.51 (d, J = 4.0 Hz, 2H), 7.29 (t, J = 7.6 Hz, 4H), 7.08–7.12 (m, 8H), 5.74 (ddt, J = 16.8, 10.0, 6.4 Hz, 4H), 4.83–4.91 (m, 8H), 3.28 (brs, 8H), 2.21 (s, 12H); ^{13}C{^{1}H} NMR (150 MHz, $CDCl_3$) δ 152.7, 152.0, 151.4, 144.3, 143.1, 142.0, 141.2, 138.2, 130.2, 129.4, 128.2, 127.0, 126.9, 126.7, 115.7, 41.1, 24.1; ^{11}B NMR (128 MHz, $CDCl_3$) δ 65.8; HRMS (APCI) m/z calcd for $C_{54}H_{50}{}^{11}B_2N_2S_3$ $[M]^{\bullet +}$: 844.3317, found: 844.3321.

Compound 3a. A mixture of **9a** (49.4 mg, 0.0585 mmol), the 2nd generation Grubbs catalyst (20.2 mg, 0.0238 mmol) in CH_2Cl_2 (6 mL) was stirred at room temperature for 4 h under dark conditions. The reaction mixture was passed through a pad of silica gel using CH_2Cl_2 as eluent, and then further purified by preparative GPC ($CHCl_3$) to give 25.7 mg (0.0326 mmol) of **3a** in 56% yield as an orange solid: Mp. 155.3–156.3 °C; ^{1}H NMR (400 MHz, dicloromethane-d_2) δ 8.27–8.28 (m, 2H), 7.97 (s, 2H), 7.48 (d, J = 3.6 Hz, 2H), 7.17 (t, J = 7.6 Hz, 4H), 7.00–7.03 (m, 8H), 5.54–5.61 (m, 4H), 3.74 (dd, J = 15.6, 5.6 Hz, 4H), 3.21 (d, J = 15.6 Hz, 4H), 2.24 (s, 12H); ^{13}C{^{1}H} NMR (150 MHz, $CDCl_3$) δ 152.7, 149.2, 149.1, 146.5, 142.1, 139.8, 139.6, 136.3, 129.8, 128.3, 126.7, 126.6, 125.6, 35.2, 23.7, one signal was not observed due to overlap with other signal; ^{11}B NMR (128 MHz, $CDCl_3$) δ 62.2; HRMS (APCI) *m/z* calcd for $C_{50}H_{42}{}^{11}B_2N_2S_3$ $[M]^{\bullet+}$: 788.2691, found: 788.2696.

Compound 9b. To a solution of **5** (198 mg, 0.556 mmol) in THF (3 mL) was added *n*-BuLi (1.6 M in hexane, 0.42 mL, 0.67 mmol) dropwise at –78 °C. After stirring at the same temperature for 2 h, tributyltin chloride (229 mg, 0.704 mmol) was added, the mixture was stirred at the same temperature for 1 h and then at room temperature for 17 h. After removing volatiles under reduced pressure, 1,4-dibromobenzene (66.2 mg, 0.281 mmol), $Pd(PPh_3)_4$ (65.1 mg, 0.0563 mmol) and 1,4-dioxane (1 mL) were added, and the mixture was stirred under reflux for 18 h. After cooling to room temperature, the mixture was passed through a pad of silica gel/K_2CO_3 using CH_2Cl_2 as eluent. The mixture was adsorbed on florisil and subsequently subjected to silica gel column chromatography (9:1 hexane/CH_2Cl_2, R_f = 0.25), and then further purified by preparative GPC ($CHCl_3$) to give 119 mg (0.151 mmol) of **9b** in 54% yield as a yellow solid: Mp. 65.0–66.0 °C; ^{1}H NMR (400 MHz, acetone-d_6) δ 7.86 (s, 4H), 7.79 (d, J = 4.0 Hz, 2H), 7.46 (d, J = 4.0 Hz, 2H), 7.29 (t, J = 7.6 Hz, 4H), 7.09–7.11 (m, 8H), 5.70–5.77 (m, 4H), 4.83–4.89 (m, 8H), 3.30 (brs, 8H), 2.20 (s, 12H); ^{13}C{^{1}H} NMR (150 MHz, $CDCl_3$) δ 156.8, 150.0, 144.2, 143.1, 142.5, 141.1, 138.2, 134.1, 129.2, 128.1, 126.8, 125.8, 115.7, 41.0, 24.1, one signal was not observed due to overlap with other signal; ^{11}B NMR (128 MHz, $CDCl_3$) δ 66.0; HRMS (APCI) *m/z* calcd for $C_{54}H_{52}{}^{11}B_2S_2$ $[M]^{\bullet+}$: 786.3691, found: 786.3655.

Compound 3b. A mixture of **9b** (66.5 mg, 0.0845 mmol), the 2nd generation Grubbs catalyst (28.8 mg, 0.0339 mmol) in CH_2Cl_2 (8.5 mL) was stirred at room temperature for 23 h under dark conditions. The reaction mixture was passed through a pad of Sephadex® LH-20 using $CHCl_3$ as eluent and then further purified by preparative GPC ($CHCl_3$) to give 22.6 mg (0.0309 mmol) of **3b** in 37% yield as a yellow solid: Mp. 135.4–136.4 °C; ^{1}H NMR (400 MHz, $CDCl_3$) δ 7.64 (s, 4H), 7.43 (d, J = 3.6 Hz, 2H), 7.37 (d, J = 3.6 Hz, 2H), 7.15 (t, J = 7.6 Hz, 4H), 6.97–7.01 (m, 8H), 5.49–5.57 (m, 4H), 3.73 (dd, J = 16.0, 5.6 Hz, 4H), 3.19 (d, J = 16.0 Hz, 4H), 2.24 (s, 12H); ^{13}C{^{1}H} NMR (150 MHz, $CDCl_3$) δ 154.0, 147.7, 146.3, 142.2, 140.2, 139.6, 135.7, 134.0, 128.3, 126.6, 125.6, 125.1, 35.3, 23.6, one signal was not observed due to overlap with other signal; ^{11}B NMR (128 MHz, $CDCl_3$) δ 61.9; HRMS (APCI) *m/z* calcd for $C_{50}H_{44}{}^{11}B_2S_2$ $[M]^{\bullet+}$: 730.3065, found: 730.3056.

2. X-ray Crystallographic Analysis

Structural analysis of 1. Single crystals of **1** suitable for X-ray crystallographic analysis were obtained by slow cooling of a hot ethylacetate solution of **1**. Intensity data were collected at 123 K with a Rigaku AFC-10 instrument equipped with a MicroMax-007 microfocus generator with MoKα radiation (λ = 0.71073 Å), and a HyPix6000 hybrid photon counting detector. Total 53451 reflections were measured at the maximum 2θ angle of 63°, of which 5061 were independent reflections (R_{int} = 0.0441). The structure was solved by direct methods (SHELXT-2018/2, Sheldrick, 2018)[S2] and refined by full-matrix least-squares procedure on F^2 for all reflections (SHELXL-2018/1, Sheldrick, 2018).[S3] All non-hydrogen atoms were refined anisotropically and all hydrogen atoms were placed using AFIX instructions. The crystal data are as follows: $C_{22}H_{21}BS$, FW = 328.26, crystal size 0.13 × 0.13 × 0.05 mm^3, *monoclinic*, $P2_1/c$, a = 12.7401(4) Å, b = 17.1136(6) Å, c = 7.9575(2) Å, β = 90.073(3)°, V = 1734.96(9) Å^3, Z = 4, D = 1.257 g cm^{-3}. The refinement converged to R_1 [I>2σ(I)] = 0.0480, wR_2 (all data) = 0.1138, GOF = 1.070. Crystallographic data have been deposited at the Cambridge Crystallographic Data Centre with the deposition number CCDC 2497396. This data can be obtained free of charge from The Cambridge Crystallographic Data Centre at www.ccdc.cam.ac.uk/data_request/cif. One level B alert in the checkCIF report arises from the disorder of the thiophene ring.

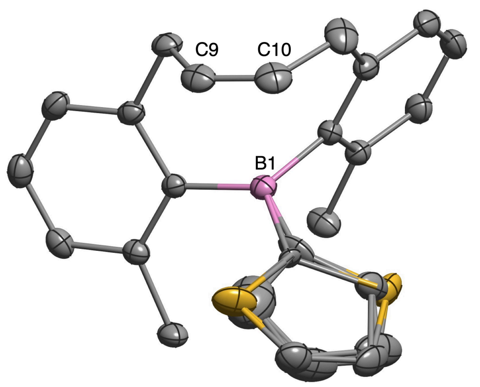

Figure S1. *ORTEP* diagram of **1** (50% probability for thermal ellipsoids). Hydrogen atoms are omitted for clarity. Selected lengths (Å):C9–C10, 1.324(2); B1···C9, 2.655; B1···C10, 2.724.

Structural analysis of 3a. Single crystals of **3a** suitable for X-ray crystallographic analysis were obtained by two layer diffusion of methanol into a CH_2Cl_2 solution of **3a**. Intensity data were collected at 138 K with a Rigaku Single Crystal X-ray diffractometer equipped with FR-X generator, Varimax optics, and PILATUS 200K photon counting detector with MoKα radiation (λ = 0.71073 Å). Total 25397 reflections were measured at the maximum 2θ angle of 60°, of which 6225 were independent reflections (R_{int} = 0.0294). The structure was solved by direct methods (SHELXT-2018/2, Sheldrick, 2018)[S2] and refined by full-matrix least-squares procedure on F^2 for all reflections (SHELXL-2018/1, Sheldrick, 2018).[S3] All non-hydrogen atoms were refined anisotropically and all hydrogen atoms were placed using AFIX instructions. The crystal data are as follows: $C_{52}H_{46}B_2Cl_4N_2S_3$, FW = 958.51, crystal size 0.25 × 0.20 × 0.05 mm^3, *monoclinic*, $C2/c$, a = 34.8259(10) Å, b = 16.4508(5) Å, c = 8.2363(3) Å, β = 94.922(3)°, V = 4701.3(3) Å^3, Z = 4, D = 1.354 g cm$^-$

[3]. The refinement converged to R_1 [I>2σ(I)] = 0.0610, wR_2 (all data) = 0.1826, GOF = 1.026. Crystallographic data have been deposited at the Cambridge Crystallographic Data Centre with the deposition number CCDC 2497397. This data can be obtained free of charge from The Cambridge Crystallographic Data Centre at www.ccdc.cam.ac.uk/data_request/cif.

3. Photophysical Properties

General Methods. UV–vis absorption spectra were measured with a JASCO V-750 spectrophotometer equipped with a JASCO ETCR-762 cell holder set at 25 °C. Fluorescence spectra were measured with a JASCO FP-8500 spectrofluorometer equipped with a JASCO ETC-815 cell holder set at 25 °C. Absolute fluorescence quantum yields were determined with a Hamamatsu Photonics C11347-01 for **2** and **3b** and a Hamamatsu Photonics C13534-02 equipped with NIR measurement unit C13684-01 for **3a**, **3a**·2PCy$_3$ and **3a**·2PPh$_3$ with an integrating sphere system. The excitation light for **3a**·2PCy$_3$ and **3a**·2PPh$_3$ was prepared from a high-power Xenon lamp, passed through A13686-475 and NIR cut filters. Time resolved fluorescence spectra were measured with a Hamamatsu Photonics Quantaurus-Tau C16361-02 for **2**.

3.1. Photophysical Properties of 2

Table S1. Photophysical Properties of **2**, **D**, and **E**

Compd.	Solvent	λ_{abs} /nm[a]	ε /10^4 $M^{-1}cm^{-1}$	λ_{em} /nm[b]	Φ_F	τ_1 /ns	τ_2 /ns
	cyclohexane	397	3.37	440	0.69[c]	1.8	–
	toluene	400	3.22	465	0.73[c]	1.9	–
**2**	$CHCl_3$	399	3.14	488	0.71[c]	2.3	–
	CH_2Cl_2	398	3.16	507	0.74[c]	2.8	–
	CH_3CN	396	3.17	538	0.68[c]	3.4	–
	cyclohexane	388	3.36	431	0.25	0.52	1.7
	toluene	392	3.24	452	0.23	0.54	1.7
**D**[d]	$CHCl_3$	390	2.97	473	0.19	0.52	2.4
	CH_2Cl_2	393	3.29	488	0.21	0.59	2.5
	CH_3CN	–	–	–	–	–	–
	cyclohexane[e]	405	3.65	444	0.94	2.3	–
	toluene[d]	412	3.31	468	0.94	2.2	–
**E**[d,e]	$CHCl_3$[e]	412	3.38	490	0.92	2.6	–
	CH_2Cl_2[e]	412	3.57	512	0.92	3.1	–
	CH_3CN[e]	409	3.72	542	0.88	3.7	–

[a] Only the longest absorption maximum wavelengths are shown. [b] Emission maximum wavelengths upon excitation at λ_{ex} = 360 nm. [c] Absolute fluorescence quantum yields upon excitation at λ_{ex} = 380 nm determined by a calibrated integrating sphere system with an error of ±3%. [d] Data from ref. [S4]. [e] Data from ref. [S5].

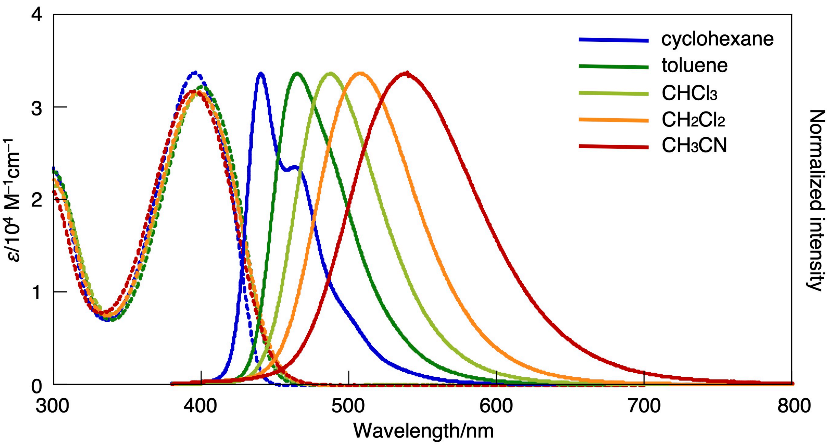

Figure S2. UV–vis absorption (dotted line) and emission (solid line) (λ_{ex} = 360 nm) spectra of **2** in various solvents.

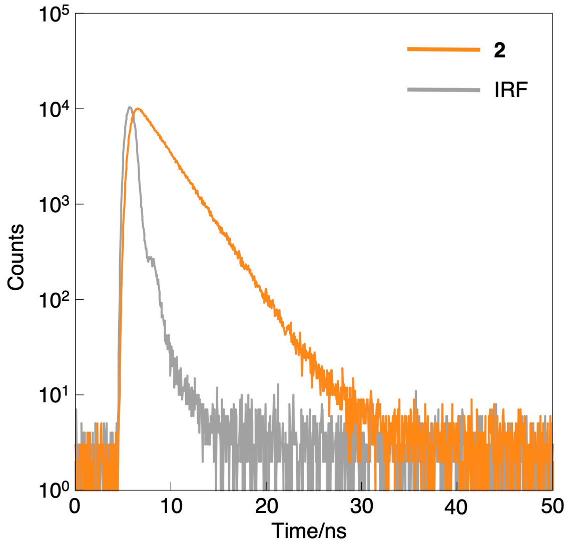

Figure S3. Fluorescence decay of **2** (λ_{em} = 500 nm) with the excitation at 365 nm in CH_2Cl_2: τ = 2.8 ns.

3.2. UV–vis Absorption and Fluorescence Spectral Changes of 2 with PCy_3 Addition

Methods. Dilute sample solutions of **2** using anhydrous toluene were placed under an argon atmosphere in a 1 cm square quartz cuvette equipped with J-Young stopcock at 25 °C. The titration fitting was conducted by considering the absorption of the compounds both prior to and after the addition reaction.

3.3. Photophysical Properties of 3 and their Lewis Base Adducts

Methods. In the measurements involving the addition of Lewis bases, dilute sample solutions of **3** using anhydrous solvents were placed under an argon atmosphere in a 1 cm square quartz cuvette equipped with J-Young stopcock at 25 °C.

Table S2. Photophysical Properties of **3b**

Compd.	Solvent	λ_{abs} /nm	ε /10^4 $M^{-1}cm^{-1}$	λ_{em} /nm[a]	Φ_F[b]
**3b**	cyclohexane	388	5.55	428	0.45
	toluene	392	5.39	435	0.41
	CH_2Cl_2	391	5.59	435	0.38
	CH_3CN	388	–[c]	436	0.36

[a] Only the shortest emission maximum wavelengths upon excitation at λ_{ex} = 350 nm. [b] Absolute fluorescence quantum yields upon excitation at λ_{ex} = 370 nm determined by a calibrated integrating sphere system with an error of ±3%. [c] Not determined due to low solubility.

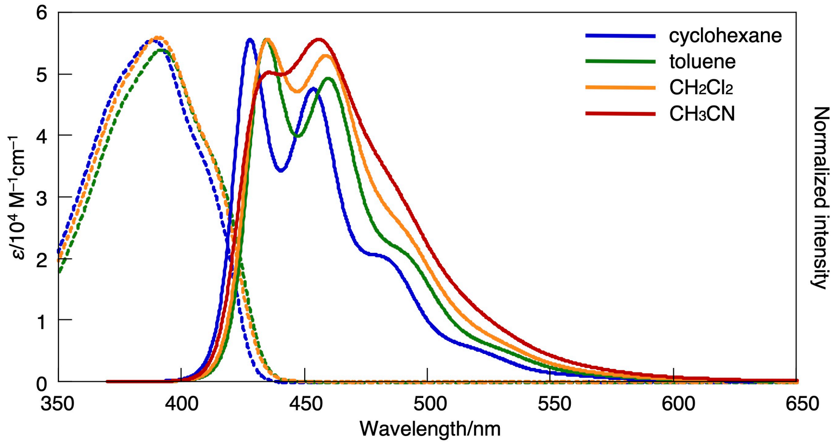

Figure S4. UV–vis absorption (dotted line) and emission (solid line) (λ_{ex} = 350 nm) spectra of **3b** in various solvents.

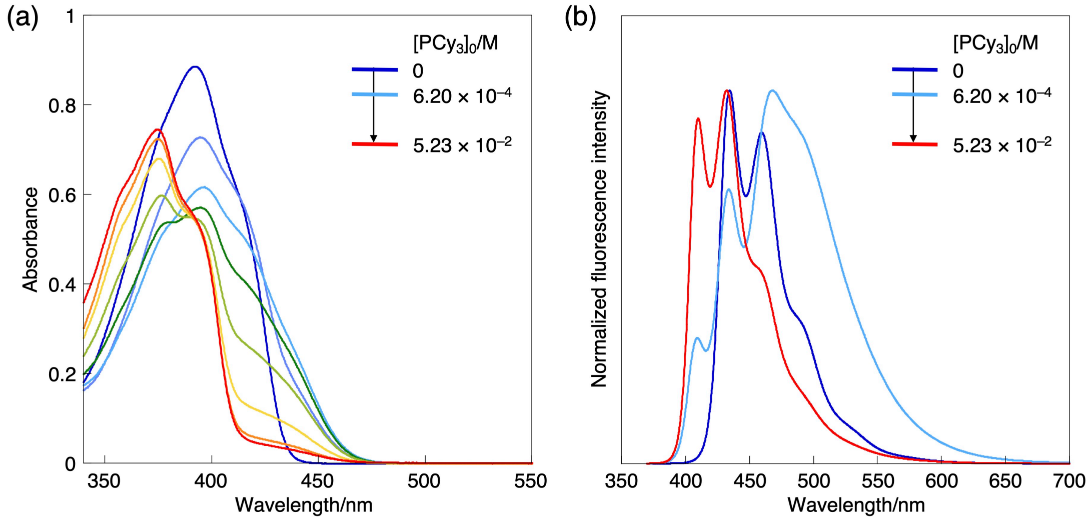

Figure S5. (a) UV–vis absorption spectral changes and (b) representative normalized fluorescence spectra (λ_{ex} = 350 nm) upon addition of PCy_3 to a toluene solution of **3b** (1.64×10^{-5} M) at 25 °C.

3.4. Photostability

Methods. UV–vis absorption spectra were measured with a Shimazu UV-3600 Plus spectrometer equipped with a UNISOKU CoolSpeck USP-203-B cell holder set at 25 °C. Dilute sample solutions of **3a** and *in situ*-generated **3a**·2PCy$_3$ in anhydrous toluene were prepared under an argon atmosphere and placed in a 1 cm square quartz cuvette. Photostability was evaluated by monitoring changes in the absorption spectra as a function of irradiation time. Irradiation was performed using an LED lamp (PFBR-150GR, CCS Inc.) equipped with a band-path filter centered at 515 nm (Edmund Optics, full width at half maximum = 10 nm). The irradiation power density at the output of the glass fiber was 1800 W m^{-2}, as measured using HD2302.0 LightMeter equipped with an LP471RAD probe. The sample solutions exhibited identical absorbance at 515 nm before and after the addition of PCy$_3$.

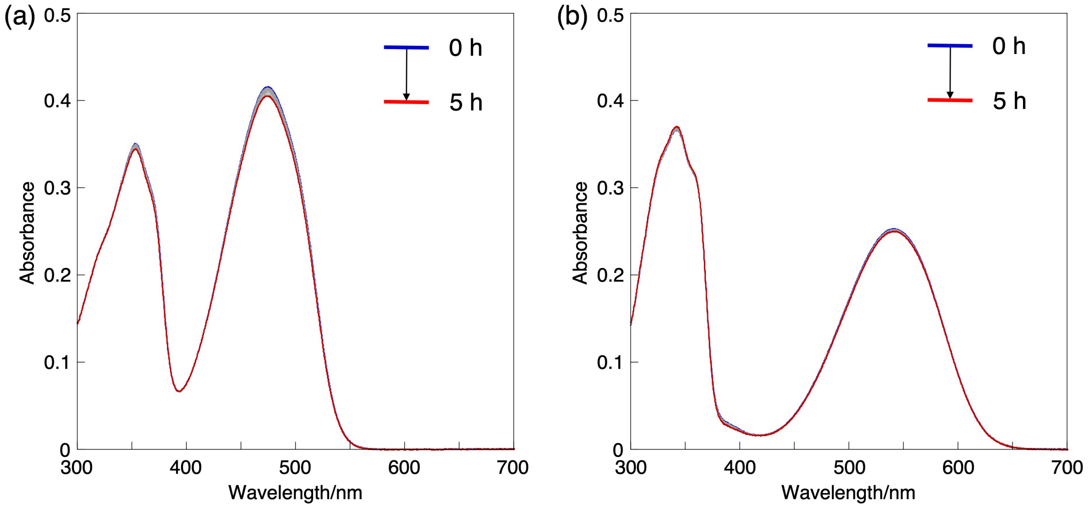

Figure S6. Absorption spectral change of (a) **3a** (1.36×10^{-5} M) and (b) **3a**·2PCy$_3$ ([**3a**]$_0$ = 1.36×10^{-5} M, [PCy$_3$]$_0$ = 7.07×10^{-2} M) upon irradiation with an LED lamp (band-path filter centered at 515 nm, full width at half maximum = 10 nm, 1800 W m^{-2}) at 25 °C in toluene.

4. Temperature-dependent UV–vis–NIR Absorption and Emission Spectra

General methods. UV–vis absorption spectra were measured with a JASCO V-750 spectrophotometer equipped with a JASCO ETCR-762 cell holder. Fluorescence spectra were measured with a JASCO FP-8500 spectrofluorometer equipped with a JASCO ETC-815 cell holder. In all measurements, dilute sample solutions using anhydrous toluene or CH_3CN were prepared under an argon atmosphere and placed in a 1 cm square quartz cuvette equipped with J-Young stopcock. For temperature dependent-absorption and fluorescence measurements conducted from 25 °C to 95 °C (or 85 °C), the temperature was increased at a rate of 5 °C/min, and the spectra were recorded after holding the targeted temperature for 10 s.

4.1. Temperature-dependent UV–vis Absorption Spectral Changes of 2 with and without PCy_3

Method. The thermodynamic parameters ΔH and ΔS of **2** were determined by using the equation of $\Delta G = \Delta H – T\Delta S$ and $\Delta G = –RT\ln K$, where T is the absolute temperature, R is the gas constant, and K is the equilibrium constant. Measurements were carried out on toluene solutions of **2**, both in the presence and absence of PCy_3. Spectral intensities were analyzed by fitting the data under the assumption that the absorbance of **2**·PCy_3 adduct at 410 nm is negligible.

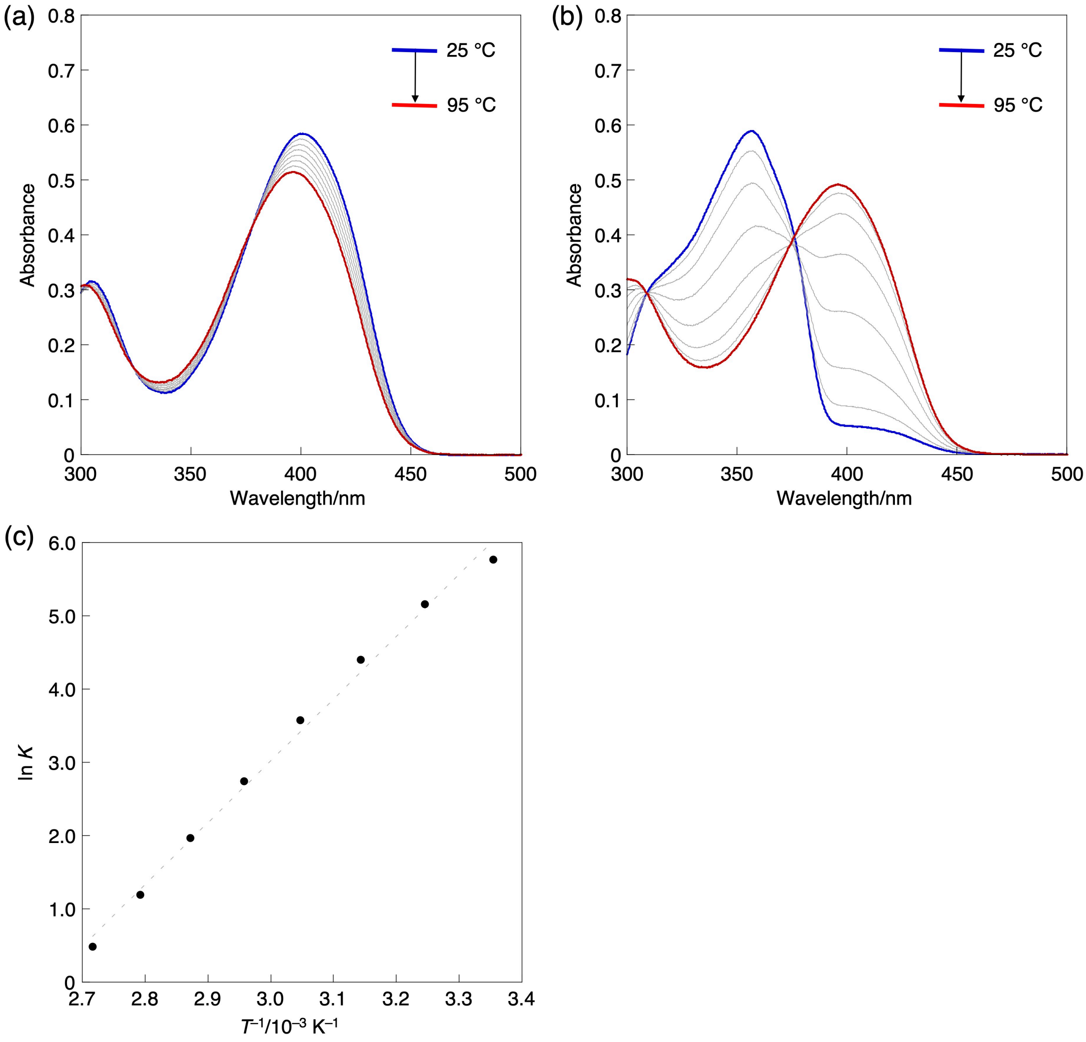

Figure S7. (a) UV–vis absorption spectral changes of (a) **2** and (b) **2** in the presence of an excess amount of PCy_3 in toluene, with increasing temperature from 25 °C to 95 °C: $[\mathbf{2}]_0 = 1.82 \times 10^{-5}$ M and $[PCy_3]_0 = 3.19 \times 10^{-2}$ M. (c) The ln K–($1/T$) plots and the fitting line for the enthalpy change ΔH and the entropy change ΔS in the equilibrium between **2** and **2**·PCy_3. The values of ΔH and ΔS were calculated to be –70.5 kJ mol^{-1} and –186 $JK^{-1}mol^{-1}$, respectively.

4.2. Temperature-dependent UV–vis Absorption and Emission Spectra of 3 with PR_3 addition

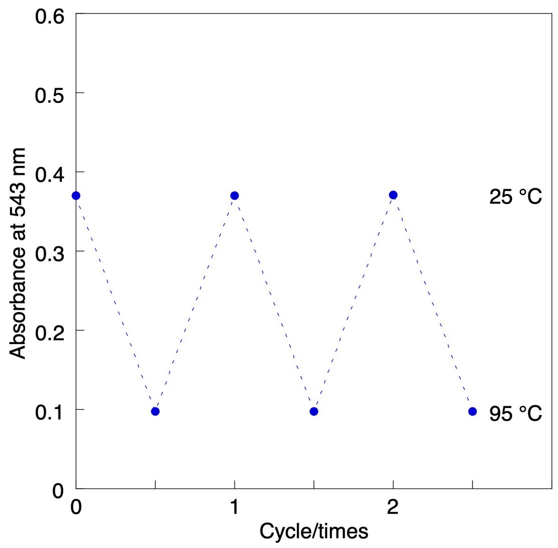

Figure S8. Temperature-dependent variation in the absorbance of **3a** at 543 nm in toluene in the presence of an excess amount of PCy_3, with temperature cycled between 25 °C and 95 °C: $[\mathbf{3a}]_0 = 1.91 \times 10^{-5}$ M, $[PCy_3]_0 = 1.09 \times 10^{-2}$ M.

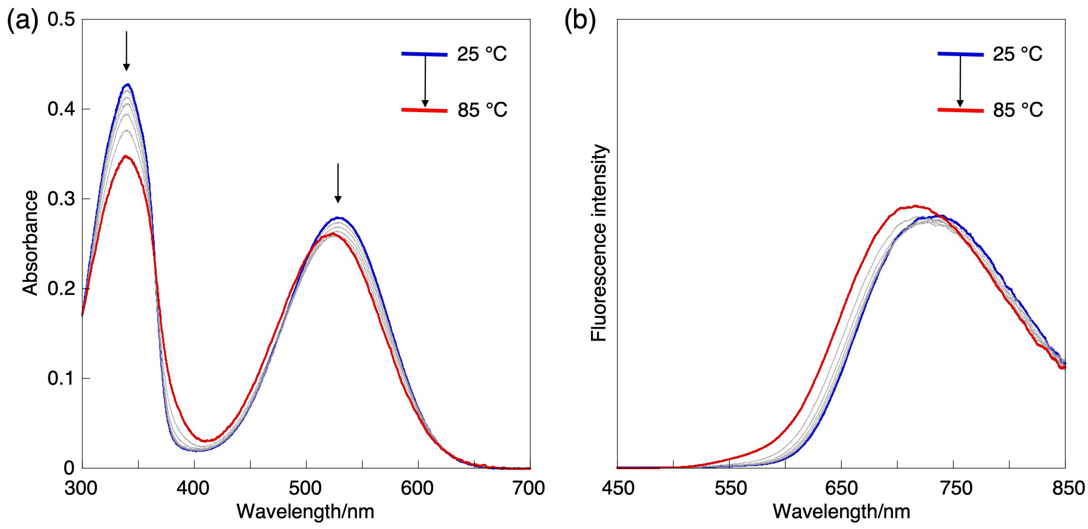

Figure S9. (a) UV–vis absorption and (b) fluorescence (λ_{ex} = 365 nm) spectral changes of **3a** in the presence of an excess amount of PCy_3 in CH_3CN, with increasing temperature from 25 °C to 85 °C: $[\mathbf{3a}]_0 = 1.39 \times 10^{-5}$ M, $[PCy_3]_0 = 4.24 \times 10^{-4}$ M.

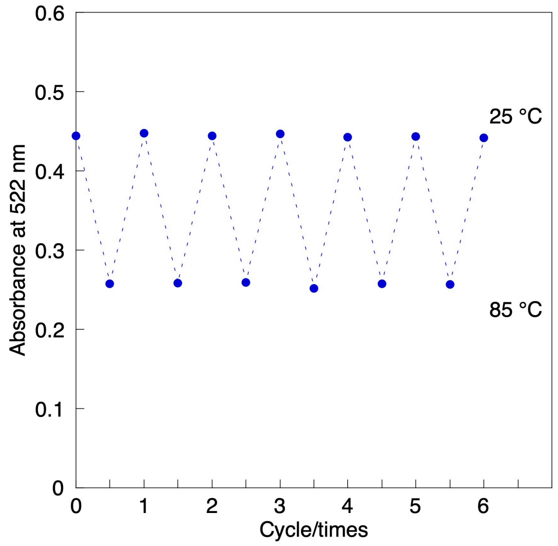

Figure S10. Temperature-dependent variation in the absorbance of **3a** at 522 nm in CH_3CN in the presence of an excess amount of PPh_3, with temperature cycled between 25 °C and 85 °C: $[\mathbf{3a}]_0 = 2.20 \times 10^{-5}$ M, $[PPh_3]_0 = 6.59 \times 10^{-2}$ M.

5. NMR Studies on Lewis Base Adducts

5.1. ^{11}B and ^{31}P NMR Spectra with PCy_3 Addition

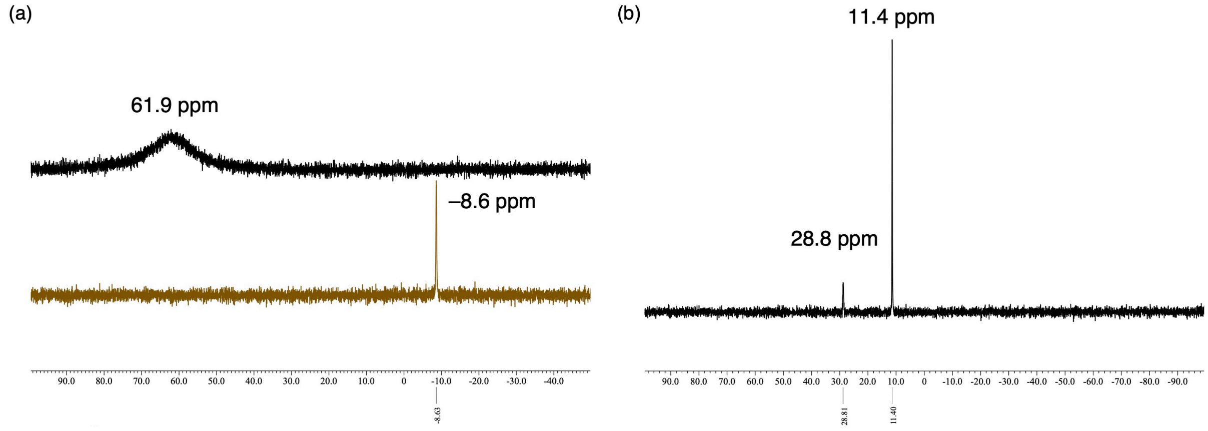

Figure S11. (a) ^{11}B NMR spectra (128 MHz) of **2** in the absence (top) and presence of 10 equiv of PCy_3 (bottom), and (b) ^{31}P NMR spectrum (162 MHz) of **2** in the presence of 10 equiv of PCy_3 in anhydrous and degassed $CDCl_3$.

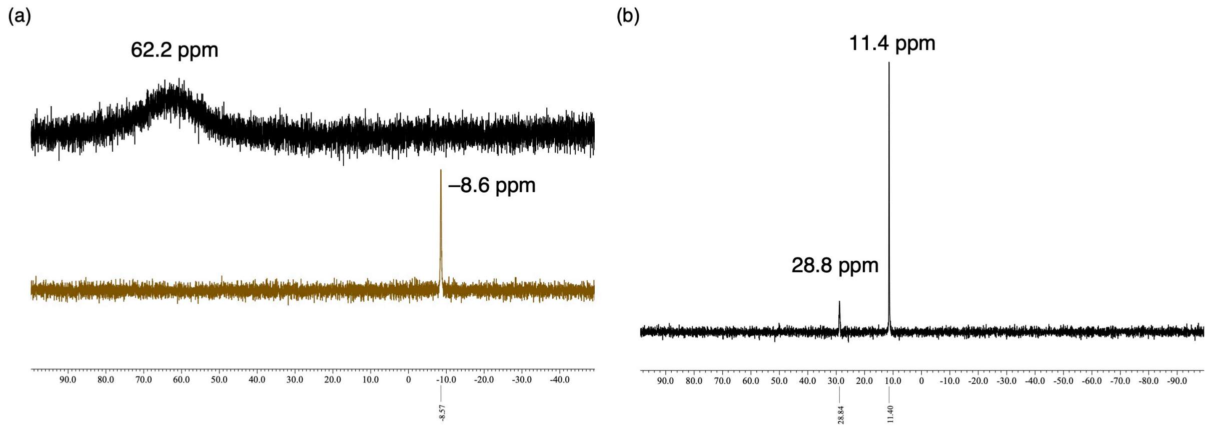

Figure S12. (a) ^{11}B NMR spectra (128 MHz) of **3a** in the absence (top) and presence of 20 equiv of PCy_3 (bottom), and (b) ^{31}P NMR spectrum (162 MHz) of **3a** in the presence of 20 equiv of PCy_3 in anhydrous and degassed $CDCl_3$.

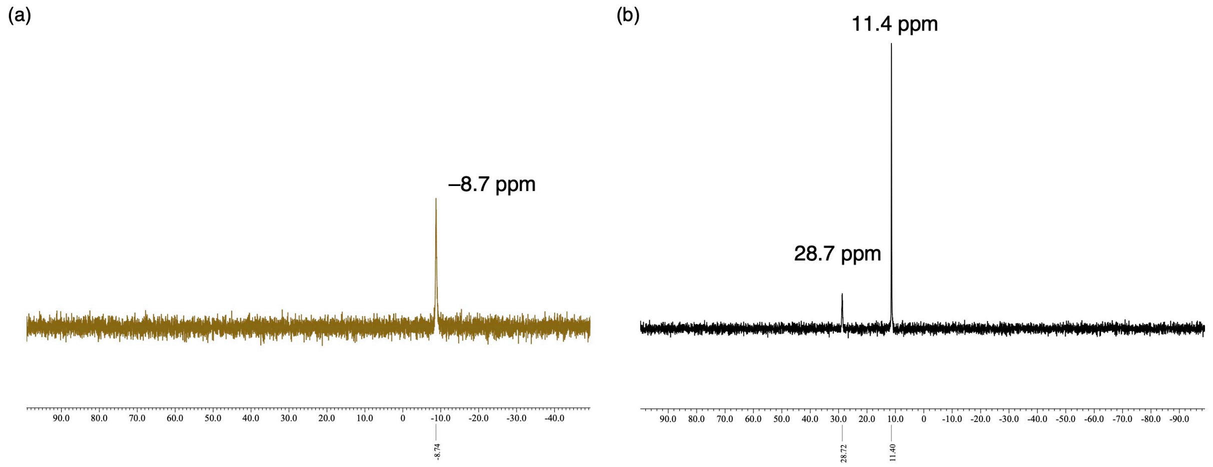

Figure S13. (a) ^{11}B NMR spectrum (128 MHz) of **D** in the presence of 10 equiv of PCy_3, and (b) ^{31}P NMR spectrum (162 MHz) of **D** in the presence of 10 equiv of PCy_3 in anhydrous and degassed $CDCl_3$.

5.2. ^{11}B NMR Spectra with TBAF Addition

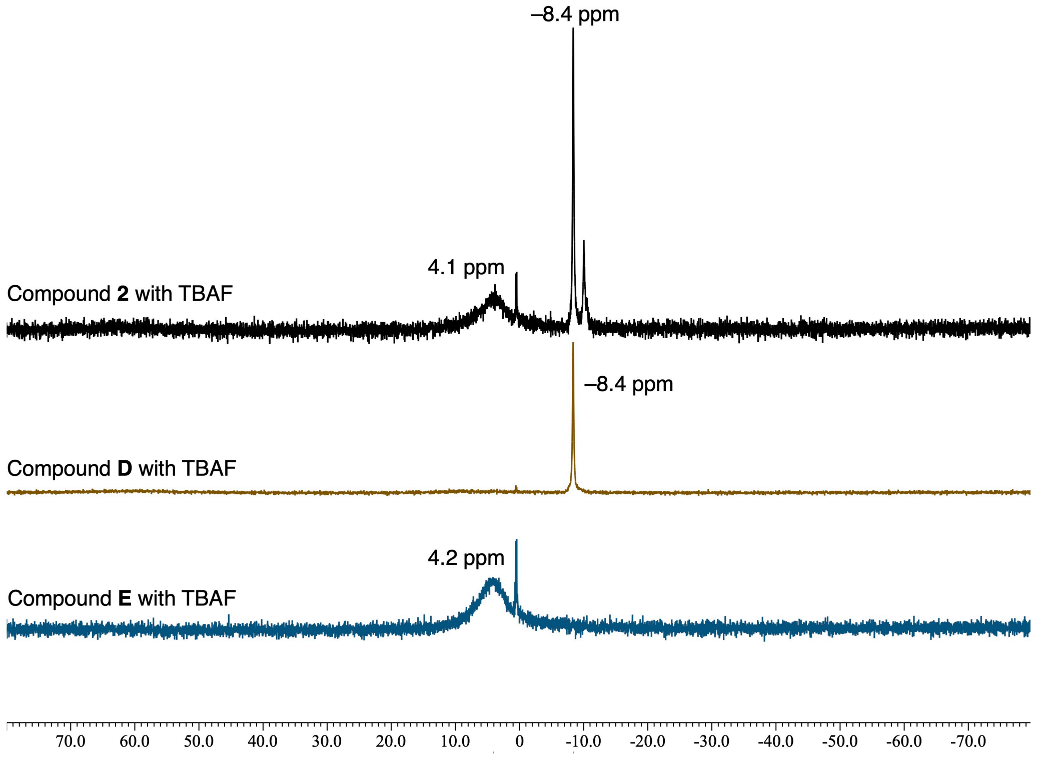

Figure S14. ^{11}B NMR spectra (128 MHz) of **2**, **D**, and **E** in the presence of an excess amount of TBAF in $CDCl_3$ (including 10% THF).

6. Theoretical Calculations

Computational methods. All theoretical calculations were conducted using the Gaussian 16 Revision B.01 suite of programs.[S6] DFT calculations of **2**, **2**·PCy_3, **D**, **D**·PCy_3 and PCy_3 were performed at the GD3-M06-2X/6-311G(d) level of theory including solvent effect of toluene using the polarizable continuum model (PCM).[S7,S8] TD-DFT calculations of **10**, **3a**, **3a**·2PCy_3 and **11** were performed at the M06-2X/6-31G(d) level of theory using the optimized geometries obtained at the same level.

Owing to the flexible linker moieties, these molecules give several local minima with small energy differences upon structural optimization. Referring to the crystal structures of **1**, **3a**, and **D**,[S4] the optimized structures of **2**, **3a** and **D** were adopted in which the olefinic moiety is located on the side opposite to the sulfur atom of the thienyl group. The cartesian coordinates are listed in Tables S4–S12.

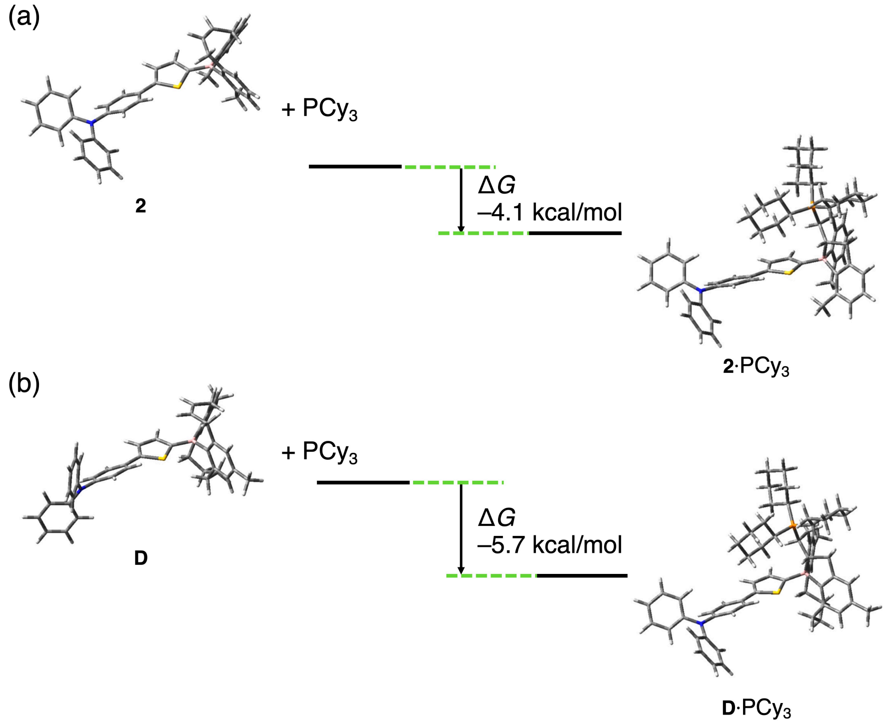

Figure S15. Gibbs free energy differences of PCy_3 addition reaction for (a) **2** and (b) **D** calculated at the GD3-M06-2X/6-311G(d) level of theory in toluene (PCM).

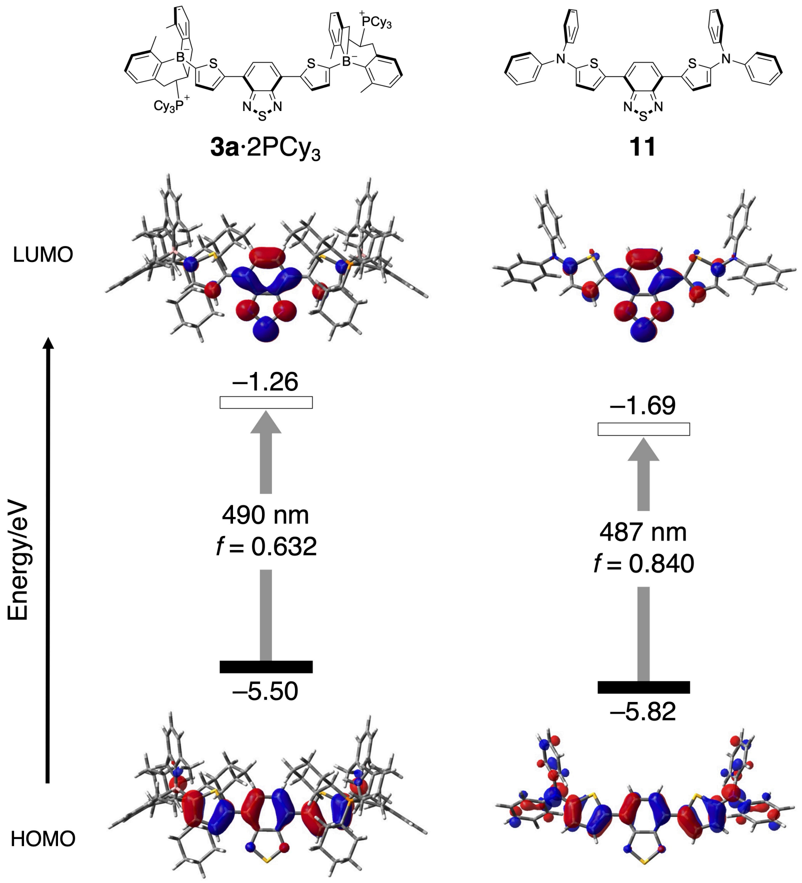

Figure S16. Energy diagrams and Kohn–Sham HOMOs and LUMOs for **3a**·2PCy$_3$ and **11** calculated at the TD-M06-2X/6-31G(d) level of theory.

Table S3. Excited Energies of **10**, **3a**, **3a**·2PCy$_3$ and **11** Using Optimized Structures in S$_0$, Calculated at the TD-M06-2X/6-31G(d) Level of Theory

Compound	Excited state	Transition energy /eV[a]	CI coefficient	f[b]
**10**	1	3.05 (406)	0.702: HOMO → LUMO	0.438
**3a**	1	2.87 (433)	0.695: HOMO → LUMO	1.023
**3a**·2PCy$_3$	1	2.53 (490)	0.696: HOMO → LUMO	0.632
**11**	1	2.55 (487)	–0.133: HOMO–2 → LUMO<br>0.684: HOMO → LUMO	0.840

[a] Corresponding wavelength (nm) in shown in the parentheses. [b] Oscillator strength.

Table S4. Cartesian Coordinates of the Optimized Structure for **2** in Toluene (PCM) at the GD3-M06-2X/6-311G(d) Level of Theory

atom	X	Y	Z	atom	X	Y	Z
S	-1.18250924	0.41424059	-0.13141595	C	-4.41370941	3.46418463	1.37575673
N	5.58365761	-0.04340395	-0.05551432	H	-4.29428021	3.97291214	2.32748358
C	1.39776419	-0.65301250	-0.05299784	C	-4.89818778	1.47837885	-2.39999388
C	-0.04964608	-0.88616295	-0.04769342	H	-4.00089468	0.92272824	-2.68551015
C	-0.70638312	-2.09709460	0.00288853	H	-5.09521985	2.21031325	-3.18422600
H	-0.18800027	-3.04360435	0.09015027	C	8.18526945	-3.27164204	0.70502694
C	-5.22471990	-1.08037427	-0.37720473	H	8.85448420	-4.10109345	0.90072663
C	-4.72622824	2.15448289	-1.06024120	C	7.20996715	1.70606712	0.44897240

C	6.12122851	1.24596507	-0.29640311	H	7.63465232	1.07119393	1.21842702
C	-7.51542196	-2.65725372	-0.82801339	C	-7.42000691	-1.87724530	0.32131928
C	7.63120595	-1.28080783	-0.54414236	H	-8.22410264	-1.88528001	1.05049827
H	7.86418782	-0.56185473	-1.32158583	C	5.57277074	2.06732127	-1.28518272
C	4.18885775	-0.24367453	-0.05636932	H	4.73288056	1.70897278	-1.86978510
C	6.45756362	-1.13054738	0.19906668	C	-6.19440595	-0.29235010	1.83761577
C	-5.33061733	-1.87353548	-1.53516716	H	-6.21541195	0.77958029	1.63715370
C	3.32445373	0.73432857	0.44825167	H	-7.06270342	-0.51812538	2.46280066
H	3.73450611	1.65184836	0.85393752	C	6.15695094	-2.05897321	1.19922423
C	1.95451081	0.53081719	0.44430343	H	5.25075011	-1.93874113	1.78228330
H	1.30889530	1.29464907	0.86509951	C	7.01306643	-3.12571560	1.44093238
C	-5.02101872	3.51188485	-0.94299919	H	6.76716623	-3.83892991	2.21956828
H	-5.37272761	4.05695834	-1.81342956	C	-4.94445961	-0.69676098	2.57010461
C	8.49018757	-2.34105815	-0.28382605	H	-4.93978758	-1.73950089	2.88412114
H	9.39774333	-2.44542457	-0.86766151	C	-3.49645186	1.43977010	2.50455348
C	-6.47808776	-2.64950230	-1.74265862	H	-2.40559056	1.50232145	2.41900135
H	-6.54701802	-3.25272973	-2.64350629	H	-3.74298647	2.04071235	3.38716765
C	-4.87818771	4.16608389	0.27277790	C	7.74231904	2.96504504	0.20153480
C	-6.29907387	-1.08928030	0.54655379	H	8.58750395	3.30900947	0.78700020
C	-2.54951628	-0.65636430	-0.09671407	C	6.10058875	3.33162709	-1.51351094
C	-4.25981163	1.43310538	0.05764567	H	5.66441900	3.95835770	-2.28315199
C	-2.11055302	-1.96139275	-0.03144813	C	-3.83286985	0.00088233	2.80526498
H	-2.80308144	-2.79499287	0.00673158	H	-3.03397012	-0.53399172	3.31446944
C	3.63961794	-1.42734631	-0.56385487	C	7.18963309	3.78682959	-0.77606915
H	4.29545431	-2.18630739	-0.97446669	H	7.60320146	4.77100735	-0.96168990
C	-4.29033899	-1.90642098	-2.63388069	B	-3.99264906	-0.12191421	-0.10087890
H	-3.98054112	-2.93516122	-2.83572869	H	-5.72908436	0.76793558	-2.38319272
H	-3.39642281	-1.33432927	-2.40098452	H	-4.71276161	-1.50925331	-3.56163654
C	2.26960451	-1.62523972	-0.55831233	H	-5.12470327	5.21857556	0.35986661
H	1.86625634	-2.53615810	-0.98660935	H	-8.39429774	-3.26846518	-1.00202531
C	-4.08931840	2.11038422	1.27894878				

Table S5. Cartesian Coordinates of the Optimized Structure for **2**·PCy_3 in Toluene (PCM) at the GD3-M06-2X/6-311G(d) Level of Theory

atom	X	Y	Z	atom	X	Y	Z
S	0.41973086	1.93832658	0.01320192	C	1.08759960	-1.89242858	-0.07864624
P	-3.09179301	-1.77012205	0.43190379	H	1.72286308	-1.54436226	-0.89629438
N	6.92720552	-0.06620668	-0.01370466	H	1.34189728	-1.27038257	0.78824739
C	-0.51026004	1.58477906	-2.32122476	C	7.56969274	-1.05264291	-0.79699095
H	-1.18690257	1.54364575	-3.16859496	C	7.42544839	1.89755732	1.34583964
C	2.91725722	1.01201446	-0.88276670	H	6.77420683	2.48699580	0.71040511
C	3.35674822	0.84778339	0.43708477	C	8.44563090	-0.21723651	1.89523158
H	2.65692332	0.97719242	1.25617971	H	8.58012680	-1.27386988	1.69334388
C	-3.52449898	3.07402397	1.54751708	C	1.36915502	-3.35867647	0.24148076
C	-0.93198598	2.00668248	-1.08612764	H	2.41063522	-3.48340701	0.54878848
C	-3.50899218	2.41528354	-1.77678649	H	1.23158939	-3.96214854	-0.66413992
C	1.52031848	1.33041507	-1.19423530	C	-4.18906167	-4.03619695	-2.87325719
C	0.86271169	1.20060638	-2.38847050	H	-5.11803814	-3.48817161	-3.07062666
H	1.33460083	0.82123448	-3.28747796	H	-3.69174112	-4.17178826	-3.83640497
C	-5.36396719	1.46397876	-3.08008682	C	0.42730876	-3.87140756	1.32993282
H	-6.05167901	0.63766504	-3.24481201	H	0.62634285	-3.34351338	2.27020221
C	-4.42565521	1.37516343	-2.04403998	H	0.59875185	-4.93387232	1.52055516
C	-3.57460448	3.53457710	-2.64592583	C	8.07669021	2.48555505	2.42233434
C	-2.62406422	4.69128916	-2.48670879	H	7.92807936	3.54127284	2.61956542
H	-2.76037600	5.42382952	-3.28456199	C	9.10599558	0.38380943	2.95920203
H	-1.58768982	4.35030075	-2.51039925	H	9.75715559	-0.21524230	3.58599482
C	-2.55329262	3.49985584	0.61533119	C	-4.26873021	-4.38476427	0.06931941
C	-3.90282104	3.83354965	2.64323078	H	-3.33678157	-4.92327635	0.25864979
H	-4.65591482	3.46770878	3.33650004	H	-4.76929684	-4.26030941	1.03128282
C	-2.89671164	1.05343638	0.43370062	B	-2.41595933	2.32320330	-0.53011195

H	-2.06818116	0.82469563	1.11911205	C	8.92401883	1.73604157	3.23304748
C	-3.28187710	5.06336287	2.85700803	H	9.43538625	2.19982370	4.06823526
C	-1.30129484	-2.15895101	0.67450408	C	-4.51349824	-5.39044558	-2.24159283
H	-1.05157097	-1.59987576	1.58805725	H	-3.58821746	-5.97030716	-2.14022277
C	-4.51302025	3.60780646	-3.67314064	H	-5.17431492	-5.96445954	-2.89544138
H	-4.53139423	4.48637727	-4.31165713	C	-5.15343182	-5.21979562	-0.86328062
C	-3.21185812	-0.15657252	-0.44945773	H	-5.34303881	-6.19454935	-0.40738454
H	-2.40131747	-0.23214700	-1.18567472	H	-6.12662628	-4.72635487	-0.97088372
C	-4.04360644	1.68420394	1.24937662	C	8.92489589	-0.93064737	-1.12105982
H	-4.95240145	1.76223518	0.64060500	H	9.48049427	-0.06886727	-0.76909783
H	-4.32556007	1.16604263	2.17315597	C	6.85677991	-2.16205762	-1.26315402
C	4.66522117	0.49307569	0.72638010	H	5.80676758	-2.26290977	-1.01240411
H	4.97735037	0.36054437	1.75634368	C	9.55144942	-1.90517814	-1.88663008
C	5.58992476	0.30018182	-0.30202382	H	10.60255070	-1.79475031	-2.12884126
C	-5.42083766	2.57909352	-3.89407332	C	8.83902465	-3.00493977	-2.35589765
C	-0.73430325	5.23919078	0.06568541	H	9.33001060	-3.75929849	-2.95921571
H	-0.45671990	4.56968475	-0.74239859	C	7.48840778	-3.12286601	-2.04227846
H	0.14454378	5.36599209	0.70460348	H	6.92058991	-3.97615984	-2.39623116
C	-2.28086594	5.47816152	1.99245876	C	-3.41129392	-2.66119042	3.13786407
H	-1.76445864	6.41578238	2.18471835	H	-2.32327620	-2.67332315	3.23646977
C	-1.89514182	4.71170903	0.88084168	H	-3.71965374	-3.66367785	2.82506425
C	-3.97839461	-3.02129011	-0.57922222	C	-5.96999727	-1.18277749	3.39578108
H	-4.94936647	-2.53420681	-0.74624492	H	-5.62830848	-0.19862413	3.73697387
C	3.85887410	0.82667595	-1.90449398	H	-7.05800110	-1.12750086	3.31369541
H	3.56789444	0.98285590	-2.93710741	C	-4.03511850	-2.34558633	4.50090450
C	-4.48592883	0.07004614	-1.25707819	H	-3.74008348	-3.11432371	5.21917644
H	-4.63957905	-0.74034911	-1.97513014	H	-3.62929821	-1.39603250	4.86851958
H	-5.37159120	0.07376249	-0.61380015	C	-5.37128100	-1.47169243	2.01714703
C	7.60193138	0.53725730	1.07401013	H	-5.80716562	-2.39766325	1.62487512
C	-0.37776244	-1.64209997	-0.44448487	H	-5.64271845	-0.67183223	1.32513012
H	-0.61607938	-2.14627225	-1.38851773	C	-3.84114900	-1.60801226	2.10214171
H	-0.51540392	-0.57347010	-0.60432539	H	-3.43111112	-0.64820701	2.43977205
C	-3.29966151	-3.19916132	-1.95130096	C	-5.55717848	-2.24493541	4.41325595
H	-3.07640293	-2.23419668	-2.41447044	H	-5.97736861	-2.01252964	5.39459475
H	-2.34177191	-3.71232914	-1.81712967	H	-5.96997806	-3.21619383	4.11399051
C	5.17035361	0.48006521	-1.62320817	H	-0.96507165	6.21792914	-0.36609320
H	5.88396390	0.35157338	-2.42958304	H	-2.78018348	5.19427467	-1.53109361
C	-1.03442211	-3.65522653	0.92967793	H	-3.55551484	5.67709634	3.70918983
H	-1.22821943	-4.21381441	0.00794252	H	-6.14925263	2.64489847	-4.69534373
H	-1.69755192	-4.06526009	1.69271585				

Table S6. Cartesian Coordinates of the Optimized Structure for **D** in Toluene (PCM) at the GD3-M06-2X/6-311G(d) Level of Theory

atom	X	Y	Z	atom	X	Y	Z
S	0.66190076	0.37124665	0.13733056	C	3.68282594	-0.15569024	2.66661930
N	-6.09442261	-0.19871163	-0.10154054	H	2.89850040	-0.72612321	2.16215939
C	-1.89556252	-0.60041192	-0.43905760	H	3.34901072	-0.06664126	3.70358795
C	-0.44255831	-0.75270806	-0.57520270	C	-8.33479520	-3.05419884	2.04254711
C	0.24030464	-1.74764220	-1.23614957	H	-8.91081623	-3.78808715	2.59366739
H	-0.25657260	-2.54353407	-1.77674269	C	-6.37222760	1.32736854	-1.98828411
C	4.72789050	-0.85418839	-0.66666781	H	-5.56802736	0.81596026	-2.50526381
C	3.75943268	1.22068024	2.04751464	C	4.81127241	-2.42830510	2.38175404
C	-6.75437258	0.89494864	-0.71529790	H	4.18178243	-2.85299122	3.17167522
C	6.97192333	-2.40789244	-1.49708296	H	5.77780698	-2.93955399	2.43658855
C	-6.36563728	-1.67373446	1.82591293	C	6.72620844	-1.14836022	-2.04290737
H	-5.41255032	-1.32743343	2.20973217	H	7.39443691	-0.75939444	-2.80749883
C	-4.69425468	-0.33310279	-0.21148807	C	-7.79772908	1.55007799	-0.05553916
C	-6.84708221	-1.15951621	0.61873194	H	-8.09338655	1.21820501	0.93337308
C	4.97535323	-2.12940220	-0.11778589	C	4.14591653	4.78995958	3.32017646
C	-3.86469915	0.79286843	-0.17773682	H	3.99766862	5.73827415	2.80200147

H	-4.30448142	1.77683813	-0.06330094	H	5.15461392	4.79249545	3.74168006
C	-2.49142748	0.65805749	-0.29932953	H	3.44462162	4.74650513	4.15658982
H	-1.87227175	1.54888945	-0.29005481	C	5.40650484	0.96473610	-2.29315908
C	3.91067092	2.31570044	2.88590435	H	5.66378410	1.76513446	-1.59314948
H	3.97999142	2.15055080	3.95879058	H	6.06395592	1.06641504	-3.16122991
C	-7.10322220	-2.62083933	2.52458007	C	8.14886708	-3.22803854	-1.95514592
H	-6.71678496	-3.01147227	3.45919451	H	8.24556830	-4.14501147	-1.37264434
C	6.08528256	-2.87216918	-0.53788608	H	9.08043182	-2.66547682	-1.85858469
H	6.25093959	-3.85101026	-0.09306844	H	8.04557057	-3.50707827	-3.00696324
C	3.95593319	3.62003948	2.39122103	C	-8.08045192	-1.60024510	0.13131623
C	5.64179165	-0.38297435	-1.63829050	H	-8.45401577	-1.20463182	-0.80647773
C	2.05250581	-0.48341646	-0.45667815	C	-8.81934746	-2.53459737	0.84587523
C	3.65863371	1.38866691	0.63975670	H	-9.77485874	-2.86689974	0.45593907
C	1.64422152	-1.59029430	-1.16551671	C	3.96804181	1.06263334	-2.70729911
H	2.35413097	-2.26373820	-1.63309019	H	3.61531963	0.27688514	-3.37207198
C	-4.10701689	-1.59524408	-0.35469882	C	3.44758451	3.07797766	-1.31207094
H	-4.73642112	-2.47735867	-0.37736451	H	2.66093970	3.83521155	-1.35756941
C	4.16691098	-2.72746321	1.01336583	H	4.35678264	3.57649903	-1.66483907
H	4.11421835	-3.81189211	0.88174959	C	-7.01770642	2.40402888	-2.58263702
H	3.13817931	-2.37024023	0.99166695	H	-6.70977109	2.72893705	-3.57009135
C	-2.73155527	-1.72318850	-0.45470162	C	-8.44913929	2.61471555	-0.66514875
H	-2.29752141	-2.71333254	-0.53876630	H	-9.25701853	3.11325491	-0.14147498
C	3.64560550	2.70375492	0.15340788	C	3.09348889	1.95358238	-2.24137891
C	5.01536680	-0.93295856	2.62462294	H	2.05289136	1.86193070	-2.54003450
H	5.65606504	-0.52604363	1.83808743	C	-8.06146187	3.05155233	-1.92834165
H	5.55472702	-0.77750142	3.56278087	H	-8.56739215	3.88691895	-2.39770677
C	3.80431665	3.78787794	1.02587804	B	3.48682175	0.05965736	-0.23416013
H	3.79675008	4.79342052	0.61080746				

Table S7. Cartesian Coordinates of the Optimized Structure for **D**·PCy_3 in Toluene (PCM) at the GD3-M06-2X/6-311G(d) Level of Theory

atom	X	Y	Z	atom	X	Y	Z
S	0.59318565	1.66692862	0.09060384	H	-3.51145006	5.98335694	-2.20813939
P	-2.79637079	-2.25841464	0.53431359	H	-3.61615209	4.86520779	-0.86527714
N	7.20717780	0.02547767	-0.08400997	C	-1.77387711	5.96656212	-0.91427761
C	-0.37085140	1.17884096	-2.20498402	H	-2.16210192	6.79655885	-0.31381490
H	-1.06409264	1.07431225	-3.03325548	H	-1.09021677	6.40104485	-1.65317904
C	3.11476669	0.84031639	-0.84328253	C	7.58457363	1.98475983	1.32138771
C	3.58939478	0.66024554	0.46219649	H	6.86586631	2.53604142	0.72572848
H	2.89658789	0.70887896	1.29596928	C	8.79410475	-0.05222819	1.77171428
C	-3.31573294	2.53798729	1.75240323	H	9.00872280	-1.08761076	1.53239049
C	-0.78577743	1.62188271	-0.97441549	C	1.72625756	-3.63854520	0.22780676
C	-3.37503922	1.90808354	-1.65358672	H	2.77661104	-3.73056664	0.51566587
C	1.69460302	1.07239599	-1.12236516	H	1.60062272	-4.21138977	-0.69920519
C	1.01765254	0.86546357	-2.29445359	C	-3.87464908	-4.48654065	-2.80216979
H	1.48955301	0.47600655	-3.18933518	H	-4.83400973	-3.98089808	-2.96423890
C	-5.25208295	0.91963688	-2.89594311	H	-3.39431510	-4.57269224	-3.77952489
H	-5.94467969	0.08775310	-3.01592237	C	0.82364875	-4.23363756	1.30742428
C	-4.29522335	0.86303831	-1.87281808	H	1.01246165	-3.73326621	2.26474572
C	-3.42899767	2.97054406	-2.59571570	H	1.04445303	-5.29384903	1.45503220
C	-2.45200549	4.11732571	-2.52678983	C	8.22135674	2.59390617	2.39484354
H	-2.27216442	4.50577996	-3.53439741	H	7.99290177	3.62747941	2.62954293
H	-1.49479458	3.73754477	-2.17499495	C	9.43835662	0.57106108	2.83260460
C	-2.45135127	3.03761029	0.75626938	H	10.15808213	0.01099670	3.41918152
C	-3.65583906	3.25112312	2.89078144	C	-3.86875282	-4.91429501	0.13137852
H	-4.32447050	2.81626737	3.63205149	H	-2.91004757	-5.41518245	0.28691837
C	-2.67418175	0.56852864	0.56347621	H	-4.35381660	-4.83641187	1.10619113
H	-1.80250561	0.35601512	1.19907125	B	-2.28089977	1.87081559	-0.40450317
C	-3.11824336	4.52279969	3.10200614	C	9.15483430	1.89505133	3.15443546
C	-0.98701024	-2.57667059	0.73927088	H	9.65445541	2.37585193	3.98710268

H	-0.75108346	-2.04345756	1.67168063	C	-4.11763735	-5.87043834	-2.19920098
C	-4.38337696	3.00484872	-3.60687965	H	-3.16301575	-6.40594614	-2.13118596
H	-4.38369520	3.84398969	-4.30072971	H	-4.76349918	-6.46045875	-2.85375877
C	-2.99850119	-0.63793158	-0.31934448	C	-4.73526485	-5.76443514	-0.80448984
H	-2.21841804	-0.68565280	-1.09107464	H	-4.87181704	-6.75784091	-0.37041095
C	-3.79329334	1.13536019	1.45639488	H	-5.73129521	-5.31235542	-0.87989388
H	-4.73781452	1.19395728	0.90184004	C	9.23596844	-0.62848914	-1.27758435
H	-4.00425307	0.58712243	2.38154266	H	9.72925975	0.26359744	-0.90870677
C	4.92573262	0.39090136	0.71517487	C	7.27312403	-2.02253386	-1.40933168
H	5.26765718	0.24410447	1.73371415	H	6.24310650	-2.21936518	-1.13381355
C	5.84108149	0.30216743	-0.33583881	C	9.91693040	-1.51933317	-2.09694052
C	-5.32956337	1.99292696	-3.76634960	H	10.94734069	-1.31268729	-2.36385880
C	-0.98779703	5.01129560	0.00393279	C	9.28354751	-2.65725475	-2.58784203
H	-0.43627236	4.28189123	-0.58889821	H	9.81607472	-3.34601940	-3.23293700
H	-0.23738444	5.58889493	0.55295065	C	7.95748076	-2.89876246	-2.24165787
C	-2.24235564	5.02050364	2.14500746	H	7.45135035	-3.78331134	-2.61209802
H	-1.79391784	6.00008439	2.30388145	C	-3.02235576	-3.23179151	3.22179306
C	-1.90038480	4.30949389	0.98436333	H	-1.93227067	-3.21693110	3.29219287
C	-3.65472128	-3.52246449	-0.48550564	H	-3.31178285	-4.23418059	2.89128753
H	-4.65057976	-3.07520306	-0.61275560	C	-5.61341955	-1.83418431	3.58370745
C	4.04603157	0.75927438	-1.88762435	H	-5.29036615	-0.84895958	3.93974937
H	3.72254284	0.93469637	-2.90750483	H	-6.70434855	-1.80821609	3.53132508
C	-4.31372948	-0.43208194	-1.06692240	C	-3.61824120	-2.96817870	4.60854937
H	-4.49170734	-1.24986342	-1.77068842	H	-3.28303312	-3.74626462	5.29859983
H	-5.16743187	-0.43257404	-0.38272764	H	-3.22878611	-2.01723313	4.99005722
C	7.86355540	0.65231880	1.00133144	C	-5.04314481	-2.07211663	2.18350118
C	-0.10219506	-1.97297707	-0.36659299	H	-5.46084912	-3.00170524	1.77946756
H	-0.33265228	-2.44462224	-1.32953752	H	-5.35666925	-1.26468156	1.51881303
H	-0.28987514	-0.90563643	-0.47755660	C	-3.50858700	-2.16434340	2.22561988
C	-3.00575643	-3.62910038	-1.87942472	H	-3.11630257	-1.20317786	2.57970103
H	-2.84706897	-2.64164333	-2.32132743	C	-5.14418637	-2.90829684	4.56340555
H	-2.01860060	-4.09369959	-1.78477611	H	-5.54412204	-2.71173341	5.56092675
C	5.38405841	0.49715223	-1.64227692	H	-5.53777652	-3.88327385	4.25080952
H	6.08909846	0.45028479	-2.46489409	C	-6.37137176	2.06740377	-4.85228993
C	-0.65163128	-4.06778397	0.93435324	H	-5.91874042	2.28850733	-5.82200809
H	-0.83514246	-4.60094851	-0.00444795	H	-7.09688922	2.85912546	-4.64590342
H	-1.28498276	-4.53319586	1.69102277	H	-6.92073569	1.12858485	-4.94233763
C	1.37799497	-2.17436730	-0.03040372	C	-3.47796401	5.31805000	4.33143126
H	1.98537115	-1.76818895	-0.84221050	H	-2.83030040	6.18892166	4.44542903
H	1.61868241	-1.57600977	0.85698098	H	-3.39078574	4.71235023	5.23695717
C	7.90617830	-0.87488112	-0.92093867	H	-4.51001860	5.67675338	4.27989922
C	-2.93328200	5.26445013	-1.61913602				

Table S8. Cartesian Coordinates of the Optimized Structure for PCy_3 in Toluene (PCM) at the GD3-M06-2X/6-311G(d) Level of Theory

atom	X	Y	Z	atom	X	Y	Z
P	0.02186504	-0.12479321	-1.08701383	C	0.43454963	4.28038817	0.70034155
C	-1.46004874	-1.08507681	-0.46649547	H	1.53130746	4.26067994	0.68170361
H	-1.24513691	-2.11920898	-0.76710488	H	0.14368792	5.26614761	1.07389325
C	-2.72489288	-0.66413737	-1.23097808	C	-0.06880138	3.18345234	1.63903710
H	-2.54138090	-0.70813279	-2.30830155	H	-1.15982234	3.25543821	1.72964309
H	-2.97027753	0.37929242	-0.99595439	H	0.34056959	3.32367367	2.64367882
C	-3.91882366	-1.54886002	-0.85884275	C	0.30318231	1.79257964	1.11467328
H	-4.81375090	-1.21300820	-1.39033672	H	1.39605144	1.70640844	1.10324457
H	-3.72008052	-2.57463360	-1.19214962	H	-0.06082904	1.02692822	1.80322805
C	-4.16463435	-1.54895082	0.65092040	C	1.42008418	-0.84616138	-0.07517602
H	-4.45801547	-0.54038975	0.96783217	H	1.27838427	-0.60738748	0.98505848
H	-4.99781330	-2.21175931	0.90049523	C	1.48516721	-2.37422663	-0.20900056
C	-2.90509351	-1.96479483	1.41268443	H	0.56922631	-2.83230861	0.17431434
H	-2.65925481	-3.00542134	1.16764673	H	1.55348592	-2.64316819	-1.27134746

H	-3.08230883	-1.92813752	2.49141373	C	2.68796197	-2.95149798	0.54466317
C	-1.71944983	-1.06743872	1.04344203	H	2.72512064	-4.03608689	0.40845639
H	-1.94620051	-0.04087149	1.35668915	H	2.55303951	-2.77355844	1.61869255
H	-0.82618290	-1.37747440	1.59528088	C	4.00064006	-2.31173337	0.09204733
C	-0.24798915	1.56908389	-0.30117207	H	4.83808934	-2.71482458	0.66826046
H	-1.34119394	1.66846727	-0.24833519	H	4.18502978	-2.57036708	-0.95778337
C	0.25415299	2.67521000	-1.24478075	C	3.93960415	-0.78996908	0.22807230
H	-0.16810221	2.52958313	-2.24265065	H	3.84728268	-0.52232322	1.28800619
H	1.34288159	2.59084561	-1.35597606	H	4.86736648	-0.33682229	-0.13248514
C	-0.09615284	4.06932692	-0.71797909	C	2.74548241	-0.22306721	-0.54274987
H	0.29878613	4.83500919	-1.39180543	H	2.87266514	-0.43051196	-1.61250280
H	-1.18716321	4.18494245	-0.71451419	H	2.71693195	0.86611770	-0.44332445

Table S9. Cartesian Coordinates of the Optimized Structure for **10** at the M06-2X/6-31G(d) Level of Theory

atom	X	Y	Z	atom	X	Y	Z
C	0.72506322	0.88747768	0.01441109	C	-5.17816079	0.23746253	-0.25791932
C	-0.71038960	-1.48887243	-0.01782378	H	-6.01627663	0.89745552	-0.44622150
C	-0.72506489	0.88747517	-0.01441135	C	-5.31656302	-1.07993395	0.06216892
C	1.47174912	-0.34491059	0.02871350	S	-3.79318360	-1.84272081	0.30049479
C	0.71039509	-1.48887025	0.01781983	C	2.93299457	-0.36586129	0.04566646
C	-1.47174655	-0.34491483	-0.02871628	C	3.81653812	0.64948298	0.31735887
H	1.20580629	-2.45530951	0.04185314	H	3.49423435	1.65440547	0.55168260
H	-1.20579770	-2.45531325	-0.04185710	C	5.17816562	0.23746503	0.25790564
N	-1.24088129	2.11312014	-0.02289171	H	6.01628293	0.89745899	0.44619772
N	1.24087353	2.11312531	0.02289149	C	5.31656704	-1.07991996	-0.06223044
S	-0.00000632	3.15943502	0.00000278	S	3.79318315	-1.84272585	-0.30046589
C	-2.93299173	-0.36586517	-0.04566721	H	-6.22792102	-1.64943041	0.17929219
C	-3.81653339	0.64948477	-0.31734296	H	6.22792514	-1.64940708	-0.17939863
H	-3.49422762	1.65441033	-0.55165160				

Table S10. Cartesian Coordinates of the Optimized Structure for **3a** at the M06-2X/6-31G(d) Level of Theory

atom	X	Y	Z	atom	X	Y	Z
C	-0.72295872	-2.43826143	0.05381963	C	-6.34527724	3.5802391	-2.09126271
C	0.70803318	-0.06020131	-0.05678874	H	-6.17329013	3.85719125	-3.12896295
C	0.72354117	-2.43821915	-0.05497446	C	-7.01600617	2.498484	2.00578211
C	-1.46728943	-1.20541349	0.10887202	H	-7.02446793	3.39648047	2.62907279
C	-0.70759716	-0.06024190	0.05530377	H	-6.22592136	1.84008669	2.38479483
C	1.46779327	-1.20533503	-0.11016224	H	-7.97068305	1.97932602	2.14380231
H	-1.20243623	0.90532621	0.10569915	C	-6.42069452	-0.20806954	-2.67318047
H	1.20282334	0.90538444	-0.10732313	H	-5.62587728	-0.92135177	-2.88336273
N	1.23806779	-3.66401111	-0.09080891	C	-6.06043901	1.24219764	-2.86850929
N	-1.23739361	-3.66408688	0.08981340	H	-6.5512671	1.60962393	-3.77978608
S	0.00037421	-4.71020029	-0.00043686	H	-4.98488487	1.29726072	-3.07622763
C	2.92566107	-1.17782818	-0.20840650	B	-6.65913909	0.32674955	0.0796112
C	3.79060443	-2.20177926	-0.53879720	C	8.05309066	-0.28612052	-0.53994274
H	3.45319110	-3.20674284	-0.75122040	C	8.31767812	-0.72818409	-1.85173835
C	5.14135630	-1.78425444	-0.56810933	C	9.1073997	-0.33437399	0.40678528
H	5.97423704	-2.44020972	-0.80405555	C	6.59900693	1.83839766	0.41404159
C	5.33254316	-0.45539854	-0.25754586	C	6.78695302	2.85167712	-0.55566857
S	3.80390827	0.29062484	0.08187951	C	9.60029978	-1.18488497	-2.18684748
C	-2.92514828	-1.17797859	0.20727478	H	9.78684524	-1.51906012	-3.20506434
C	-3.79003820	-2.20204240	0.53748750	C	10.36640334	-0.80218784	0.05248047
H	-3.45256412	-3.20705450	0.74957845	H	11.15113716	-0.84809866	0.80326987
C	-5.14078687	-1.78456020	0.56709452	C	6.35846635	2.22592349	1.74358907
H	-5.97363023	-2.44057058	0.80300908	C	10.62065057	-1.2204381	-1.2517872
C	-5.33203129	-0.45560180	0.25695394	H	11.60623918	-1.58268908	-1.52868436
S	-3.80347774	0.29054051	-0.08242608	C	7.29920723	-0.7067806	-2.97180092
C	-8.05235314	-0.28645146	0.54106645	H	7.57894662	0.03558554	-3.72856525
C	-8.31604836	-0.72869569	1.85298415	H	6.29164634	-0.47386247	-2.62953983

C	-9.10730117	-0.33451257	-0.40495563	H	7.26539196	-1.68020991	-3.47259729
C	-6.59936349	1.83807028	-0.41400349	C	8.82312981	0.03643729	1.85359491
C	-6.78792030	2.85153859	0.55532966	C	7.59889401	-0.71243387	2.30032122
C	-9.59838797	-1.18561092	2.18885718	H	7.6773593	-1.79611158	2.21321022
H	-9.78423878	-1.51996365	3.20714226	C	6.74801938	4.19482594	-0.18939695
C	-10.36602974	-0.80250834	-0.04987961	H	6.89094587	4.95661942	-0.9514533
H	-11.15128544	-0.84827781	-0.80013139	C	6.53551408	4.56517746	1.13410716
C	-6.35955153	2.22521263	-1.74382629	H	6.51967877	5.61395989	1.41502726
C	-10.61934927	-1.22110293	1.25445565	C	6.34269083	3.58110722	2.0904243
H	-11.60471266	-1.58352448	1.53193285	H	6.17008755	3.85830552	3.12796179
C	-7.29691642	-0.70700028	2.97243483	C	7.01577238	2.49824969	-2.00590963
H	-7.57602858	0.03585796	3.72895230	H	7.02476993	3.39610145	-2.62939919
H	-6.28949312	-0.47450288	2.62946178	H	6.22573488	1.83994656	-2.38519614
H	-7.26306818	-1.68015840	3.47374602	H	7.97040491	1.97885472	-2.14329508
C	-8.82415414	0.03677314	-1.85186866	C	6.41905127	-0.20731214	2.67281101
H	-9.68431736	-0.24563787	-2.47000901	H	5.62324372	-0.91989424	2.88158667
H	-8.68447669	1.11743680	-1.96020020	C	6.06067275	1.24328499	2.86901816
C	-7.60068972	-0.71243373	-2.30014840	H	6.55402904	1.61027242	3.77910544
H	-7.67990354	-1.79613592	-2.21398095	H	4.98565915	1.29956841	3.07911315
C	-6.75035787	4.19458363	0.18846325	B	6.65954142	0.32698189	-0.07926243
H	-6.89371199	4.95657754	0.95023974	H	9.68264553	-0.24649014	2.47240134
C	-6.53874227	4.56454266	-1.13527598	H	8.68368674	1.11712192	1.96217987
H	-6.52405538	5.61321512	-1.41666811				

Table S11. Cartesian Coordinates of the Optimized Structure for **3a**·$2PCy_3$ at the M06-2X/6-31G(d) Level of Theory

atom	X	Y	Z	atom	X	Y	Z
S	-3.41408427	-1.66022747	0.51527085	H	1.09000588	0.51641114	1.06621730
P	-7.40241606	1.55476956	-0.18559454	H	-1.09298084	-0.50660796	1.06662606
C	-4.74717387	-2.11752578	-1.59822016	N	1.13014580	0.51451677	-3.49973722
H	-5.54157019	-2.42986016	-2.27135660	N	-1.12507626	-0.52556325	-3.49931962
C	-6.84116855	-2.34194447	3.05784749	S	0.00347299	-0.00801963	-4.54791175
C	-4.88009474	-2.23399940	-0.23326654	S	3.41457501	1.66198215	0.51418150
C	-7.41860819	-3.19282830	-0.22107096	P	7.40140273	-1.55468741	-0.18441072
C	-2.66654932	-1.23402125	-1.00866373	C	4.74795241	2.11745933	-1.59951970
C	-3.51371744	-1.55737066	-2.04254342	H	5.54246137	2.42916067	-2.27281772
H	-3.25527412	-1.39801553	-3.08092321	C	6.84197782	2.34441919	3.05638683
C	-9.56486242	-3.04732089	-1.41924904	C	4.88073298	2.23501493	-0.23465030
H	-10.39903992	-2.43286423	-1.75522248	C	7.41947710	3.19291721	-0.22318519
C	-8.52847608	-2.45338456	-0.68724467	C	2.66726149	1.23438800	-1.00948860
C	-7.40457843	-4.56613651	-0.57429695	C	3.51457553	1.55688471	-2.04352695
C	-6.25589645	-5.44783771	-0.16524875	H	3.25635384	1.39652075	-3.08179977
H	-6.36375902	-6.45421462	-0.58068362	C	9.56527137	3.04563771	-1.42197804
H	-5.30496837	-5.03294316	-0.51270877	H	10.39907818	2.43059253	-1.75779492
C	-5.98540797	-3.06163475	2.19360823	C	8.52889001	2.45266325	-0.68918736
C	-6.92104574	-2.58694185	4.42081758	C	7.40591619	4.56597781	-0.57739449
H	-7.59436426	-2.00633564	5.04842172	C	6.25770163	5.44845468	-0.16869865
C	-6.73760263	-0.96620094	1.10840099	H	6.36618678	6.45464711	-0.58441556
H	-5.84115859	-0.43275269	1.44224664	H	5.30657215	5.03400670	-0.51617100
C	-6.10292528	-3.56869781	4.97827878	C	5.98636891	3.06379303	2.19174315
C	-6.10594545	2.14740220	0.96983919	C	6.92206001	2.59033463	4.41918055
H	-6.27628734	1.52061590	1.85792246	H	7.59525640	2.00994820	5.04711807
C	-8.44227745	-5.14654980	-1.30214222	C	6.73775715	0.96744487	1.10785723
H	-8.38925987	-6.20561450	-1.54407282	H	5.84112954	0.43460569	1.44218500
C	-7.35439814	-0.29867524	-0.12038515	C	6.10431315	3.57275126	4.97603438
H	-6.66801292	-0.51357545	-0.95705538	C	6.10493403	-2.14591655	0.97175398
C	-7.60089282	-1.23822968	2.34987815	H	6.27571440	-1.51846245	1.85928310
H	-8.60096315	-1.60046367	2.07248489	C	8.44362426	5.14542598	-1.30600248
H	-7.74347698	-0.37225928	3.01486828	H	8.39098075	6.20434157	-1.54866580
C	-9.53484294	-4.39648060	-1.72335266	C	7.35409397	0.29880700	-0.12054199

C	-4.03209478	-4.73866183	2.03563138
H	-3.95839908	-4.42158770	0.99709894
H	-3.06092170	-4.55277265	2.50859310
C	-5.20468286	-4.24516802	4.16592421
H	-4.52914957	-4.97593031	4.60794445
C	-5.12010933	-3.99934432	2.78574396
C	-6.92348255	2.01738221	-1.90979112
H	-6.00925032	1.42043971	-2.06067732
C	-8.69283505	-0.95680086	-0.45497758
H	-9.14724817	-0.52009445	-1.35198339
H	-9.41131958	-0.77610270	0.35643200
C	-4.72284537	1.77265891	0.39231470
H	-4.50248266	2.41571434	-0.47004325
H	-4.70429398	0.73649007	0.03419824
C	-6.55195725	3.49524783	-2.13135742
H	-5.76646020	3.80091958	-1.43542965
H	-7.41597300	4.14110509	-1.93954646
C	-6.13363592	3.61200552	1.43490396
H	-5.97417481	4.29126761	0.58875499
H	-7.10561170	3.86705598	1.87041727
C	-3.64077832	1.98132764	1.45092465
H	-2.66538313	1.72129649	1.02561925
H	-3.81847868	1.28868805	2.28497652
C	-3.65013208	3.42758906	1.95214110
H	-2.89671110	3.56623897	2.73390496
H	-3.37395967	4.09589440	1.12458833
C	-6.07321081	3.71890506	-3.56991135
H	-5.14829523	3.14949752	-3.73329582
H	-5.82499355	4.77599664	-3.70950138
C	-5.03045720	3.82780497	2.48105596
H	-5.26616803	3.22525163	3.36873730
H	-5.03003998	4.87554344	2.79939307
C	-7.96116145	1.53146814	-2.93898738
H	-8.91940514	2.04454251	-2.77124031
H	-8.13928601	0.45887490	-2.81826472
B	-6.20597074	-2.48976252	0.66456105
C	-7.12927265	3.27250922	-4.58022104
H	-8.03234543	3.88692835	-4.45709154
H	-6.77150194	3.43425834	-5.60197762
C	-7.48289968	1.80107503	-4.36972730
H	-8.25621481	1.48218369	-5.07582549
H	-6.59709935	1.18309917	-4.56765768
C	-9.40271352	3.61152636	-0.12036907
H	-8.69578293	4.29105053	0.37249780
H	-9.28957151	3.75750608	-1.19886955
C	-10.80554489	2.24247745	2.10613866
H	-10.97166046	2.04871201	3.17042415
H	-11.50140625	1.59742298	1.55257511
C	-10.83520655	3.97591797	0.28708623
H	-11.53698649	3.38061661	-0.31303618
H	-11.02443102	5.02690186	0.04569659
C	-9.36975197	1.86149934	1.73805430
H	-8.67419107	2.43530923	2.36551754
H	-9.19292485	0.80397896	1.95540064
C	-9.08750856	2.15286532	0.25147337
H	-9.75133771	1.50806181	-0.34486746
C	-11.09087001	3.70598209	1.76983473
H	-12.12283497	3.96357684	2.02848143
H	-10.43940482	4.35148251	2.37541921
H	-10.33893973	-4.85632175	-2.29069846
H	-6.14240105	-3.77897106	6.04361207
H	6.66764588	0.51334231	-0.95725209
C	7.60127346	1.23998171	2.34907097
H	8.60144349	1.60169310	2.07135860
H	7.74362738	0.37438926	3.01460707
C	9.53572317	4.39458971	-1.72704581
C	4.03346667	4.74113692	2.03275371
H	3.96007406	4.42378429	0.99428530
H	3.06219545	4.55520442	2.50550657
C	5.20619494	4.24893827	4.16330097
H	4.53093969	4.98022632	4.60487675
C	5.12140199	4.00215320	2.78331068
C	6.92193666	-2.01830170	-1.90818810
H	6.00799590	-1.42097959	-2.05934670
C	8.69271513	0.95618063	-0.45589677
H	9.14674435	0.51864699	-1.35269700
H	9.41130836	0.77577191	0.35548362
C	4.72187949	-1.77102222	0.39421622
H	4.50097806	-2.41475543	-0.46749796
H	4.70375929	-0.73517914	0.03515329
C	6.54956347	-3.49612395	-2.12867053
H	5.76406816	-3.80091288	-1.43235926
H	7.41327943	-4.14231305	-1.93663975
C	6.13203119	-3.61013339	1.43806838
H	5.97206382	-4.29005007	0.59254452
H	7.10397046	-3.86528974	1.87360534
C	3.64000305	-1.97817159	1.45331563
H	2.66462192	-1.71790545	1.02812790
H	3.81836290	-1.28494821	2.28674030
C	3.64871438	-3.42402258	1.95572818
H	2.89541235	-3.56163416	2.73778855
H	3.37199883	-4.09287675	1.12879950
C	6.07033478	-3.72046250	-3.56695578
H	5.14568137	-3.15066697	-3.73047014
H	5.82151945	-4.77751303	-3.70579403
C	5.02896876	-3.82449692	2.48464422
H	5.26518024	-3.22131710	3.37176699
H	5.02810695	-4.87196652	2.80386240
C	7.95967526	-1.53367798	-2.93792883
H	8.91763764	-2.04724271	-2.77008164
H	8.13850748	-0.46111777	-2.81793373
B	6.20667492	2.49092682	0.66305479
C	7.12637535	-3.27529241	-4.57783076
H	8.02913787	-3.89013691	-4.45455233
H	6.76823999	-3.43749019	-5.59938878
C	7.48088632	-1.80392108	-4.36837373
H	8.25419214	-1.48591821	-5.07488348
H	6.59538043	-1.18557168	-4.56645336
C	9.40079451	-3.61220019	-0.11807600
H	8.69362415	-4.29102096	0.37541420
H	9.28738119	-3.75890135	-1.19644738
C	10.80470048	-2.24220836	2.10717249
H	10.97108683	-2.04773606	3.17128634
H	11.50083188	-1.59795853	1.55301481
C	10.83317271	-3.97700508	0.28939329
H	11.53515771	-3.38248162	-0.31125981
H	11.02184371	-5.02825455	0.04872809
C	9.36905208	-1.86072304	1.73903710
H	8.67329618	-2.43363374	2.36710040
H	9.19283034	-0.80292716	1.95554890
C	9.08635686	-2.15311309	0.25274766
H	9.75034043	-1.50906389	-0.34422913

H	-4.19867516	-5.82250139	2.05147594	C	11.08918241	-3.70612181	1.77191010
H	-6.20125953	-5.52887284	0.92380449	H	12.12103709	-3.96409073	2.03062542
C	1.34317775	0.62552952	-1.04270012	H	10.43744015	-4.35082347	2.37804993
C	-1.34249732	-0.62512573	-1.04222813	H	10.33982129	4.85367069	-2.29500576
C	0.66173200	0.30299042	-2.27355090	H	6.14397530	3.78377228	6.04121278
C	0.64279881	0.30276996	0.09906706	H	4.19988700	5.82500382	2.04839635
C	-0.64406837	-0.29734791	0.09930186	H	6.20297232	5.52983134	0.92031675
C	-0.65891868	-0.30810833	-2.27331376				

Table S12. Cartesian Coordinates of the Optimized Structure for **11** at the M06-2X/6-31G(d) Level of Theory

atom	X	Y	Z	atom	X	Y	Z
C	-0.72421667	2.21121811	-0.04621848	H	-7.94909203	-4.83391186	-0.21492588
C	0.70841558	-0.16508812	0.04464986	H	-6.85238885	-5.29435022	-2.39739420
C	0.72421675	2.21121830	0.04621548	C	-7.62038454	0.09537976	0.47687286
C	-1.47127962	0.97894609	-0.09235620	C	-9.80177529	1.22781986	1.81526186
C	-0.70841449	-0.16508829	-0.04466285	C	-7.42000496	0.85222008	1.63574251
C	1.47128020	0.97894636	0.09234849	C	-8.91872642	-0.08898065	-0.00950664
H	-1.20163749	-1.13219584	-0.08313931	C	-9.99923191	0.46909881	0.66436797
H	1.20163892	-1.13219564	0.08312278	C	-8.50669700	1.41771169	2.29223135
N	1.23853736	3.43684435	0.07696492	H	-6.41165093	0.99497833	2.01117422
N	-1.23853829	3.43684399	-0.07695964	H	-9.07209522	-0.67023791	-0.91320294
S	-0.00000033	4.48440477	-0.00000134	H	-11.00189018	0.31858960	0.27603431
C	2.92717941	0.95949072	0.17326056	H	-8.33765196	2.00267783	3.19109094
C	3.79868020	1.97880205	0.46939561	H	-10.64724543	1.66731266	2.33414613
H	3.46556751	2.99028996	0.65556677	C	6.61785697	-1.76623645	0.79611310
C	5.15511107	1.56561426	0.54555253	C	6.78710113	-4.30789381	1.95026198
H	5.98638044	2.22274593	0.77243234	C	6.00564001	-2.02263038	2.02654242
C	5.32123819	0.23085278	0.29320153	C	7.31668236	-2.78905392	0.14595679
S	3.79627184	-0.53767867	-0.07177099	C	7.40474174	-4.04749648	0.72863598
C	-2.92717893	0.95949046	-0.17326667	C	6.08500194	-3.29067904	2.59145066
C	-3.79867996	1.97880203	-0.46940026	H	5.47198285	-1.22308624	2.53122738
H	-3.46556736	2.99029009	-0.65557099	H	7.78549720	-2.58857026	-0.81226226
C	-5.15511112	1.56561466	-0.54555469	H	7.94909045	-4.83391325	0.21492960
H	-5.98638076	2.22274680	-0.77243204	H	5.60389912	-3.47830160	3.54637072
C	-5.32123819	0.23085315	-0.29320367	H	6.85238002	-5.29435223	2.39739412
S	-3.79627100	-0.53767940	0.07176304	C	7.62038665	0.09537880	-0.47686827
N	-6.51607718	-0.48011478	-0.20523770	C	9.80178164	1.22781892	-1.81525034
N	6.51607718	-0.48011564	0.20523880	C	7.42001067	0.85222013	-1.63573787
C	-6.61785930	-1.76623527	-0.79611228	C	8.91872711	-0.08898255	0.00951472
C	-6.78710817	-4.30789201	-1.95026180	C	9.99923476	0.46909689	-0.66435648
C	-7.31668294	-2.78905278	-0.14595418	C	8.50670478	1.41771175	-2.29222326
C	-6.00564641	-2.02262883	-2.02654369	H	6.41165776	0.99497916	-2.01117224
C	-6.08501067	-3.29067720	-2.59145228	H	9.07209315	-0.67024055	0.91321102
C	-7.40474470	-4.04749504	-0.72863367	H	11.00189182	0.31858691	-0.27602016
H	-7.78549456	-2.58856940	0.81226650	H	8.33766249	2.00267868	-3.19108285
H	-5.47199063	-1.22308466	-2.53123000	H	10.64725342	1.66731172	-2.33413194
H	-5.60391102	-3.47829949	-3.54637400				

7. References

[S1] (a) M. O. Kitching, T. E. Hurst, V. Snieckus, *Angew. Chem. Int. Ed.* **2012**, *51*, 2925–2929; (b) T. Ikeuchi, S. Inuki, S. Oishi, H. Ohno, *Angew. Chem. Int. Ed.* **2019**, *58*, 7792–7796.

[S2] G. M. Sheldrick, *Acta Cryst.* **2015**, A71, 3–8.

[S3] G. M. Sheldrick, *Acta Cryst.* **2015**, C71, 3–8.

[S4] R. Oshimizu, N. Ando, S. Yamaguchi, *Angew. Chem. Int. Ed.* **2022**, *61*, e202209394.

[S5] M. Ito, E. Ito, M. Hirai, S. Yamaguchi, *J. Org. Chem.* **2018**, *83*, 8449–8456.

[S6] Gaussian 16, Revision B.01, M. J. Frisch, G. W. Trucks, H. B. Schlegel, G. E. Scuseria, M. A. Robb, J. R. Cheeseman, G. Scalmani, V. Barone, G. A. Petersson, H. Nakatsuji, X. Li, M. Caricato, A. V. Marenich, J. Bloino, B. G. Janesko, R. Gomperts, B. Mennucci, H. P. Hratchian, J. V. Ortiz, A. F. Izmaylov, J. L. Sonnenberg, D. Williams-Young, F. Ding, F. Lipparini, F. Egidi, J. Goings, B. Peng, A. Petrone, T. Henderson, D. Ranasinghe, V. G. Zakrzewski, J. Gao, N. Rega, G. Zheng, W. Liang, M. Hada, M. Ehara, K. Toyota, R. Fukuda, J. Hasegawa, M. Ishida, T. Nakajima, Y. Honda, O. Kitao, H. Nakai, T. Vreven, K. Throssell, J. A. Montgomery, Jr., J. E. Peralta, F. Ogliaro, M. J. Bearpark, J. J. Heyd, E. N. Brothers, K. N. Kudin, V. N. Staroverov, T. A. Keith, R. Kobayashi, J. Normand, K. Raghavachari, A. P. Rendell, J. C. Burant, S. S. Iyengar, J. Tomasi, M. Cossi, J. M. Millam, M. Klene, C. Adamo, R. Cammi, J. W. Ochterski, R. L. Martin, K. Morokuma, O. Farkas, J. B. Foresman, and D. J. Fox, Gaussian, Inc., Wallingford CT, 2016.

[S7] G. Scalmani, M. J. Frisch, *J. Chem. Phys.* **2010**, *132*, 114110.

[S8] S. Grimme, J. Antony, S. Ehrlich, H. Krieg, *J. Chem. Phys.* **2010**, *132*, 154104.

8. NMR Spectra

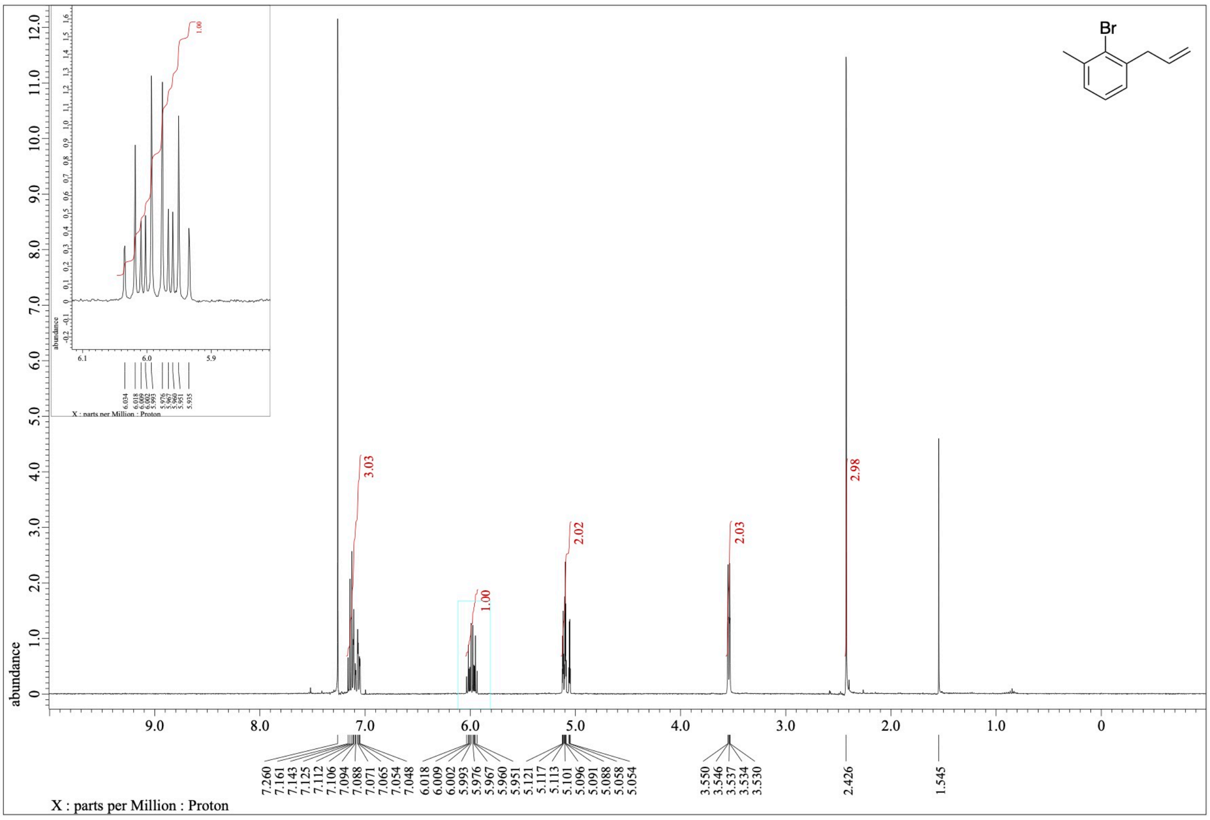

Figure S17. ^{1}H NMR spectrum of **4** (400 MHz, $CDCl_3$).

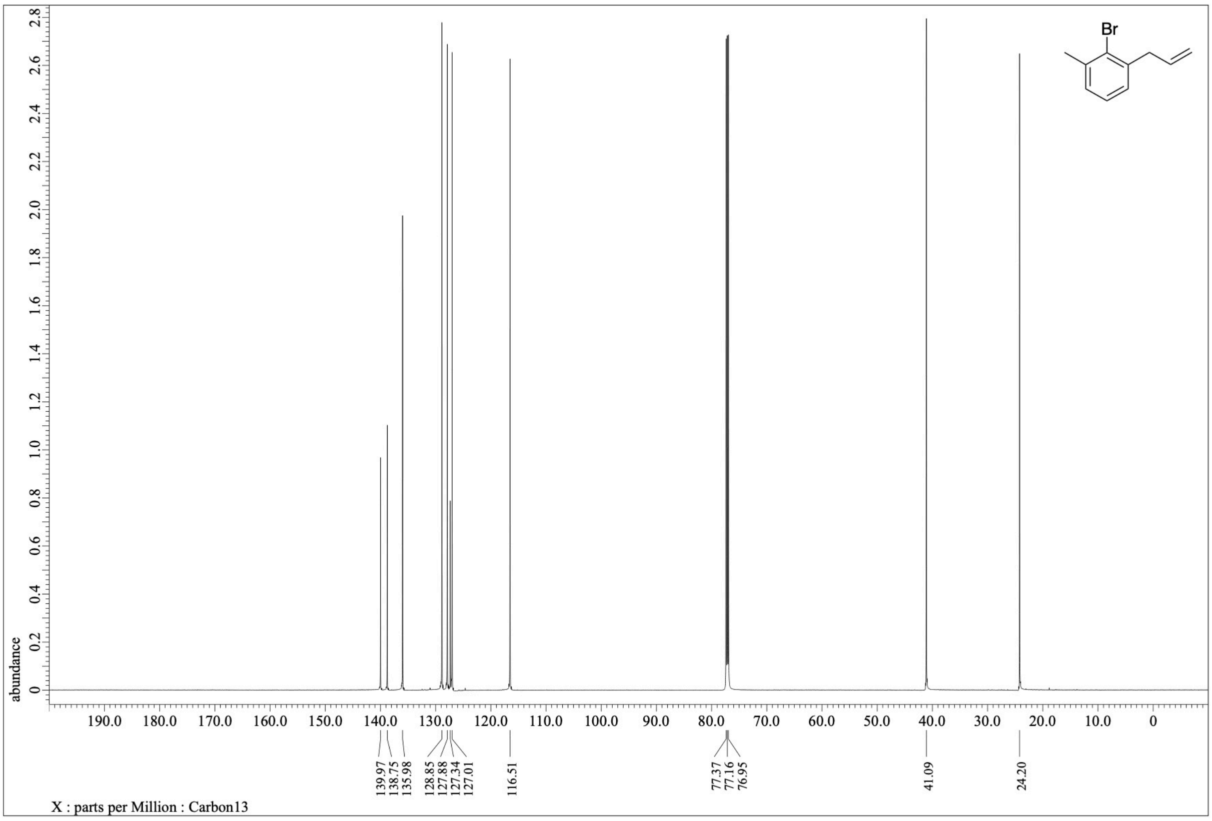

Figure S18. ^{13}C{^{1}H} NMR spectrum of **4** (150 MHz, $CDCl_3$).

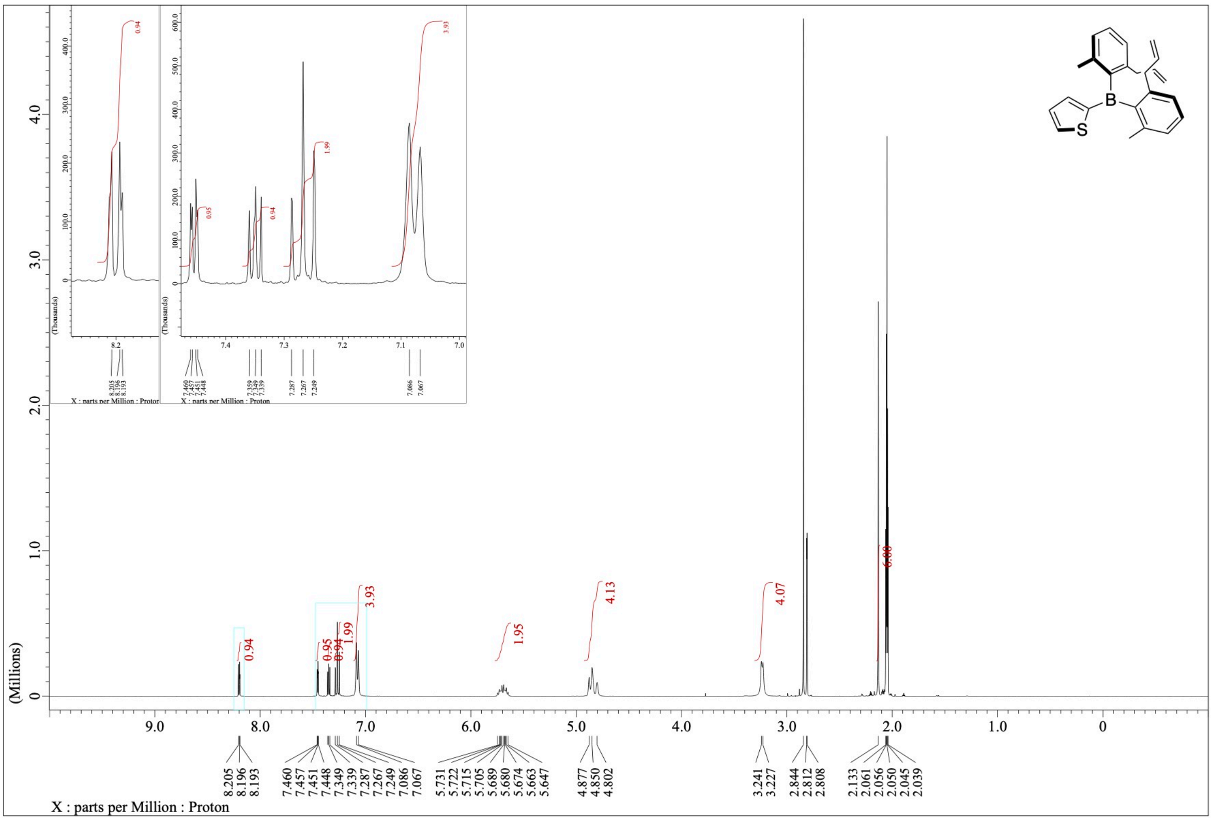

Figure S19. ^{1}H NMR spectrum of **5** (400 MHz, acetone-d_6).

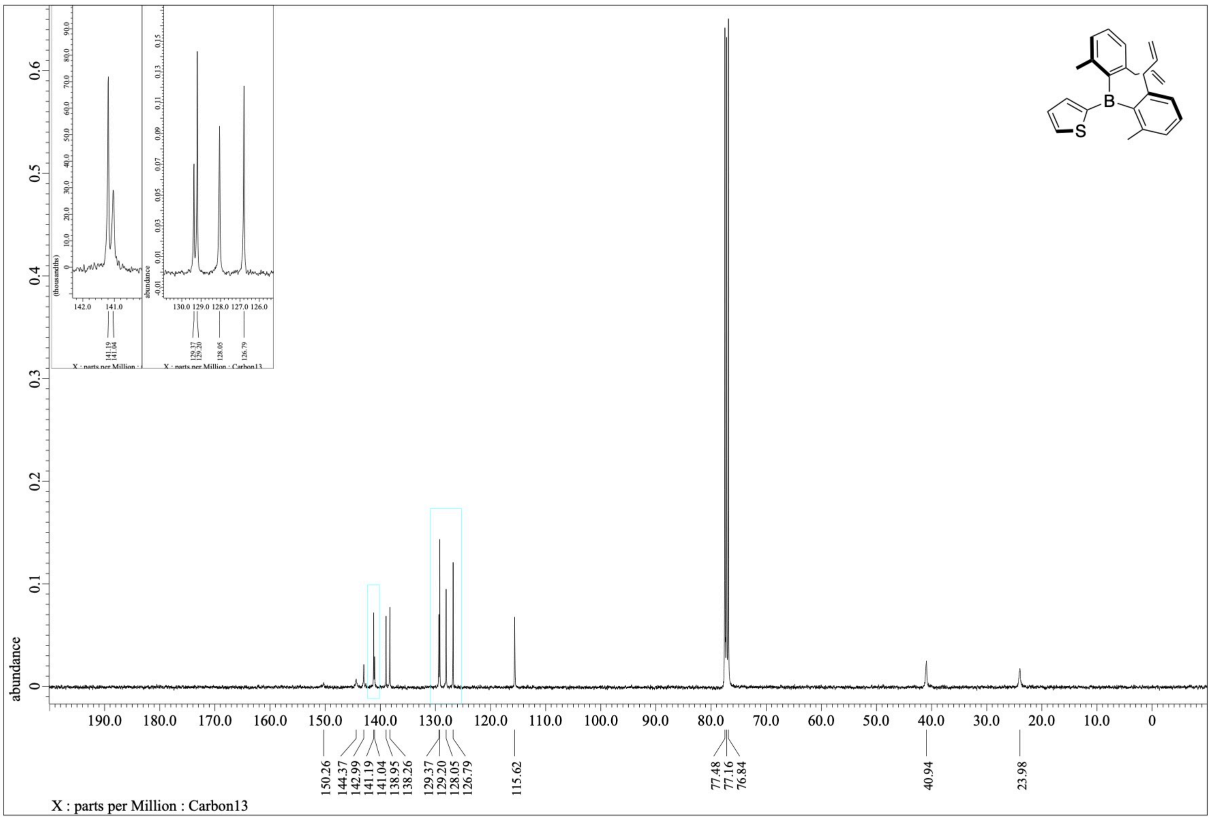

Figure S20. $^{13}C\{^{1}H\}$ NMR spectrum of **5** (100 MHz, $CDCl_3$).

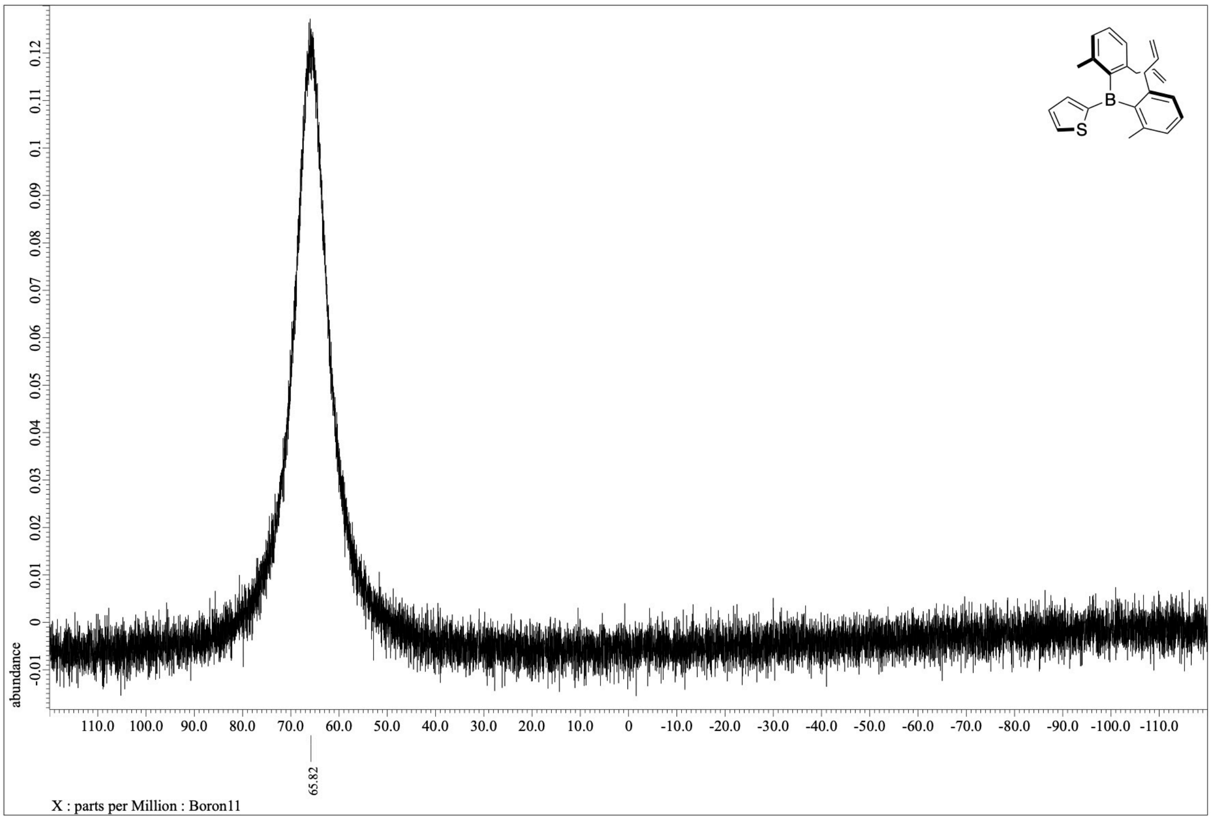

Figure S21. ^{11}B NMR spectrum of **5** (128 MHz, $CDCl_3$).

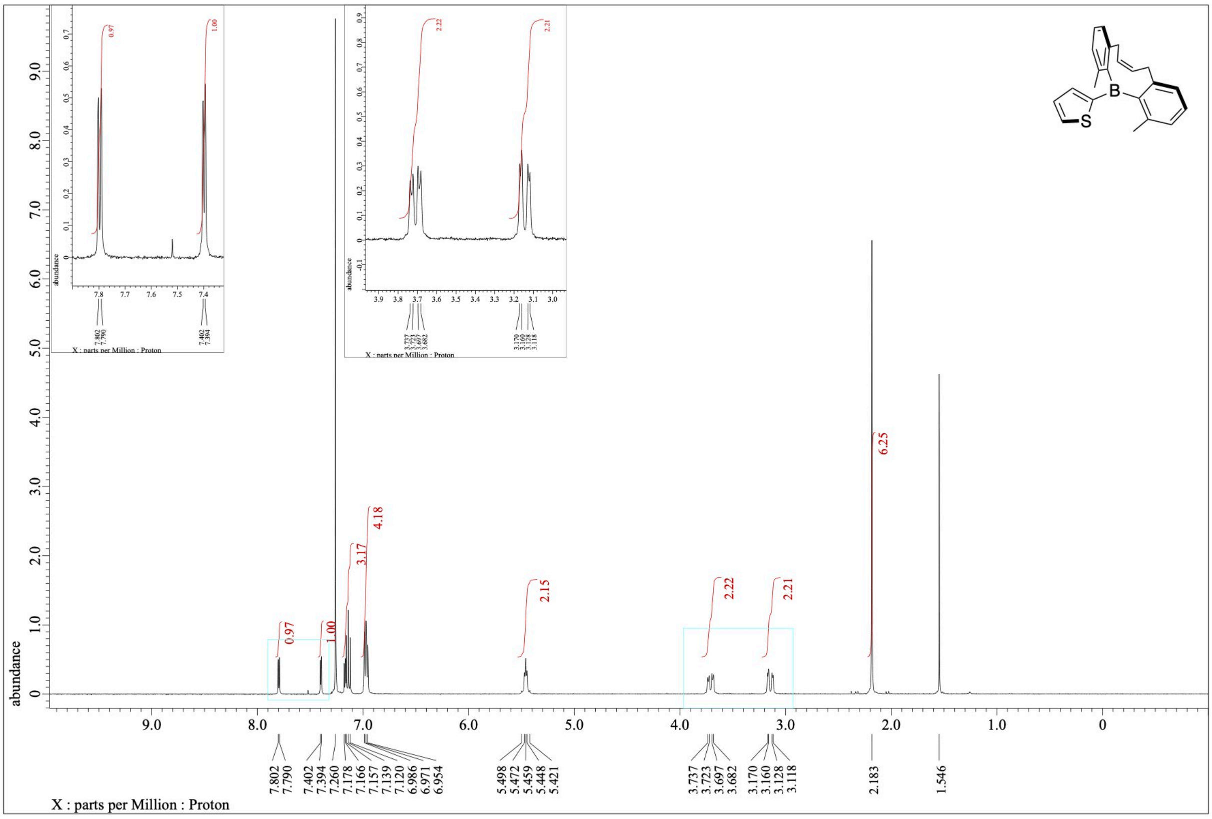

Figure S22. ^{1}H NMR spectrum of **1** (400 MHz, $CDCl_3$).

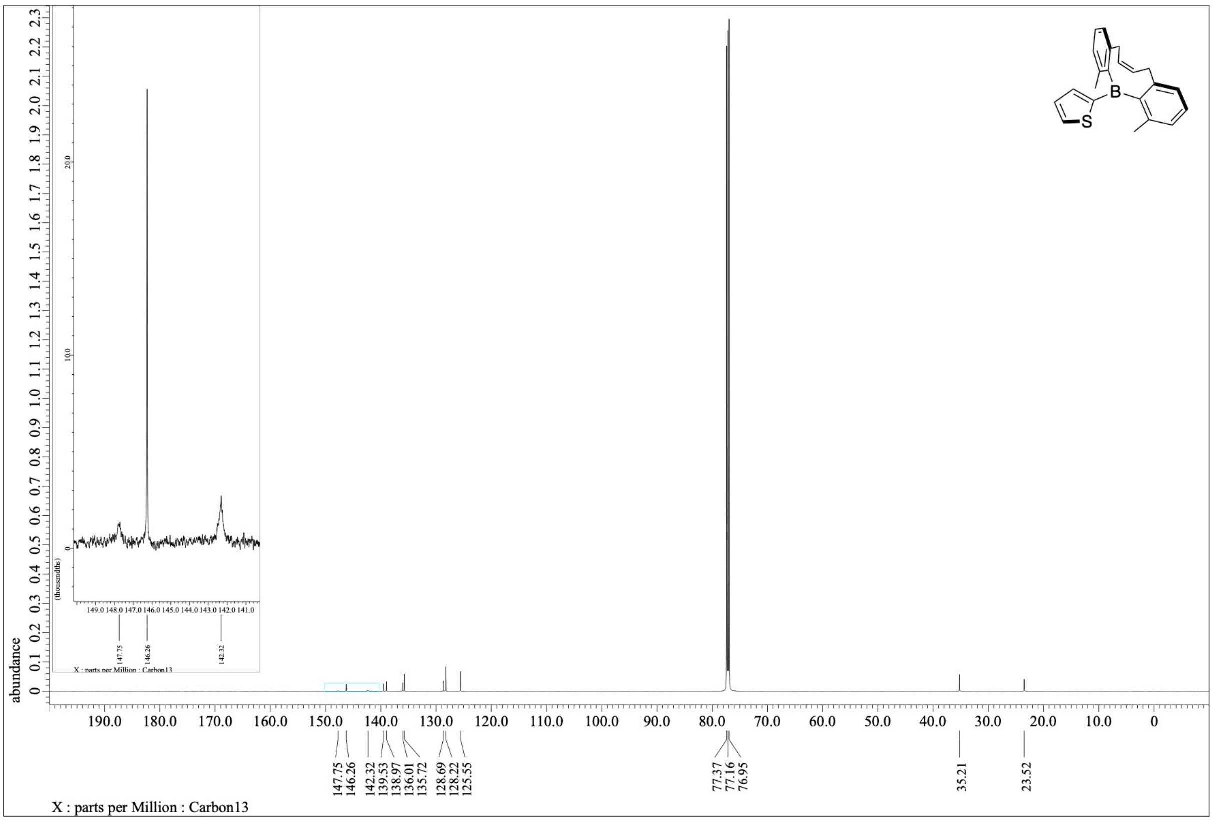

Figure S23. $^{13}C\{^{1}H\}$ NMR spectrum of **1** (150 MHz, $CDCl_3$).

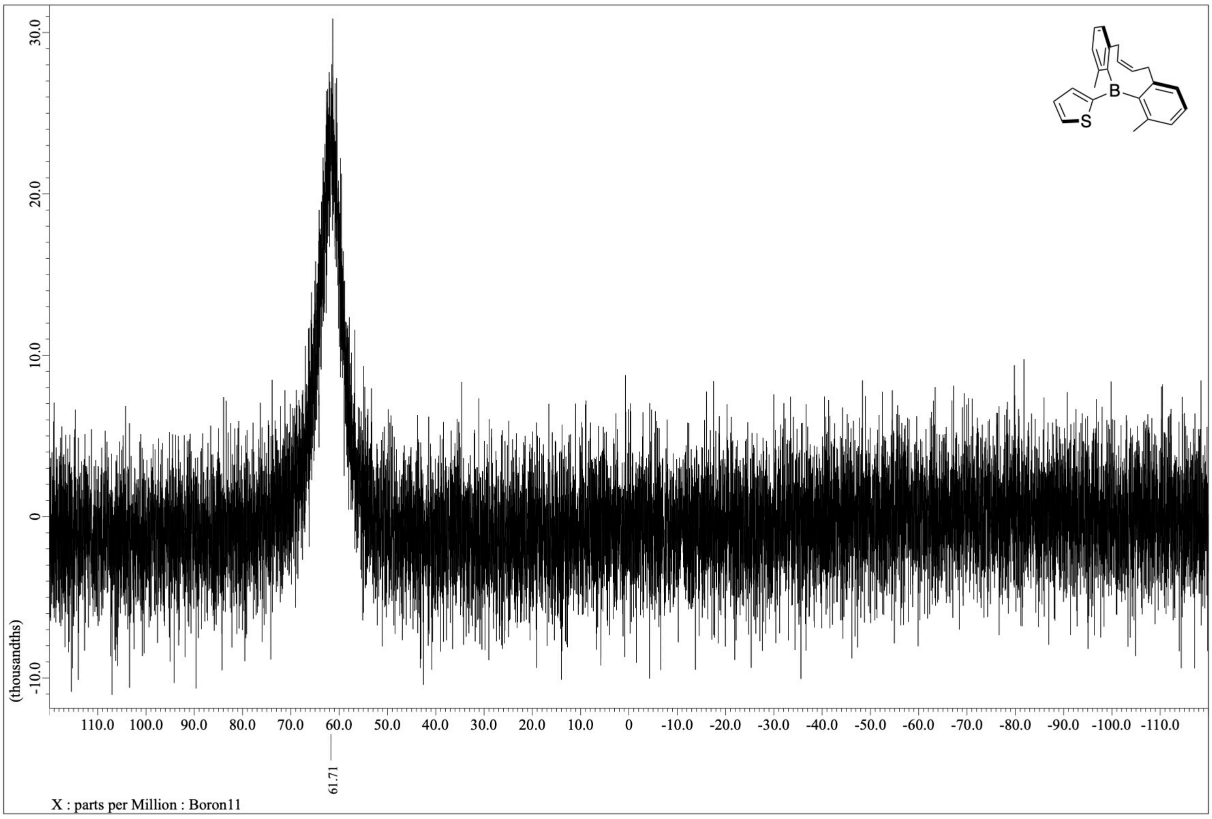

Figure S24. ^{11}B NMR spectrum of **1** (128 MHz, $CDCl_3$).

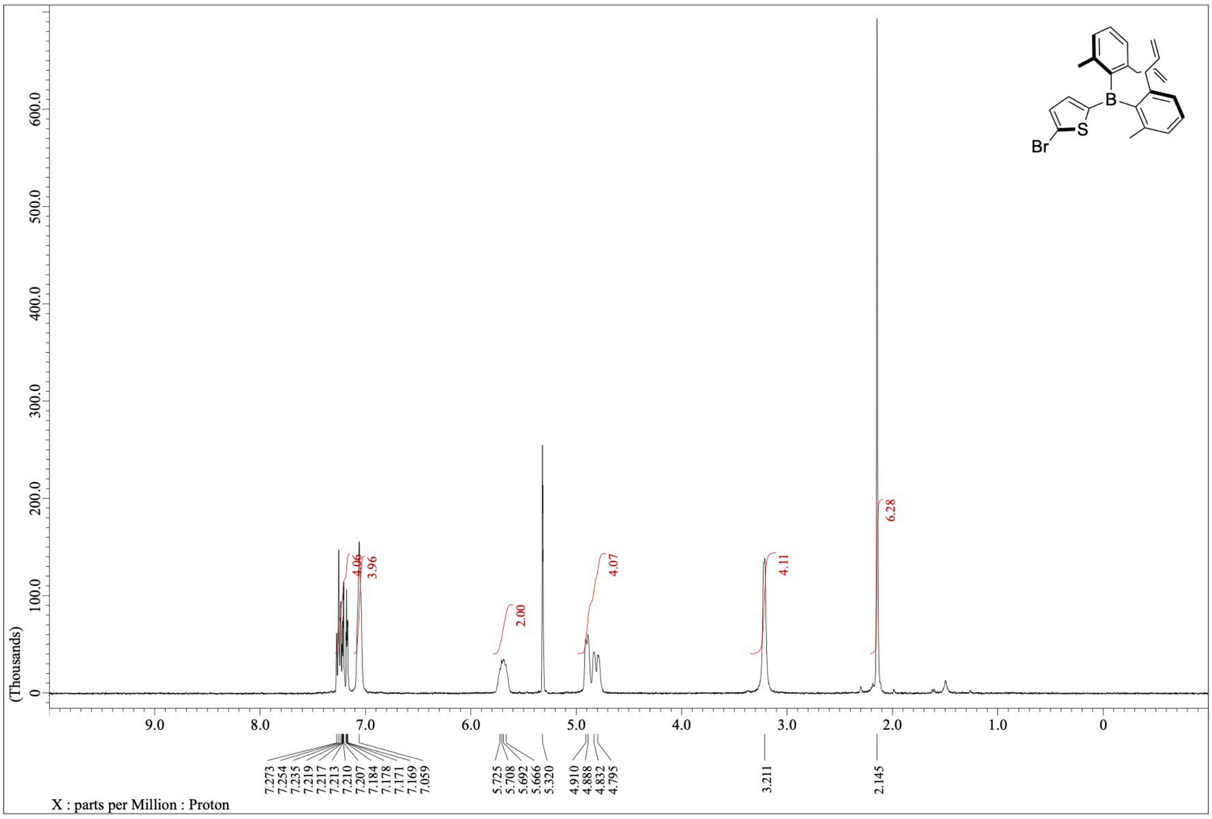

Figure S25. ^{1}H NMR spectrum of **6** (400 MHz, dichloromethane-d_2).

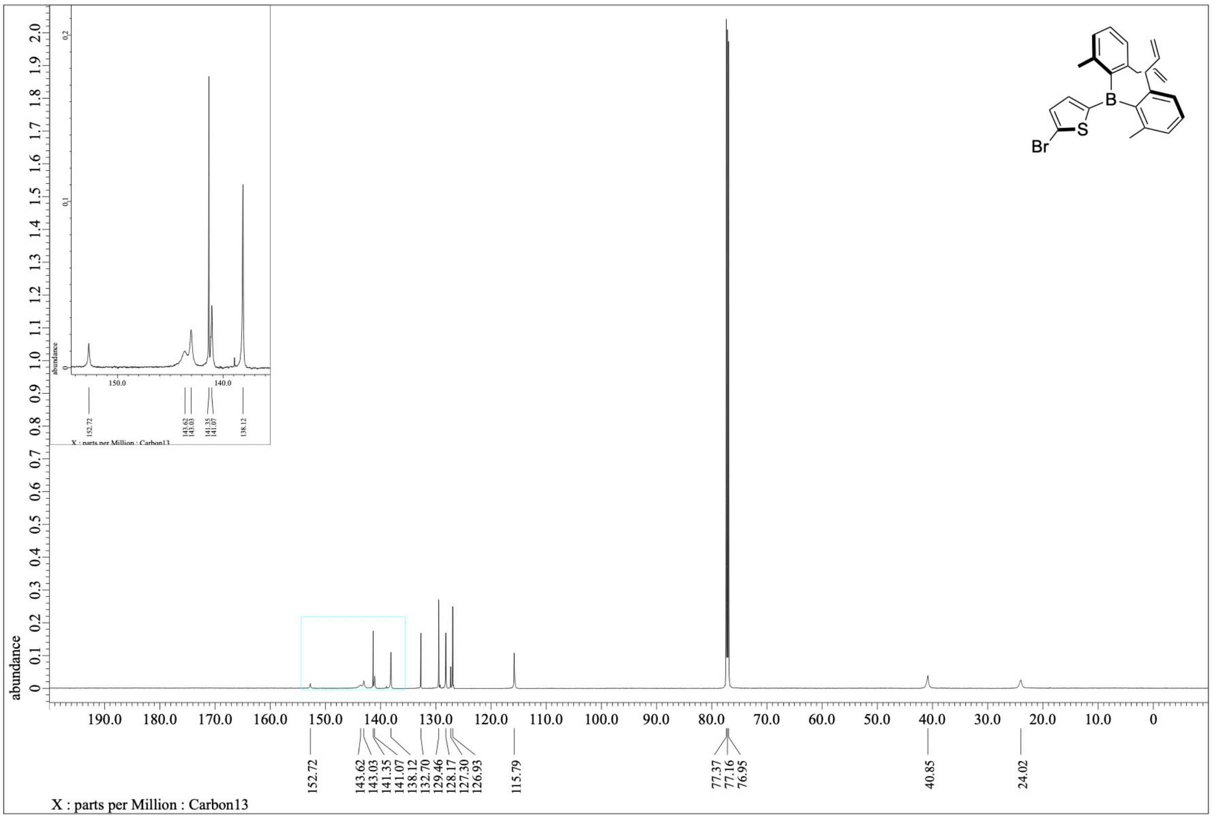

Figure S26. ^{13}C{^{1}H} NMR spectrum of **6** (150 MHz, $CDCl_3$).

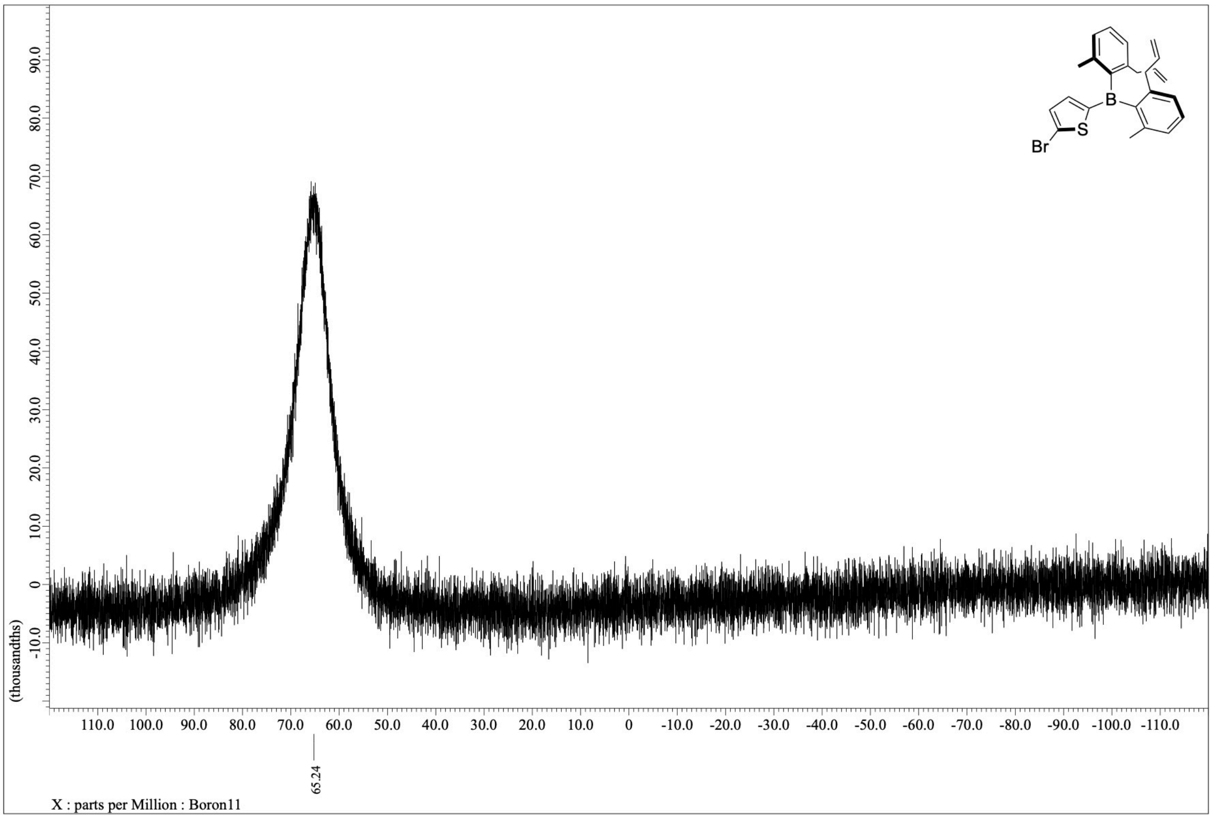

Figure S27. ^{11}B NMR spectrum of **6** (128 MHz, $CDCl_3$).

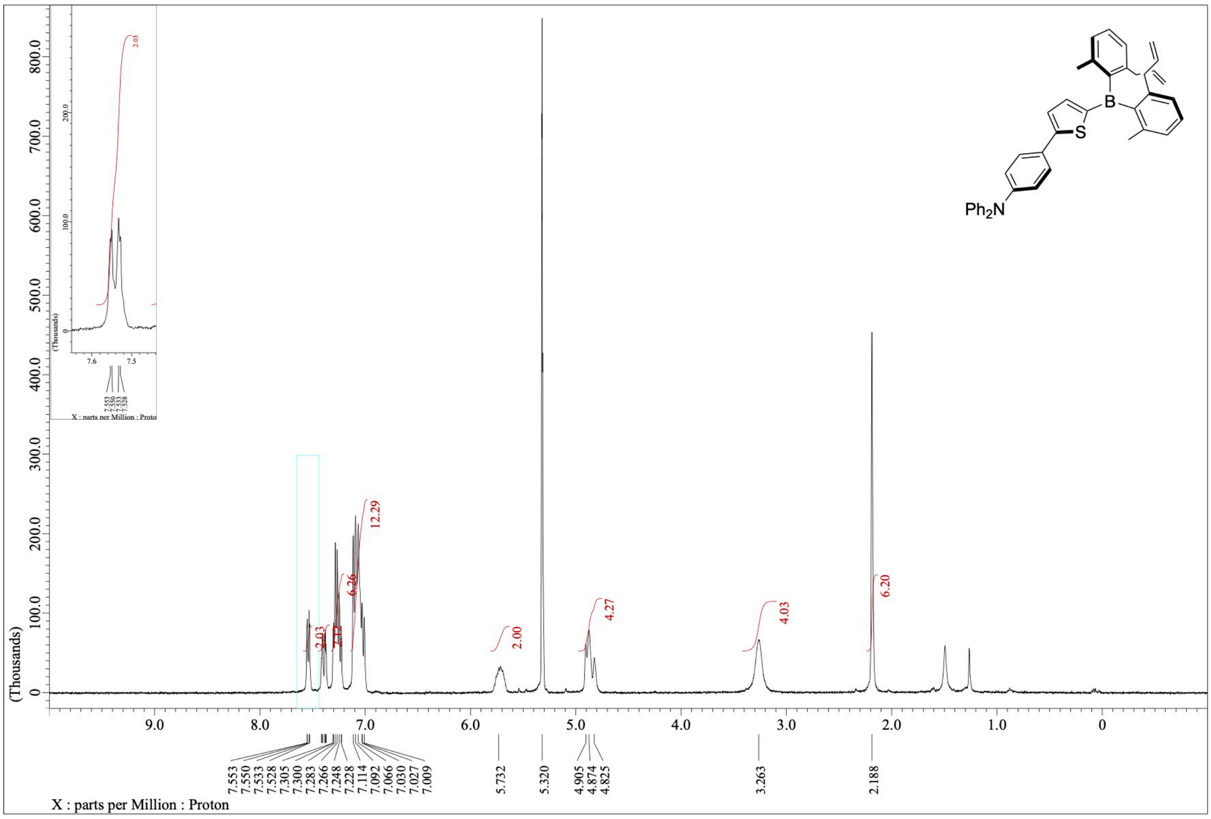

Figure S28. ^{1}H NMR spectrum of **8** (400 MHz, dichloromethane-d_2).

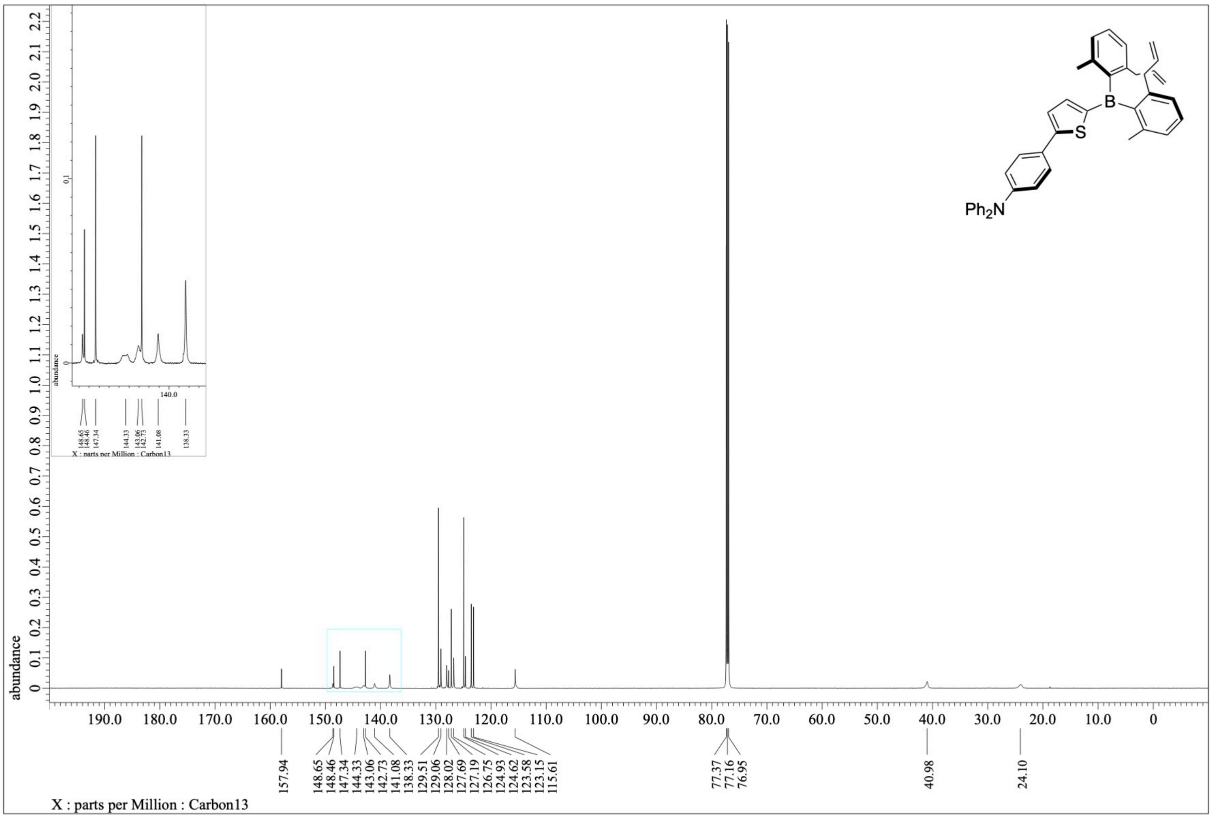

Figure S29. $^{13}C\{^{1}H\}$ NMR spectrum of **8** (150 MHz, $CDCl_3$).

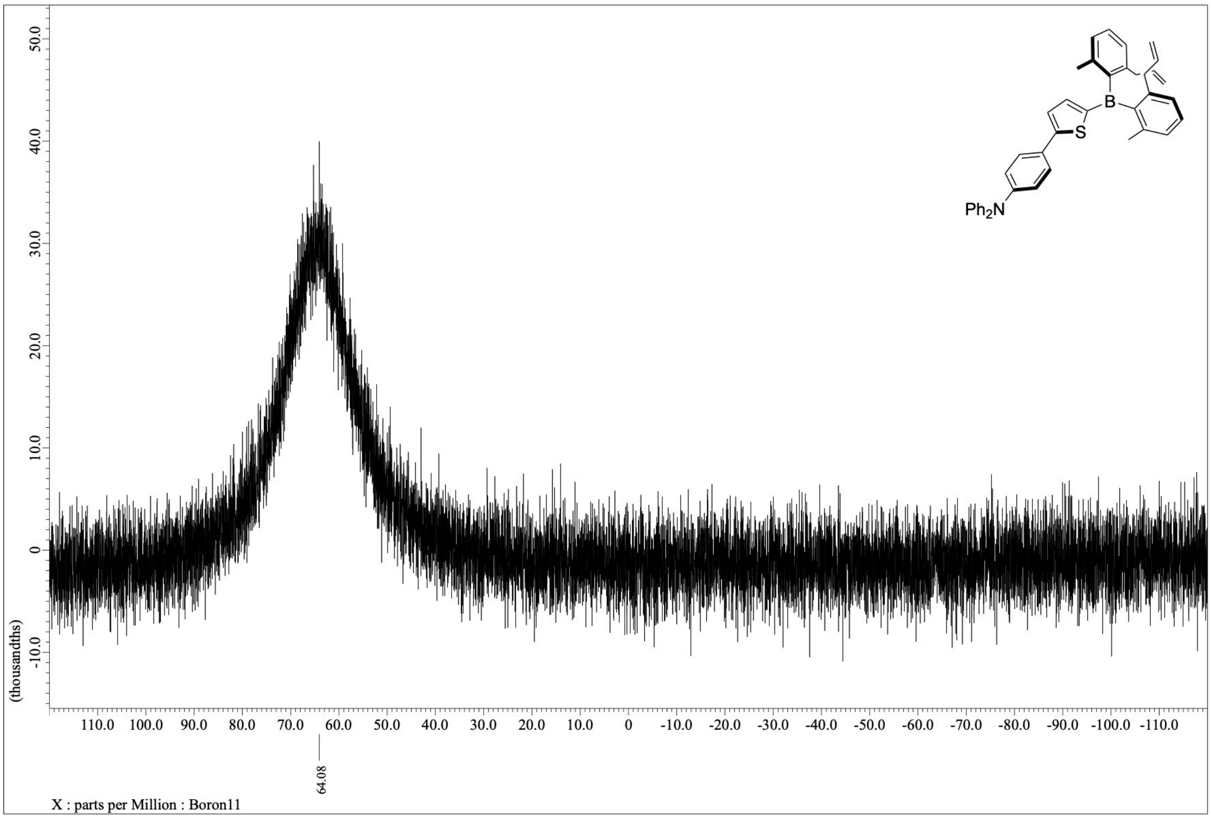

Figure S30. ^{11}B NMR spectrum of **8** (128 MHz, $CDCl_3$).

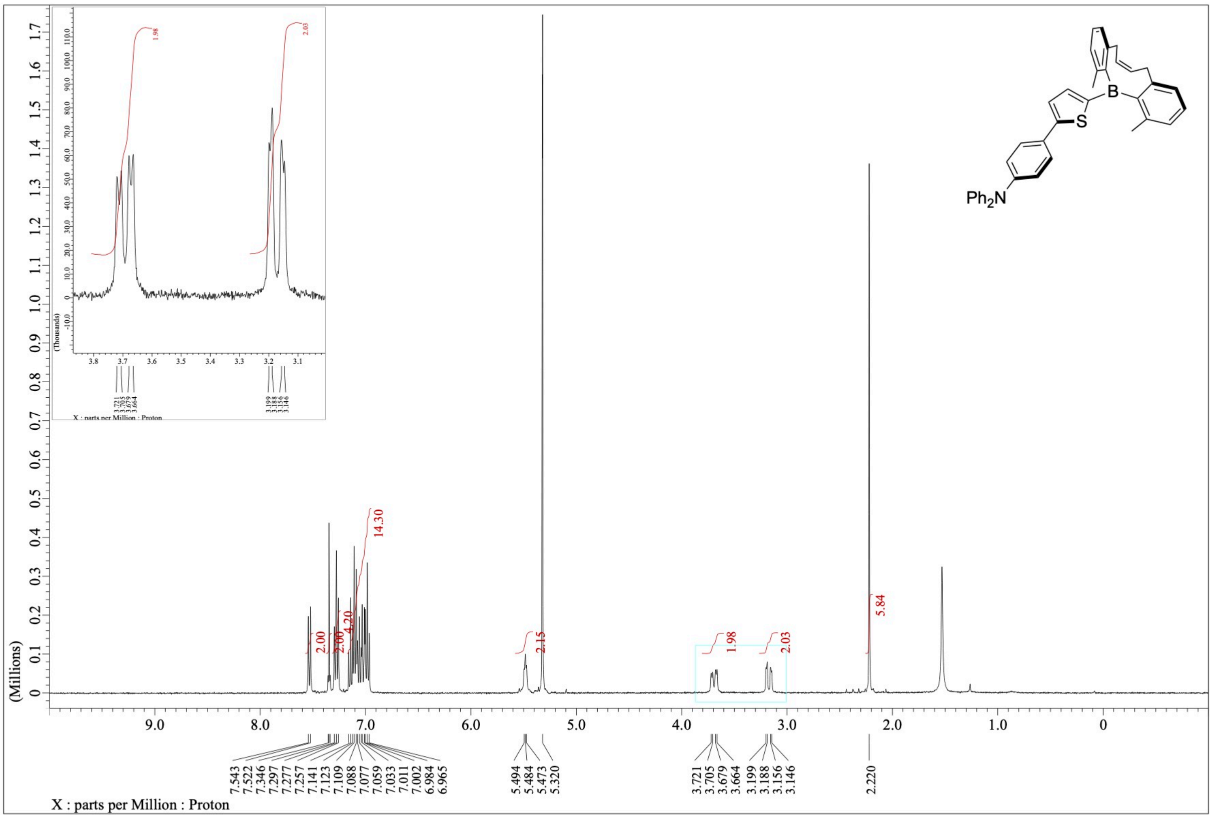

Figure S31. ^{1}H NMR spectrum of **2** (400 MHz, dichloromethane-d_2).

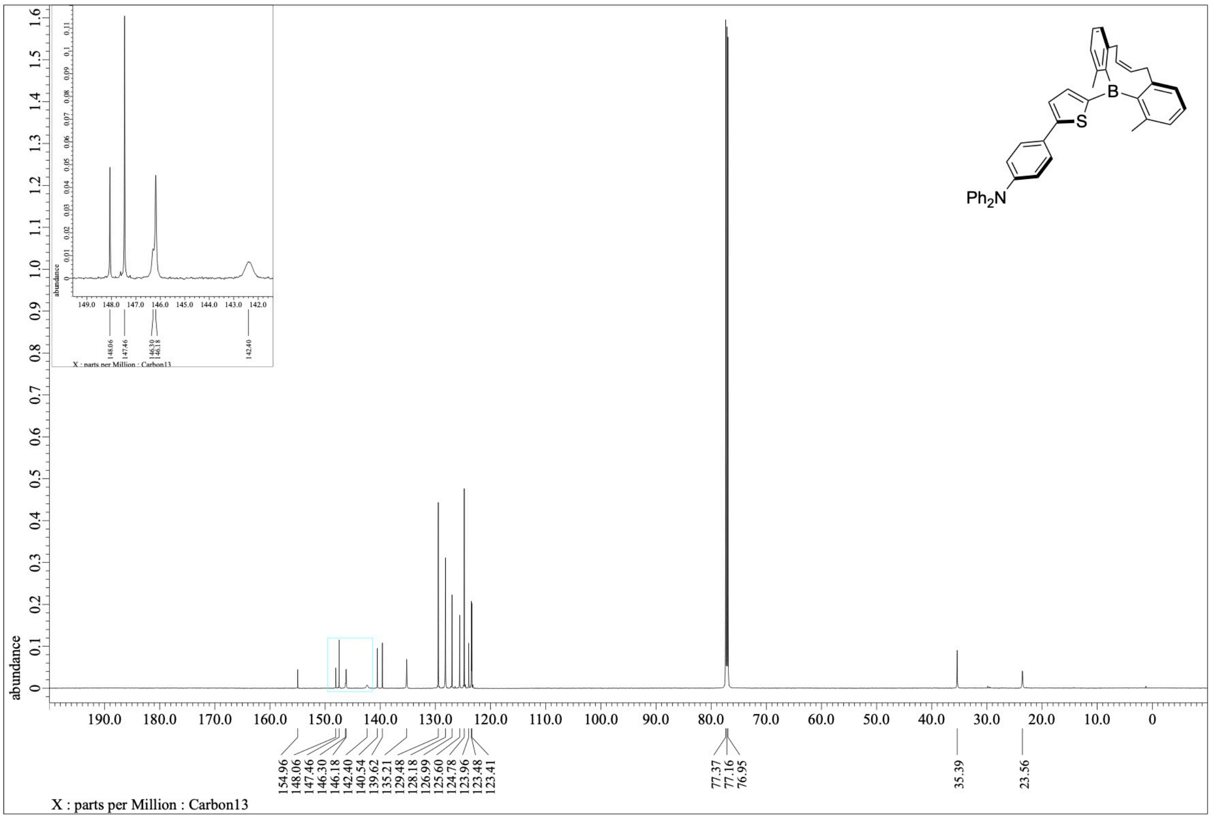

Figure S32. $^{13}C\{^{1}H\}$ NMR spectrum of **2** (150 MHz, $CDCl_3$).

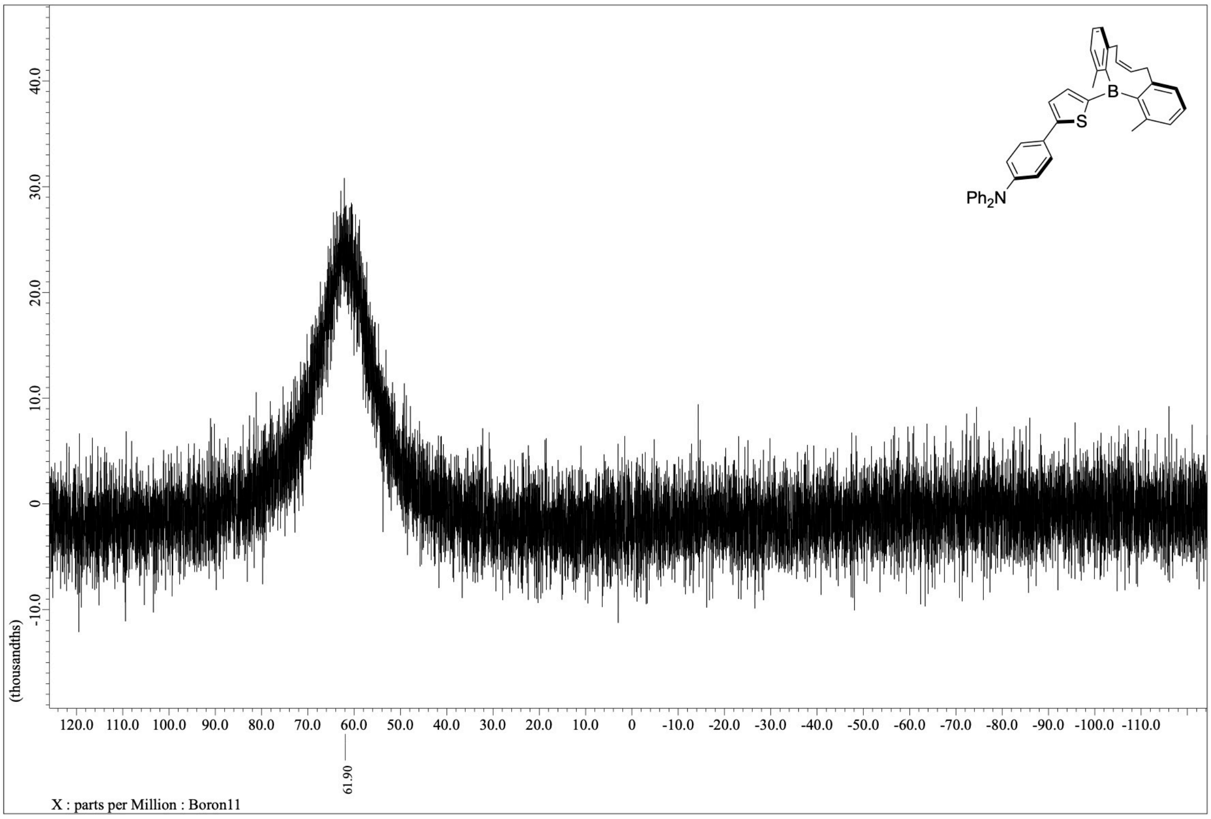

Figure S33. ^{11}B NMR spectrum of **2** (128 MHz, $CDCl_3$).

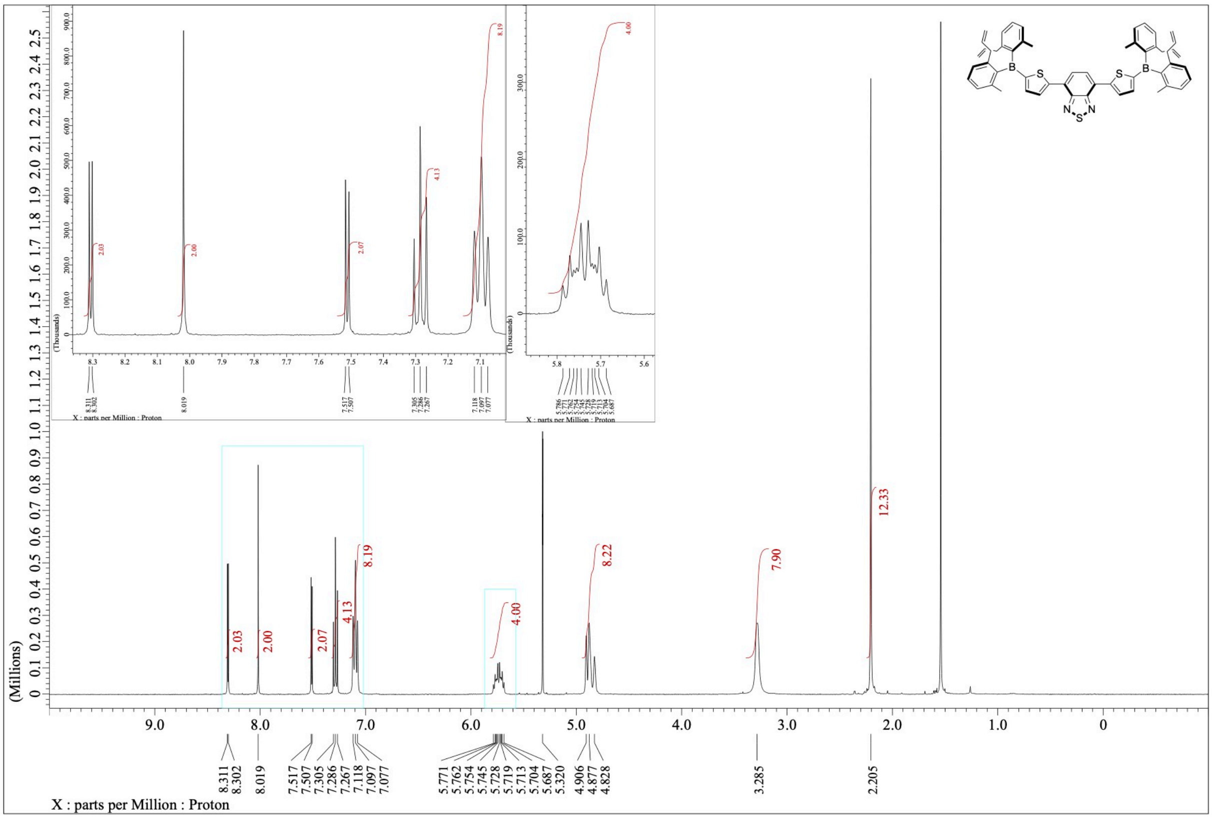

Figure S34. ^{1}H NMR spectrum of **9a** (400 MHz, dichloromethane-d_2).

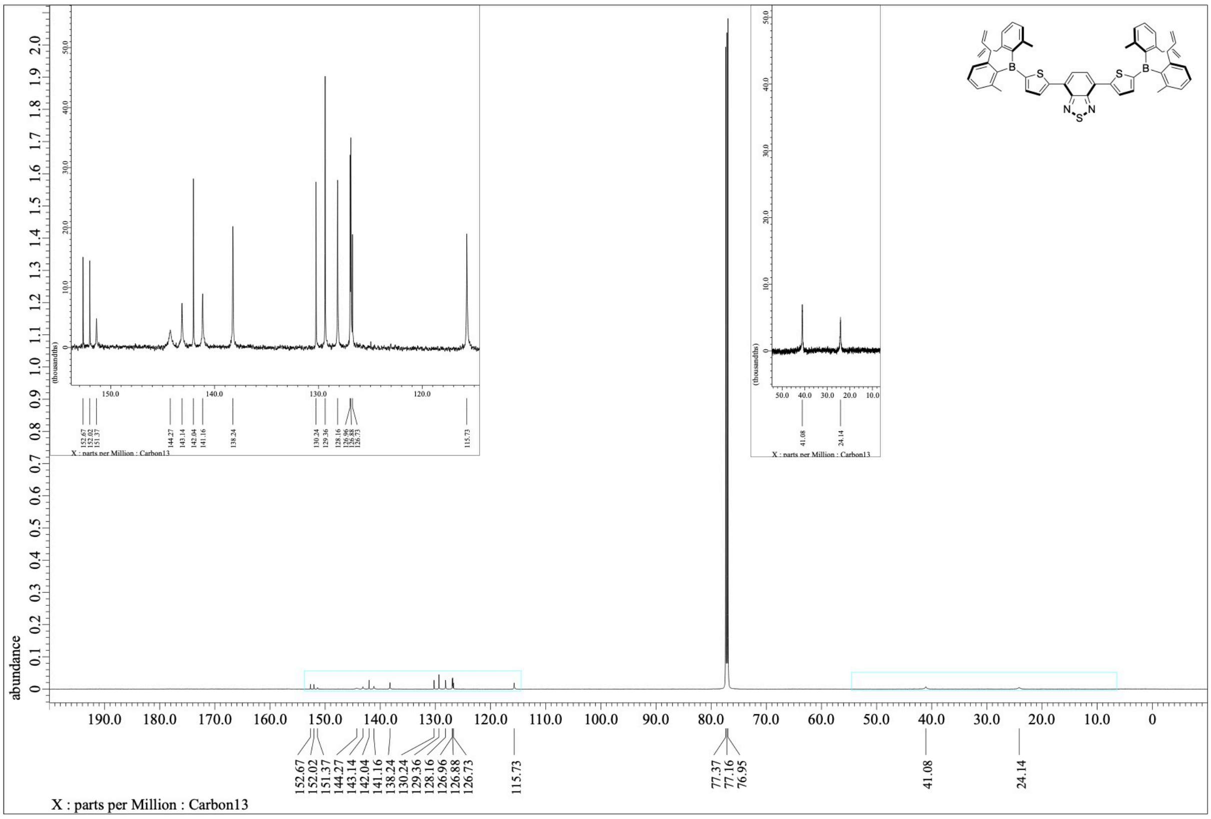

Figure S35. $^{13}C\{^1H\}$ NMR spectrum of **9a** (150 MHz, $CDCl_3$).

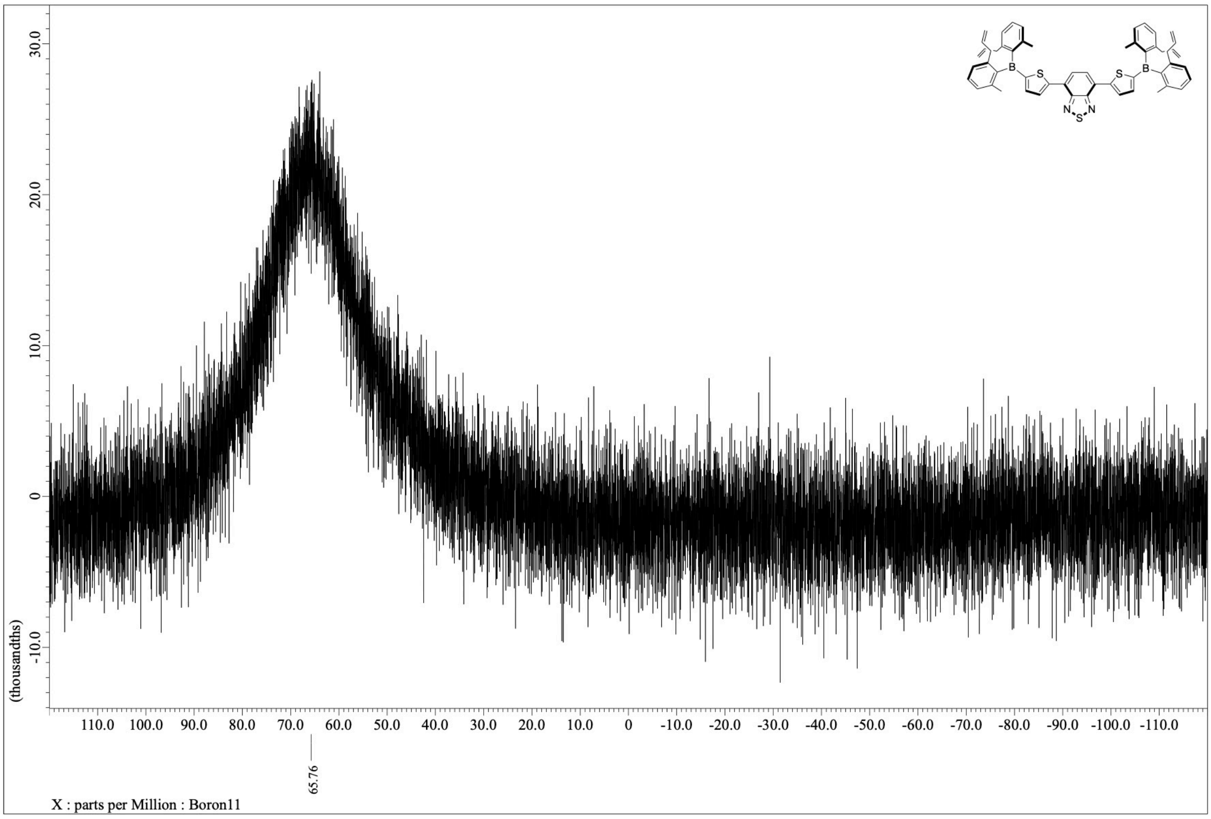

Figure S36. ^{11}B NMR spectrum of **9a** (128 MHz, $CDCl_3$).

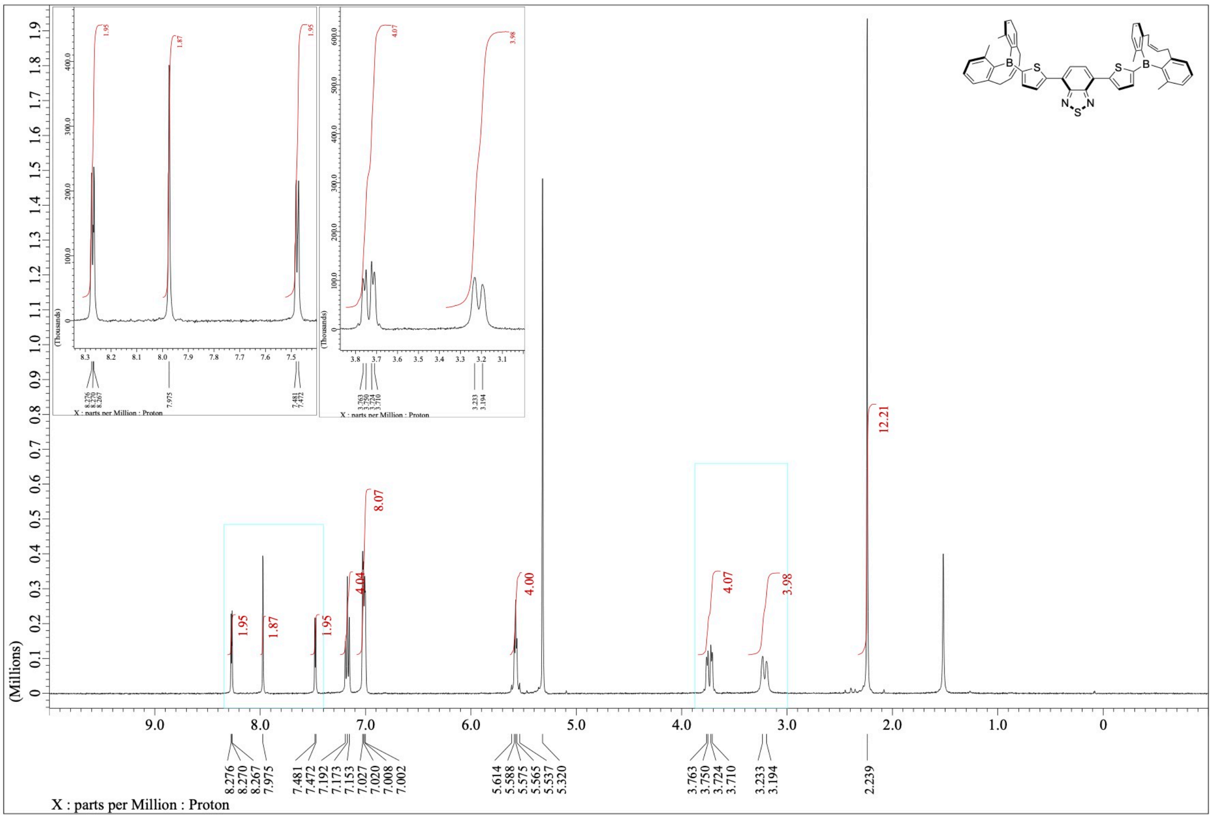

Figure S37. ^{1}H NMR spectrum of **3a** (400 MHz, dichloromethane-d_2).

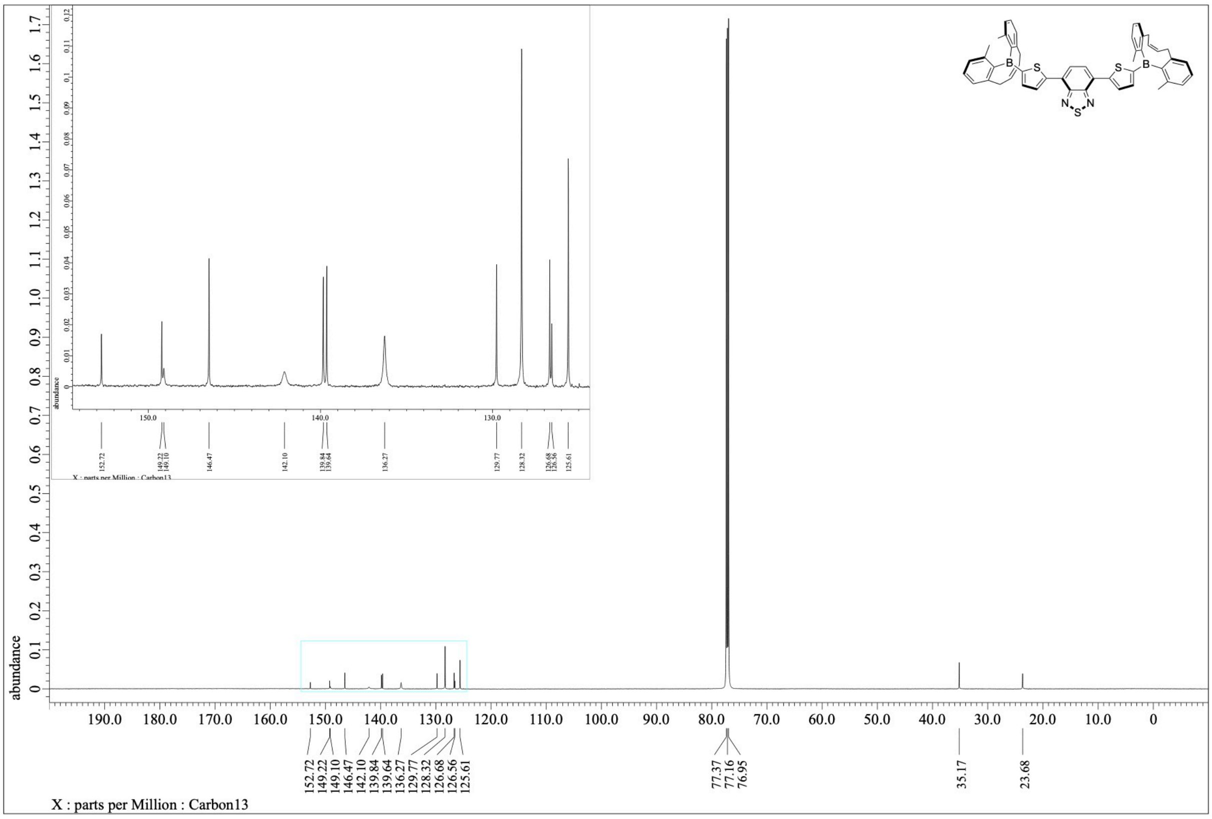

Figure S38. $^{13}C\{^{1}H\}$ NMR spectrum of **3a** (150 MHz, $CDCl_3$).

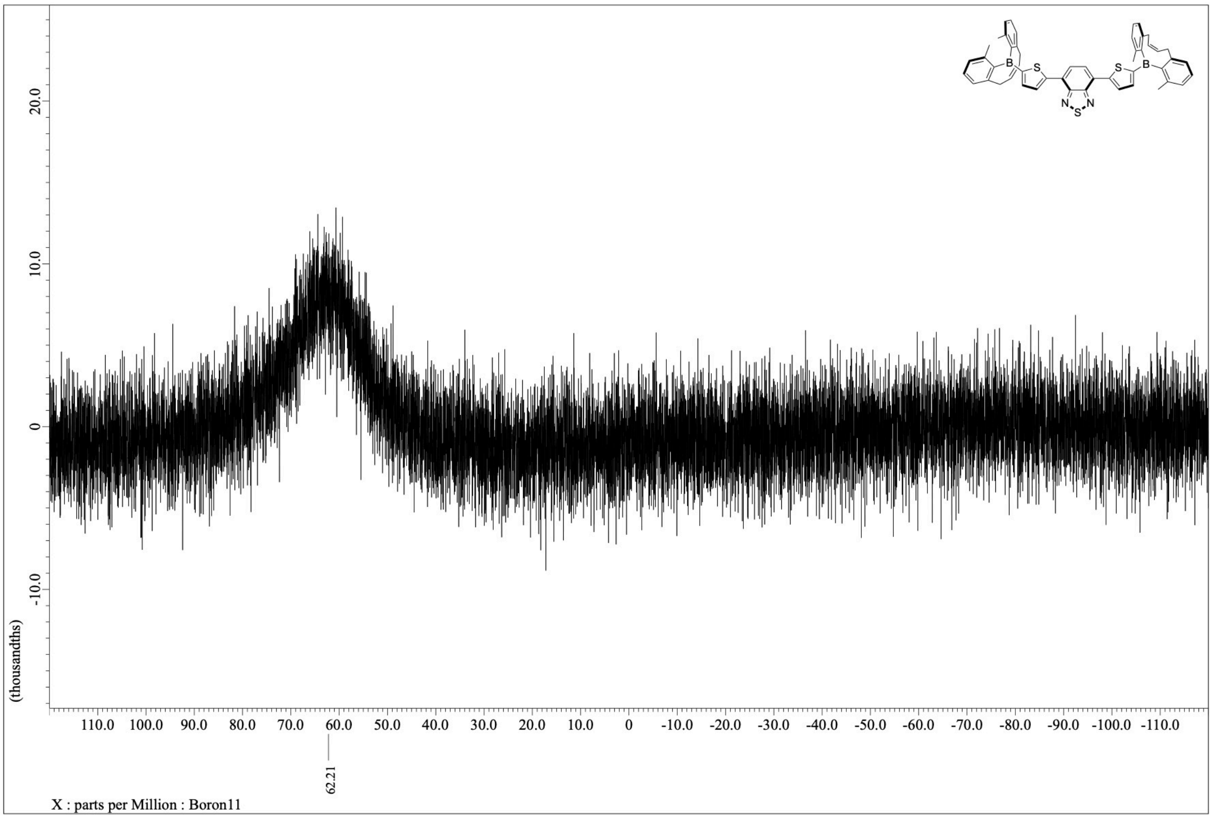

Figure S39. ^{11}B NMR spectrum of **3a** (128 MHz, $CDCl_3$).

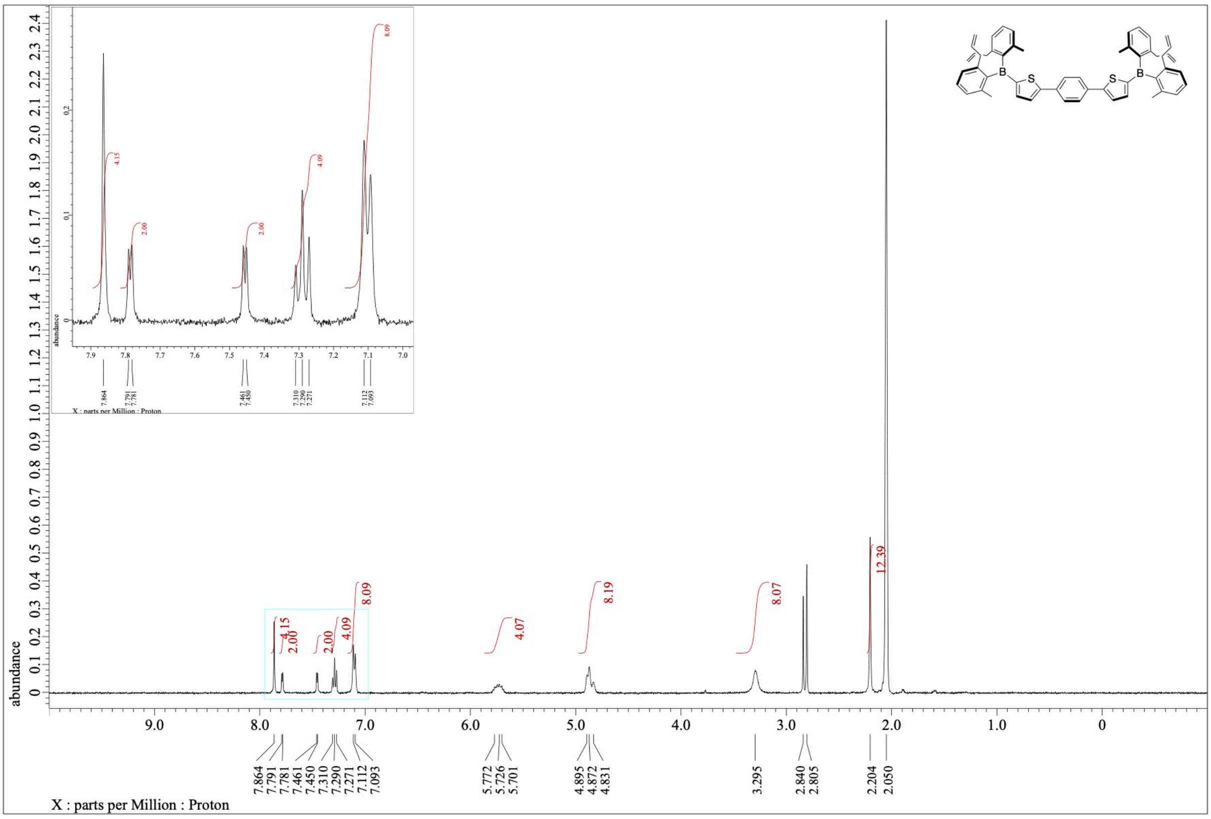

Figure S40. ^{1}H NMR spectrum of **9b** (400 MHz, acetone-d_6).

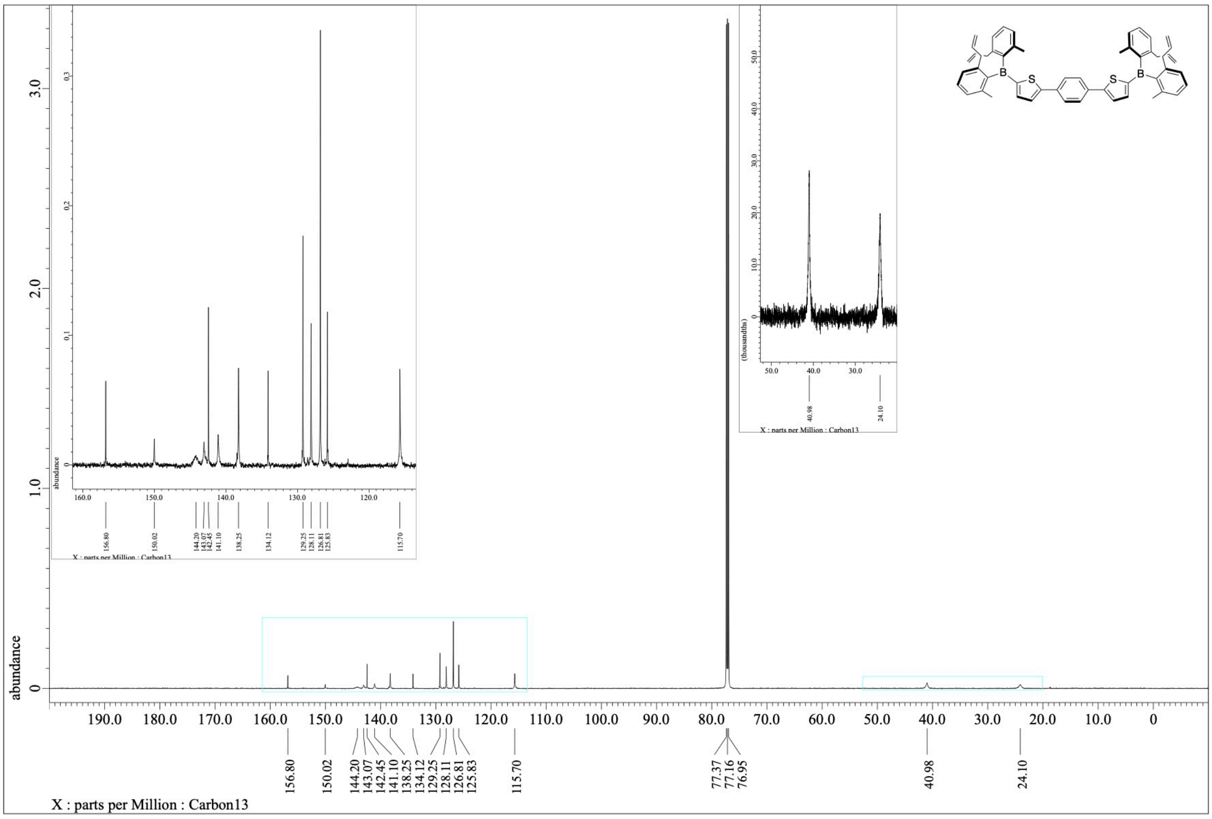

Figure S41. $^{13}C\{^1H\}$ NMR spectrum of **9b** (150 MHz, $CDCl_3$).

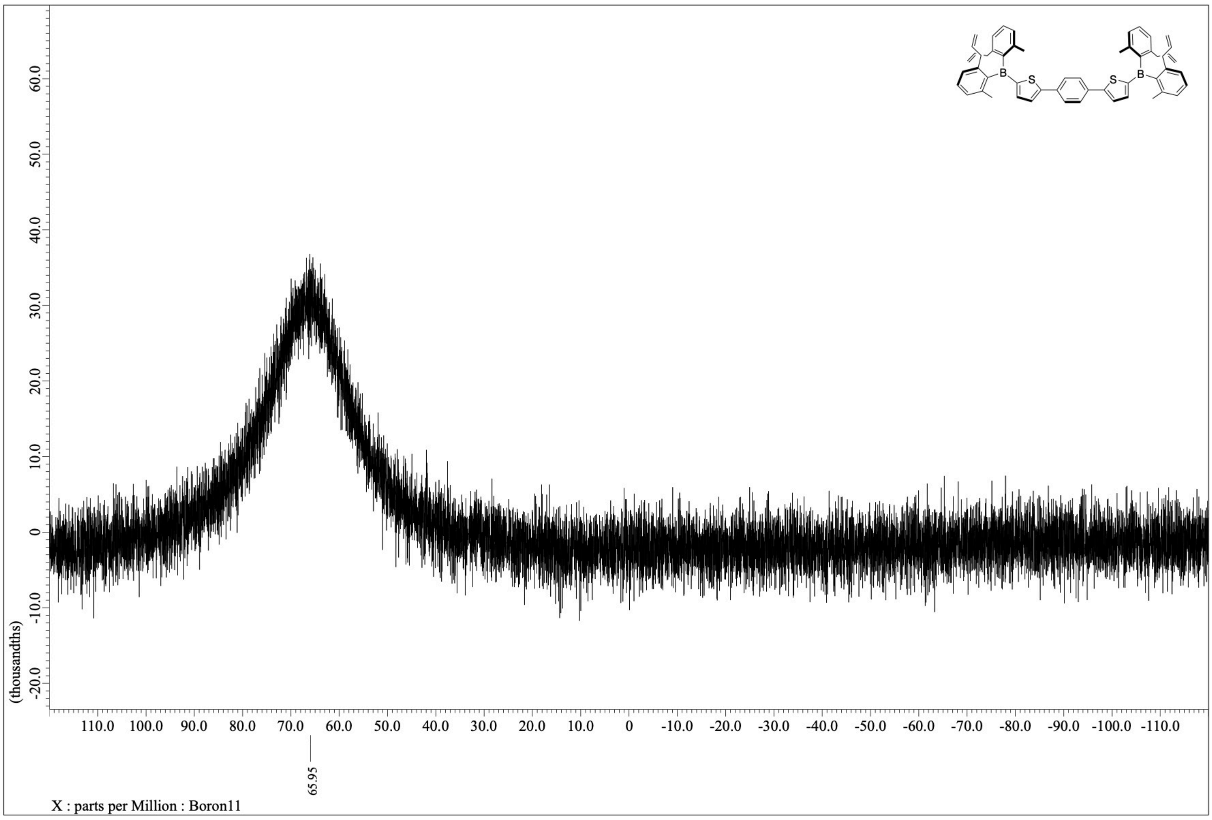

Figure S42. ^{11}B NMR spectrum of **9b** (128 MHz, $CDCl_3$).

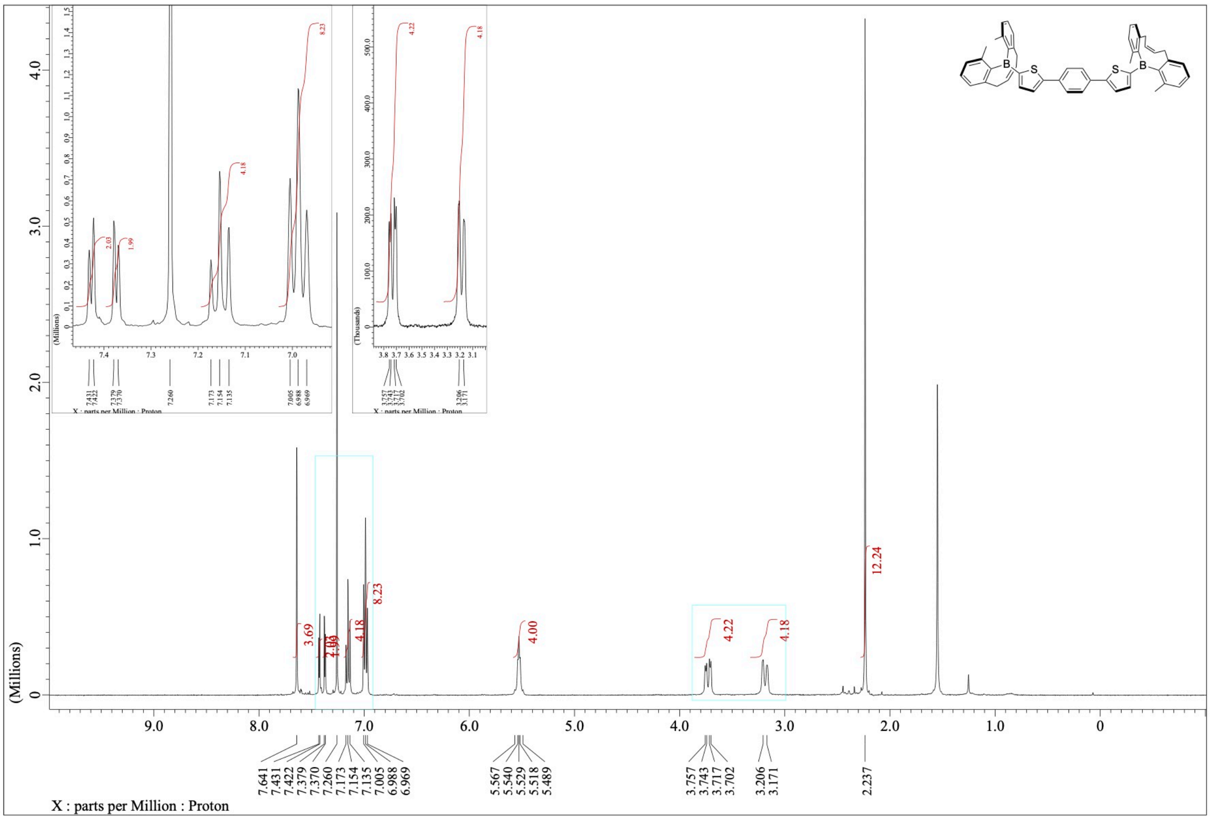

Figure S43. ^{1}H NMR spectrum of **3b** (400 MHz, $CDCl_3$).

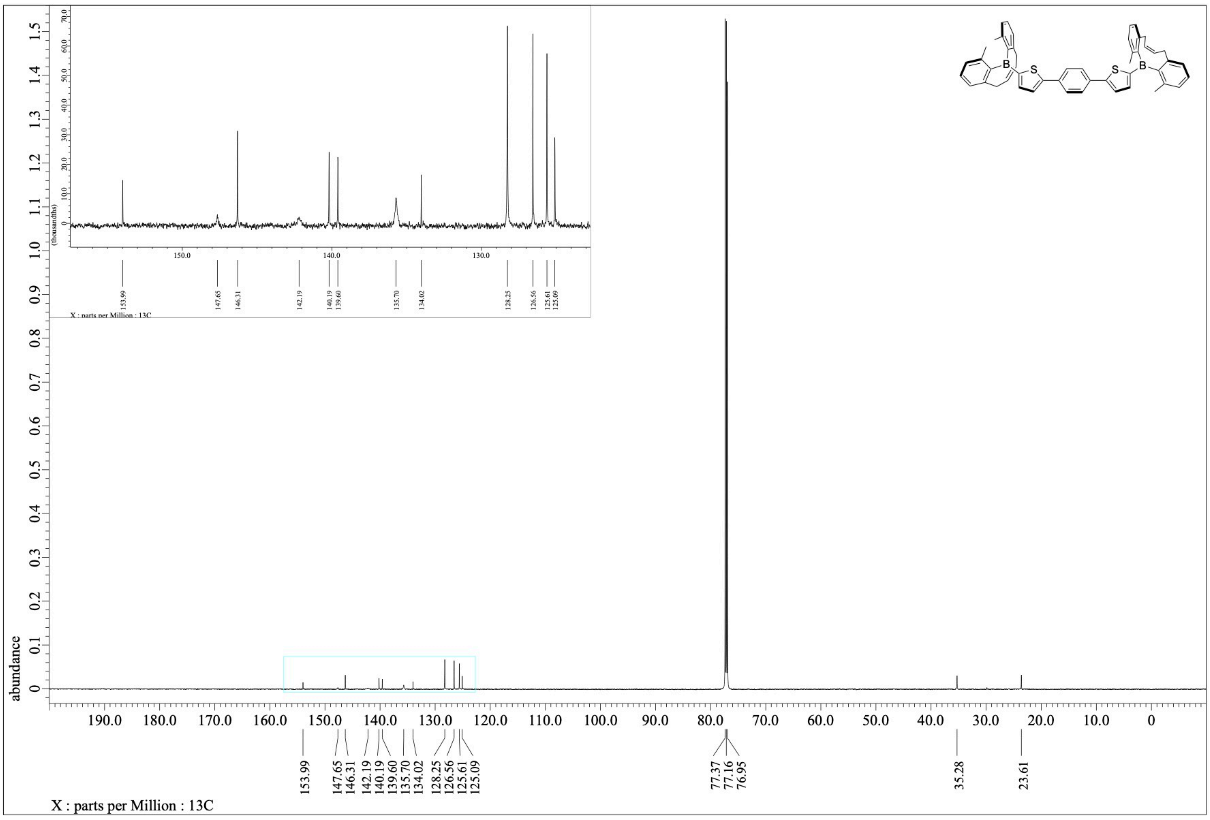

Figure S44. $^{13}C\{^{1}H\}$ NMR spectrum of **3b** (150 MHz, $CDCl_3$).

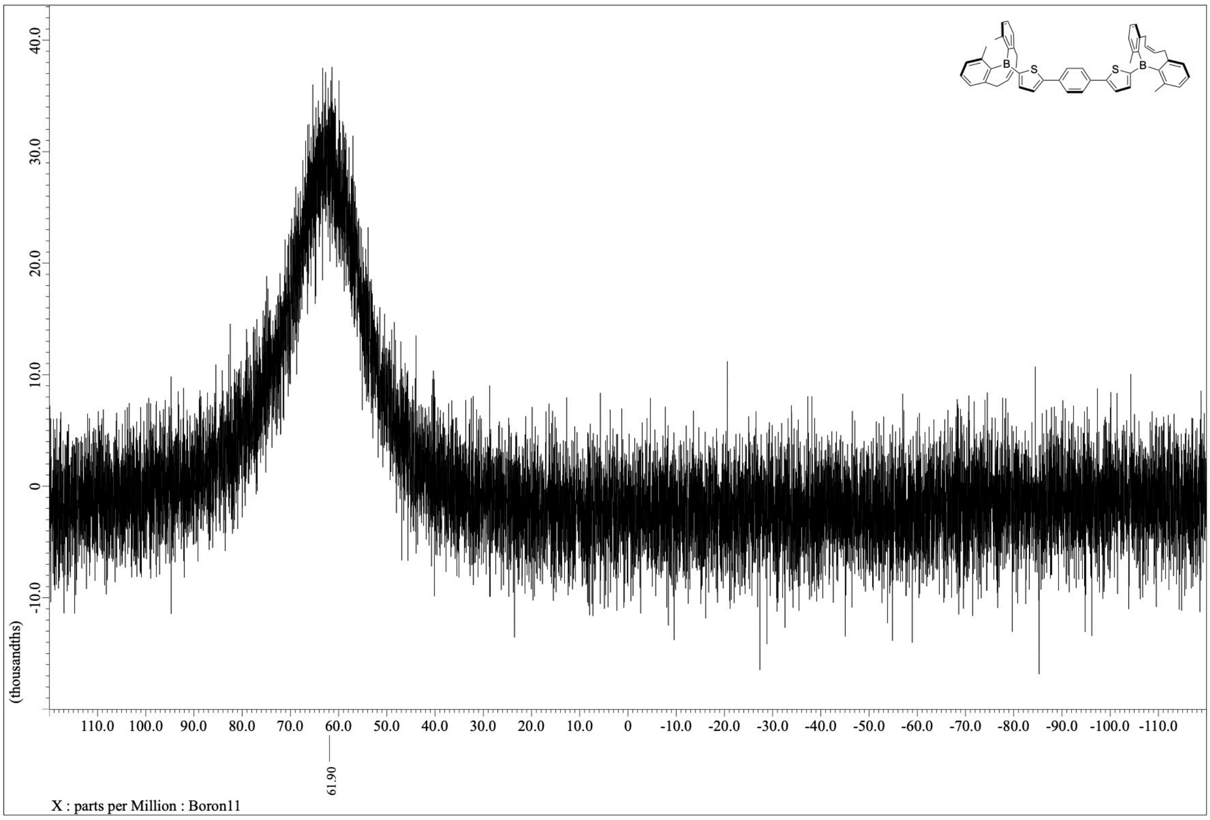

Figure S45. ^{11}B NMR spectrum of **3b** (128 MHz, $CDCl_3$).
